# Antibiotic resistomes discovered in the gut microbiomes of Korean swine and cattle

**DOI:** 10.1093/gigascience/giaa043

**Published:** 2020-05-05

**Authors:** Suk-Kyung Lim, Dongjun Kim, Dong-Chan Moon, Youna Cho, Mina Rho

**Affiliations:** 1 Animal and Plant Quarantine Agency, Bacterial Disease Division, 177 Hyeoksin 8-ro, Gimcheon-si, Gyeongsangbuk-do, 39660, Republic of Korea; 2 Hanyang University, 222 Wangsimni-ro, Seongdong-gu, Seoul, 04763, Republic of Korea, Department of Computer Science and Engineering; 3 Hanyang University, 222 Wangsimni-ro, Seongdong-gu, Seoul, 04763, Republic of Korea, Department of Biomedical Informatics

**Keywords:** swine gut microbiomes, cattle gut microbiomes, antibiotic resistome, antibiotic resistance gene

## Abstract

**Background:**

Antibiotics administered to farm animals have led to increasing prevalence of resistance genes in different microbiomes and environments. While antibiotic treatments help cure infectious diseases in farm animals, the possibility of spreading antibiotic resistance genes into the environment and human microbiomes raises significant concerns. Through long-term evolution, antibiotic resistance genes have mutated, thereby complicating the resistance problems.

**Results:**

In this study, we performed deep sequencing of the gut microbiomes of 36 swine and 41 cattle in Korean farms, and metagenomic analysis to understand the diversity and prevalence of antibiotic resistance genes. We found that aminoglycoside, β-lactam, lincosamide, streptogramin, and tetracycline were the prevalent resistance determinants in both swine and cattle. Tetracycline resistance was abundant and prevalent in cattle and swine. Specifically, *tetQ, tetW, tetO, tet32*, and *tet44* were the 5 most abundant and prevalent tetracycline resistance genes. Their prevalence was almost 100% in swine and cattle. While *tetQ* was similarly abundant in both swine and cattle, *tetW* was more abundant in swine than in cattle. Aminoglycoside was the second highest abundant resistance determinant in swine, but not in cattle. In particular, *ANT(6)* and *APH(3′′)* were the dominant resistance gene families in swine. β-lactam was also an abundant resistance determinant in both swine and cattle. *Cfx* was the major contributing gene family conferring resistance against β-lactams.

**Conclusions:**

Antibiotic resistome was more pervasive in swine than in cattle. Specifically, prevalent antibiotic resistance genes (prevalence >50%) were found more in swine than in cattle. Genomic investigation of specific resistance genes from the gut microbiomes of swine and cattle in this study should provide opportunities to better understand the exchange of antibiotic resistance genes in farm animals.

## Background

Antibiotics have been widely used to cure infectious diseases. In farms, antibiotics have also been used to treat and prevent diseases or to promote the growth of animals. Increasing administration of antibiotics expedites the development of resistance and leads to the spread of resistance genes in the farm environment and human population [1]. Moreover, gene transfer from the environment or food chain to the human population further complicates this problem. In particular, antibiotic resistance genes have been transferred from one bacterium to others within the human microbiome, and between human and livestock microbiomes [2].

With advances in high-throughput sequencing technology and metagenomic analysis, gut microbiomes have been investigated to understand the prevalence of antibiotic resistance genes (ARGs) and the compositional changes in the microbiome after treatment. In recent years, ARGs have been extensively studied to understand their diversity and abundance in the human microbiome in terms of race and age [3, 4]. The most prevalent resistance determinant in humans is tetracycline [4], which is also prevalent in farm animals [5]. Because tetracycline is widely applied for infection control and growth promotion, several studies have suggested a positive correlation between its use and prevalence [6]. In human skin microbiomes [7] and soil microbiomes [8], divergent ARGs that show low sequence similarity to the known genes have also been identified through functional metagenomics, implying that resistance genes have evolved in diverse environments.

For the gut microbiomes of farm animals, several studies have explored the prevalence of antibiotic resistance genes [5, 9]. A recent study on ARGs in Chinese, French, and Danish swine showed that the most prevalent classes of ARGs are tetracycline, β-lactam, macrolide, streptogramin, and bacitracin [10]. Notably, the profile of ARGs in Chinese swine was different from that in the other 2 populations, in terms of the composition and abundance. Tetracycline, aminoglycoside, and β-lactam were also the abundant antibiotic classes in the farm environments for swine [5], which was consistent with the ARG profiles of farm animals. The effects of antibiotics, used as feed additives, on the changes in bacterial composition have been discussed. In the cattle microbiome, it was found that tetracycline was the most abundant class, followed by aminoglycoside [11]. A previous study suggested that ARGs in animal microbiomes could be transferred and distributed to other environments [12].

In this work, we performed metagenomic analysis on the gut microbiomes of swine and cattle to investigate the diversity and prevalence of ARGs in different farm environments. An unbiased screening of microbial resistance genes was performed using metagenomic shotgun sequencing data. To our knowledge, this is the first study investigating ARGs in multiple types of farm animals raised in Korea. We observed the presence of 2 different patterns of resistance genes: 1 type is host-dedicated; the other exists in different host animals.

## Data Description

We performed deep sequencing on the gut microbiomes of 36 swine and 41 cattle from Korean farms (Supplementary Table S1), and metagenomic analysis to understand the diversity and prevalence of ARGs. All raw sequencing data described in this study are available at the European Nucleotide Archive with the accession number PRJEB32496.

Fresh fecal samples from healthy finishing swine and adult Korean cattle were collected aseptically in 25 feedlots throughout Korea between August 2017 and June 2018 (Supplementary Tables S2 and S3), following the guidelines of the Animal Protection Act of the Animal and Plant Quarantine Agency. Farm selection was based on 2 criteria: geographical distribution and farm size.

The Illumina HiSeq4000 Platform (Illumina, San Diego, CA, USA) was used to sequence the DNA samples. A total of 77 gut microbiomes were sequenced from swine and cattle for this study. For every sample, 151-bp paired-end sequences were generated from the insert of 350 bp. An average of 38 M paired reads (ranging between 25 M and 75 M) were generated for each sample after filtering.

## Analysis

### Bacterial composition of swine and cattle gut microbiomes

**Figure 1:**
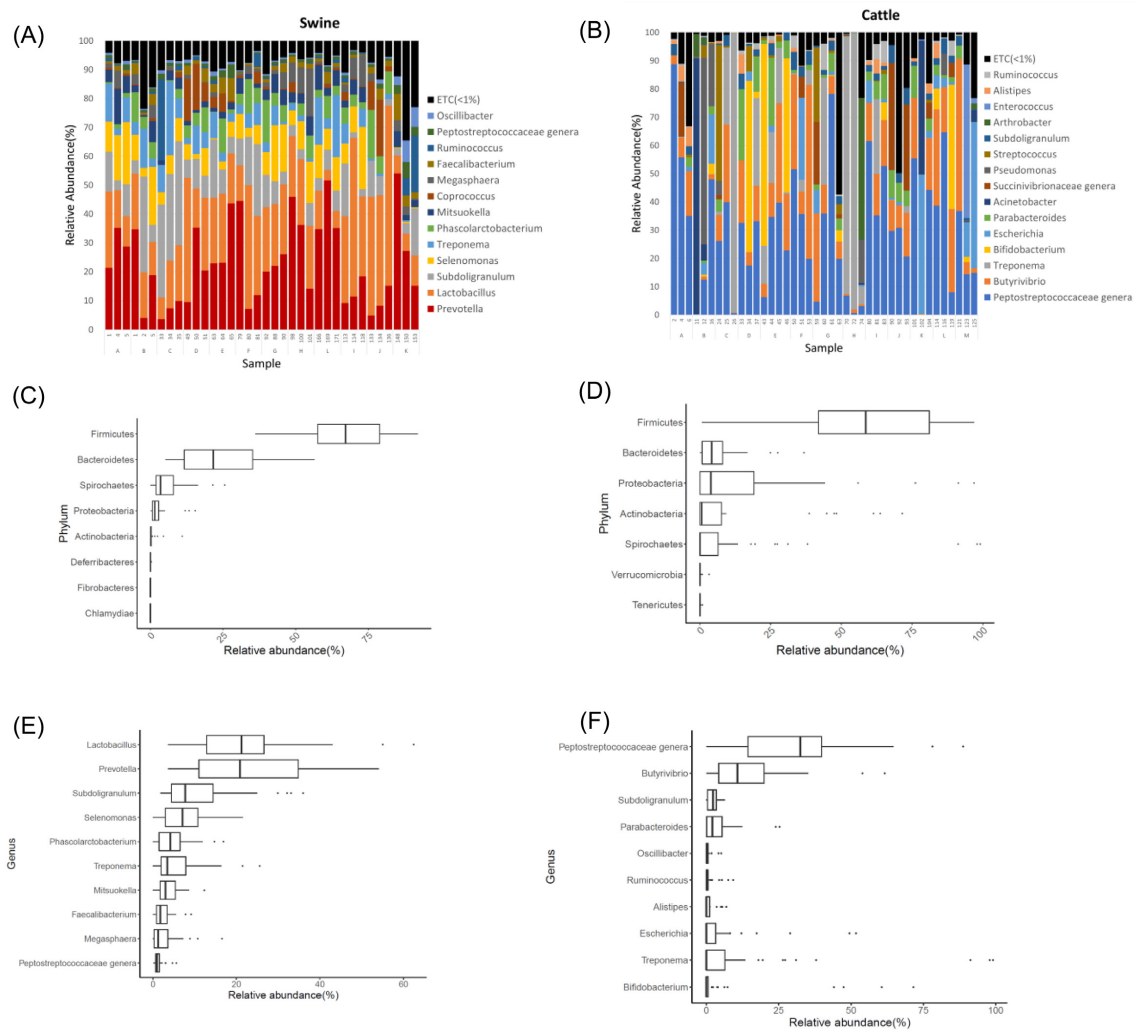
Bacterial composition of swine and cattle gut microbiomes. Genus-level bacterial composition in (A) swine and (B) cattle. The 10 most abundant bacterial phyla in (C) swine and (D) cattle. The 10 most abundant bacterial genera in (E) swine and (F) cattle. Boxplots display the median as the middle line whilst the perimeters of the box display the 1st and 3rd quantiles of the data.The whiskers span the range of the 25% quantile or the 75% quantile plus 1.5 times the interquartile range, and dots are outliers.

A total of 36 gut microbiomes from swine and 41 from cattle were collected to investigate their bacterial composition. Consistent with previous studies [13–16], the major phyla in swine and cattle were Bacteroidetes and Firmicutes, which were also commonly observed in human gut microbiomes [17]. Their proportions, however, were quite different in the 2 animals: 21.65% and 67.16%, respectively, in swine; 4.15% and 58.63%, respectively, for cattle (Fig. 1C and D). The ratio of Bacteroidetes to Firmicutes was much higher in swine than in cattle.

In the swine gut microbiomes, the major genera were *Lactobacillus* (21.19%; median proportion), *Prevotella* (20.89%), *Subdoligranulum* (7.75%), and *Selenomonas* (7.06%) (Fig. 1A and E). *Prevotella* was the major genus in Bacteroidetes, whereas *Lactobacillus, Subdoligranulum*, and *Selenomonas* were the major genera in Firmicutes. The proportion of *Prevotella* showed a negative correlation with that of *Subdoligranulum* (*r* = −0.6457; Fig. 1A and Supplementary Fig. S1A). At the species level, *Prevotella copri* was the most abundant species, which made up 17.23% of the microbiomes (Supplementary Fig. 2C). This value was higher than that of other species, such as *Lactobacillus amylovorus* (7.80%), *Subdoligranulum* species (7.75%), and *Streptococcus bovis* (7.06%).

In the cattle microbiomes, the major genera were the *Peptostreptococcaceae* genus (32.56%; median proportion) and *Butyrivibrio* (10.77%), which were observed in all cattle samples. Even though *Treponema* and *Bifidobacterium* were observed as 2 of the 10 most abundant genera ordered on the basis of their average proportions, their prevalence was <50%; i.e., they were seen in 19 and 14 of the 41 cattle samples, respectively. In several samples, high proportions of *Treponema* and *Bifidobacterium* were observed (Fig. 1B). *Treponema* was the most common bacterial genus in swine and cattle gut microbiomes. Its prevalence was 46.34% in cattle and 97.22% in swine.

**Figure 2:**
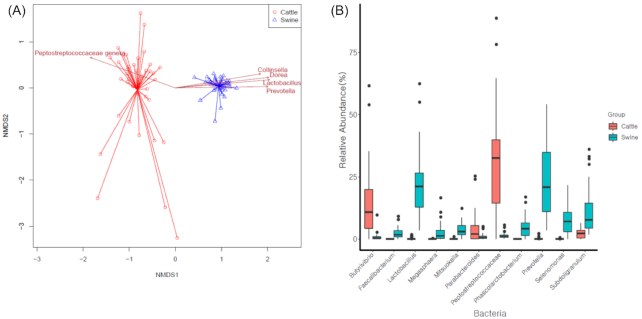
Different bacterial compositions in the gut microbiomes of swine and cattle. (A) Nonmetric multidimensional scaling (NMDS) analysis of genus-level bacterial composition in swine and cattle. (B) Differential distribution of genus composition in swine and cattle (*P*-value < 0.01; median relative abundance in any sample > 1%).

The genus-level composition was significantly different between swine and cattle (*P*-value < 0.001, PERMANOVA test). NMDS also showed a distinct separation between swine and cattle (Fig. 2A). Because swine and cattle had significantly different compositions, samples from each group were clustered distinctively. In addition, the effect of the farm on the variation of bacterial taxonomic abundance in swine and cattle was significant (*P* < 0.001, PERMANOVA test). Notably, the α diversity in cattle was lower than that in swine (Supplementary Fig. S4). The Shannon diversity for swine was 1.86, while that for cattle was 1.23 on average. Similarly, the inverse Simpson index for swine was 4.91, while that for cattle was 2.96 on average.

### Pervasive antibiotic resistance genes in the gut microbiomes of swine and cattle

The abundance of resistomes was investigated by using reads per kilobase and million reads (RPKM) of resistance genes. According to the antibiotic resistance ontology provided by the Comprehensive Antibiotic Resistance Database (CARD) [18], the resistance genes identified from the gut microbiomes were assigned to the classes based on the determinant types. It should be noted that efflux pump–related genes were excluded in this study because the homology search of such genes was less accurate, as found in previous studies [19, 20]. In both swine and cattle, the median numbers of RPKM were >0 in 7 classes: aminoglycoside, β-lactam, lincosamide, nucleoside, macrolide, tetracycline, and macrolide-lincosamide-streptogramin shared (MLS). This finding implies that the prevalence of each of these 7 classes was >50%.

Notably, the abundance of the resistance genes was higher in swine than in cattle. This observation is consistent with the resistant phenotypes. In the antimicrobial susceptibility testing with *Escherichia coli*, frequently observed resistance in both swine and cattle was that against aminoglycosides, sulfonamides, and tetracyclines (Table 1). For cattle, the resistance was observed against only 4 classes of antibiotics: tetracycline, aminoglycoside, sulfonamide, and quinolone. Moreover, the resistance rates observed from the antimicrobial susceptibility test with *E. coli* were relatively lower in cattle than in swine (Table 1). In cattle, the resistance rates were 41.5% for tetracyclines, 28.2% for aminoglycosides, 28.2% for sulfonamides, and 12.8% for quinolones. In swine, the resistance rates were 66.7% for tetracyclines, 66.7% for aminoglycosides, 66.7% for sulfonamides, and 33.3% for quinolones. In addition, the resistance was observed against most of the antibiotic types in swine (Table 1). Overall, the prevalence of resistance and the MIC_50_ and MIC_90_ values in swine were higher than those in cattle.

**Table 1:** Antibiotic resistance of *Escherichia coli* (*n* = 77) isolated from animal fecal samples

Antimicrobial subclass	Antimicrobial agent	Breakpoint (µg/mL)	Swine (n = 36)			Cattle (n = 41)		
			MIC_50_ (µg/mL)	MIC_90_ (µg/mL)	Resistance % (No.)	MIC_50_ (µg/mL)	MIC_90_ (µg/mL)	Resistance % (No.)
Aminoglycosides	Gentamicin	≥16	1	32	27.8 (10)	1	1	0
	Streptomycin	≥32	64	128	66.7 (24)	16	64	26.8 (11)
Aminopenicillin	Ampicillin	≥32	64	64	69.4 (25)	4	4	0
β-lactam/-lactamase inhibitor combinations	Amoxicillin/clavulanic acid	≥32/16	8	8	0	2	4	0
Cephamycin	Cefoxitin	≥32	4	8	0	4	4	0
Cephalosporin III	Ceftiofur	≥8	0.5	0.5	2.8 (1)	0.5	0.5	0
	Ceftazidime	≥16	1	1	0	1	1	0
Cephalosporin IV	Cefepime	≥16	0.25	0.25	0	0.25	0.25	0
Carbapenem	Meropenem	≥4	0.25	0.25	0	0.25	0.25	0
Fluoroquinolone	Ciprofloxacin	≥4	0.25	8	16.7 (6)	0.12	0.25	0
Folate pathway inhibitors	Trimethoprim/Sulfamethoxazole	≥4/76	0.25	4	33.3 (12)	0.12	0.12	0
Sulfonamides	Sulfisoxazole	≥512	512	512	66.7 (24)	32	512	26.8 (11)
Phenicols	Chloramphenicol	≥32	64	64	66.7 (24)	8	8	0
Polymyxins	Colistin	≥4	2	2	0	2	2	0
Quinolone	Nalidixic acid	≥32	8	128	33.3 (12)	2	64	12.2 (5)
Tetracyclines	Tetracycline	≥16	64	128	66.7 (24)	2	128	41.5 (17)

MIC_50_ and MIC_90_ are the concentrations at which 50% and 90% of the isolates, respectively, were inhibited.

**Figure 3:**
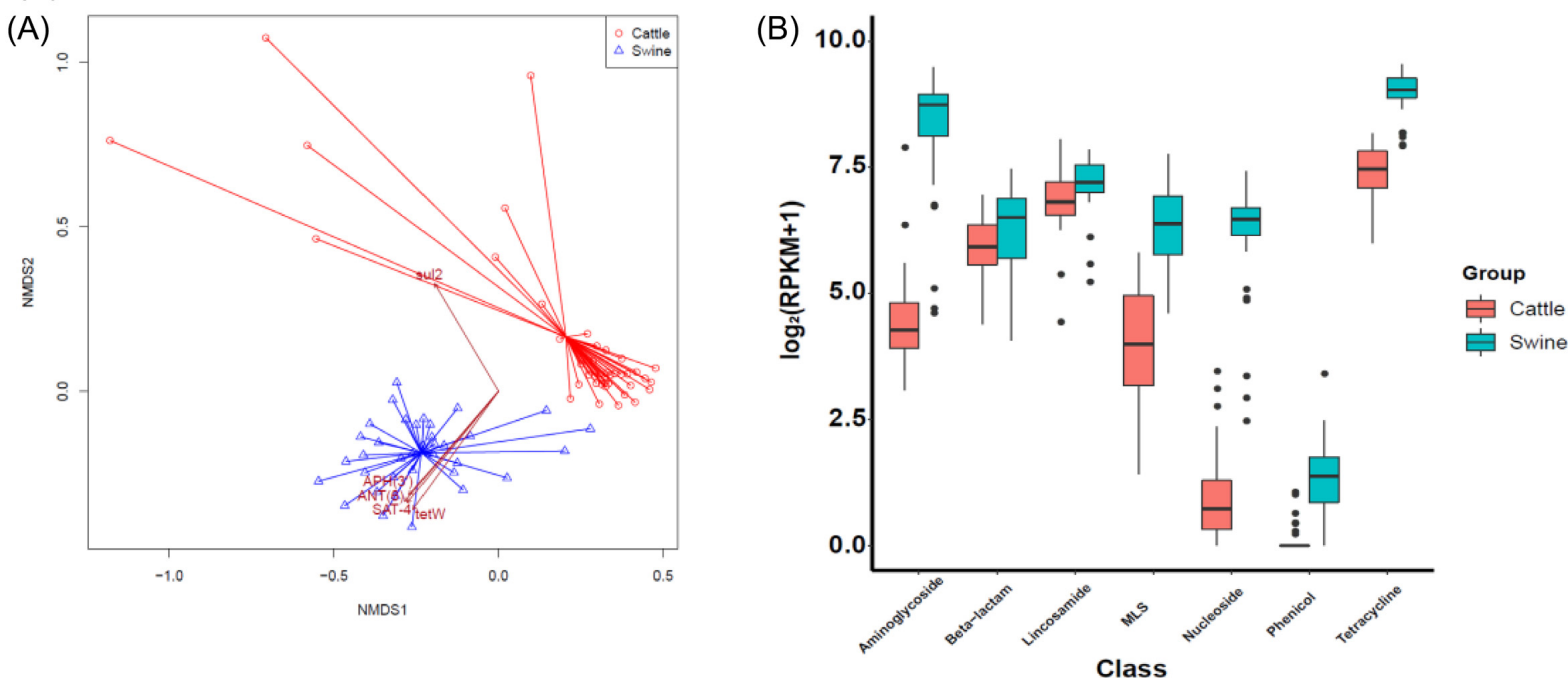
Composition of antibiotic resistance genes in swine and cattle. (A) Nonmetric multidimensional scaling (NMDS) analysis with the abundance of antibiotic resistance genes in swine and cattle. (B) Distribution of significant antibiotic resistance determinants in swine and cattle (*P*-value < 0.01).

Notably, our non-metric multidimensional scaling (NMDS) analysis showed that the samples from each animal were clustered distinctively (Fig. 3A). Statistical analysis showed that the distribution of resistance genes was significantly different between swine and cattle (*P*-value < 0.001, PERMANOVA test). The major factors that showed differential abundance were aminoglycoside, tetracycline, lincosamide, β-lactam, nucleoside, phenicol, and MLS. The abundance of aminoglycoside resistance genes in swine was higher than that in cattle: 427.22 vs 18.40 median RPKM in swine and cattle, respectively (Fig. 3). Consistently, sales of aminoglycoside antibiotics intended for swine have been higher than those intended for cattle (Fig. S3). In addition, β-lactam, lincosamide, MLS, and phenicol resistance genes were more enhanced in swine: 90.29 vs 60.06 RPKM in swine and cattle for β-lactam; 146.27 vs 111.55 for lincosamide; 82.81 vs 14.96 for MLS; 1.60 vs 0 for phenicol. Notably, the effect of the farm was significant in the variation of resistance gene abundance in swine and cattle (*P* < 0.001, PERMANOVA test).

In a previous study on cows in the United States that were fed with corn, the resistance genes of aminoglycoside, MLS, and tetracycline were mainly detected [11]. Zaheer et al. also observed that tetracycline resistance genes were the most abundant class in cattle [21]. In the analysis of ARG abundance in cattle and feedlot environment, Noyes et al. found that tetracycline resistance genes were the most abundant gene families, followed by MLS and aminoglycoside [22]. Wang et al. screened the ARGs in the swine microbiomes and observed that aminoglycoside and tetracycline resistance were the dominant resistance determinants, followed by β-lactam resistance [23]. The major resistance determinants, such as aminoglycoside and β-lactam, were also dominant in the human microbiomes, because streptomycin and penicillin were the most prevalent antibiotics that were administered for both humans and animals [24, 25].

### Resistance gene families differentially enhanced in the gut microbiomes of swine and cattle

**Figure 4:**
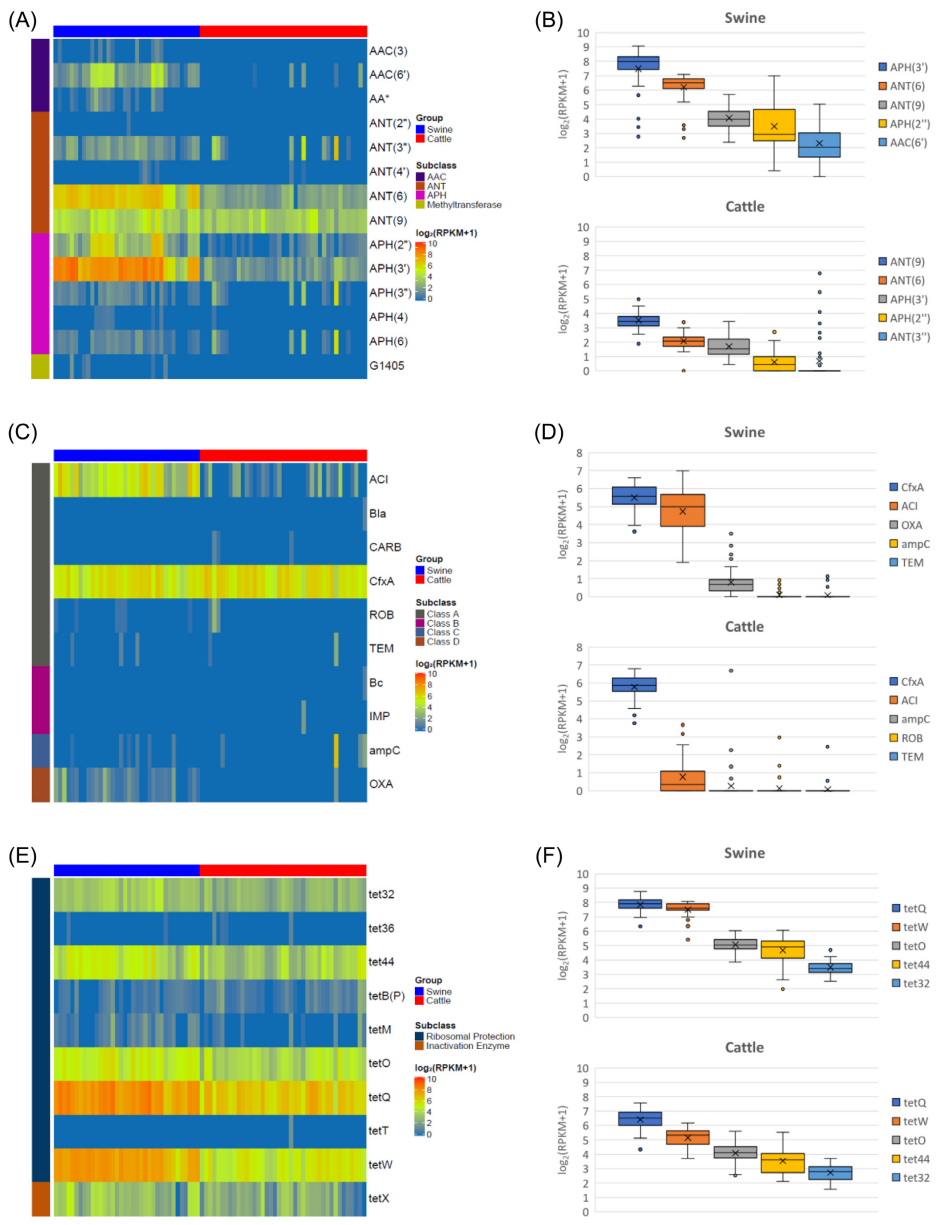
Antibiotic resistance genes in swine and cattle gut microbiomes. Binary heat map showing the presence of resistance genes for (A) aminoglycosides, (C) β-lactams, and (E) tetracyclines. The 5 most abundant gene families of antibiotic resistance genes for (B) aminoglycosides, (D) β-lactams, and (F) tetracyclines. The y-axis represents log-transformed RPKM. Boxplots display the median as the middle line and x as the mean whilst the perimeters of the box display the 1st and 3rd quantiles of the data.The whiskers span the range of the 25% quantile or the 75% quantile plus 1.5 times the interquartile range, and dots are outliers.

Although aminoglycoside was one of the most abundant resistance determinants in both swine and cattle, major constituents of this class of antibiotics are related to different resistance gene families. Among aminoglycoside acetyltransferase (AAC), aminoglycoside phosphotransferase (APH), and aminoglycoside nucleotidyltransferase (ANT), AAC resistance genes were rarely found in cattle (Fig. 4A). Moreover, there were predominant gene families of ANT and APH in swine and cattle: *ANT(6)* and *APH(3′)*. The prevalences of *ANT(6), ANT(9)*, and *APH(3′)* were 100%, 100%, and 100% in swine and 97.56%, 100%, and 100% in cattle samples, respectively. Even though *APH(3′)* and *ANT(6)* are the most abundant gene families in cattle, the overall abundance of ARGs in swine is evidently higher than that in cattle: 249.57 vs 1.91 RPKM for *APH(3′)* in swine and cattle; 90.47 vs 3.21 RPKM for *ANT(6)*; 14.97 vs 9.96 RPKM for *ANT(9)*. Most of the ANT, APH, and AAC genes other than *ANT(6), ANT(9)*, and *APH(3′)* showed low prevalence in cattle. Notably, *AAC(6′), APH(2′′), APH(3′′), APH(6)*, and *ANT(3′′)* were highly prevalent in swine but not in cattle: 97.22%, 100%, 77.78%, 83.33%, and 94.44% of prevalence for swine; 12.20%, 60.98%, 26.83%, 24.39%, and 24.93% for cattle, respectively.

β-Lactam resistance gene families were not diverse in swine and cattle, compared to aminoglycoside resistance gene families (Fig. 4C and D). In both swine and cattle, the most abundant β-lactam gene family was *CfxA*, which existed mostly in *Bacteroides* and *Prevotella. CfxA* was the most abundant in swine, followed by *ACI* and *OXA. ACI* and *OXA* were more prevalent in swine than in cattle. The prevalence of *ACI* was 100% and 53.66%, and that of *OXA* was 80.56% and 2.44%, in swine and cattle, respectively. Most of the *OXA* genes in our samples were *OXA-2, OXA-61*, and *OXA-335*. Fortunately, extended-spectrum β-lactamase resistance genes such as *SHV, TEM*, and *CTX-M* were rarely found in this study.

Tetracycline was highly prevalent in cattle and swine (Fig. 4E). Interestingly, the 5 most abundant gene families were common in cattle and swine: *tet32, tet44, tetO, tetQ*, and *tetW*. All of the swine and cattle samples contained these resistance genes. The prevalence of these genes was 100%. These 5 genes were also found as the dominant tetracycline resistance genes in cattle by Zaheer et al. [21]. *Tet32* was one of the most prevalent families in swine; it was also observed in a previous study [26]. *Tet32, tetO, tetQ*, and *tetW* were identified as the prevalent genes in humans in China, Denmark, and Spain [3, 4]. By means of PCR-based screening, Bryan et al. screened *E. coli* isolates from swine and found higher tetracycline resistance than those from cattle after the analysis of 14 tetracycline resistance genes [27]. In particular, *tetA* and *tetB* were found as dominant genes in swine and cattle.

### Homologous resistance genes found across different farm animals

**Figure 5:**
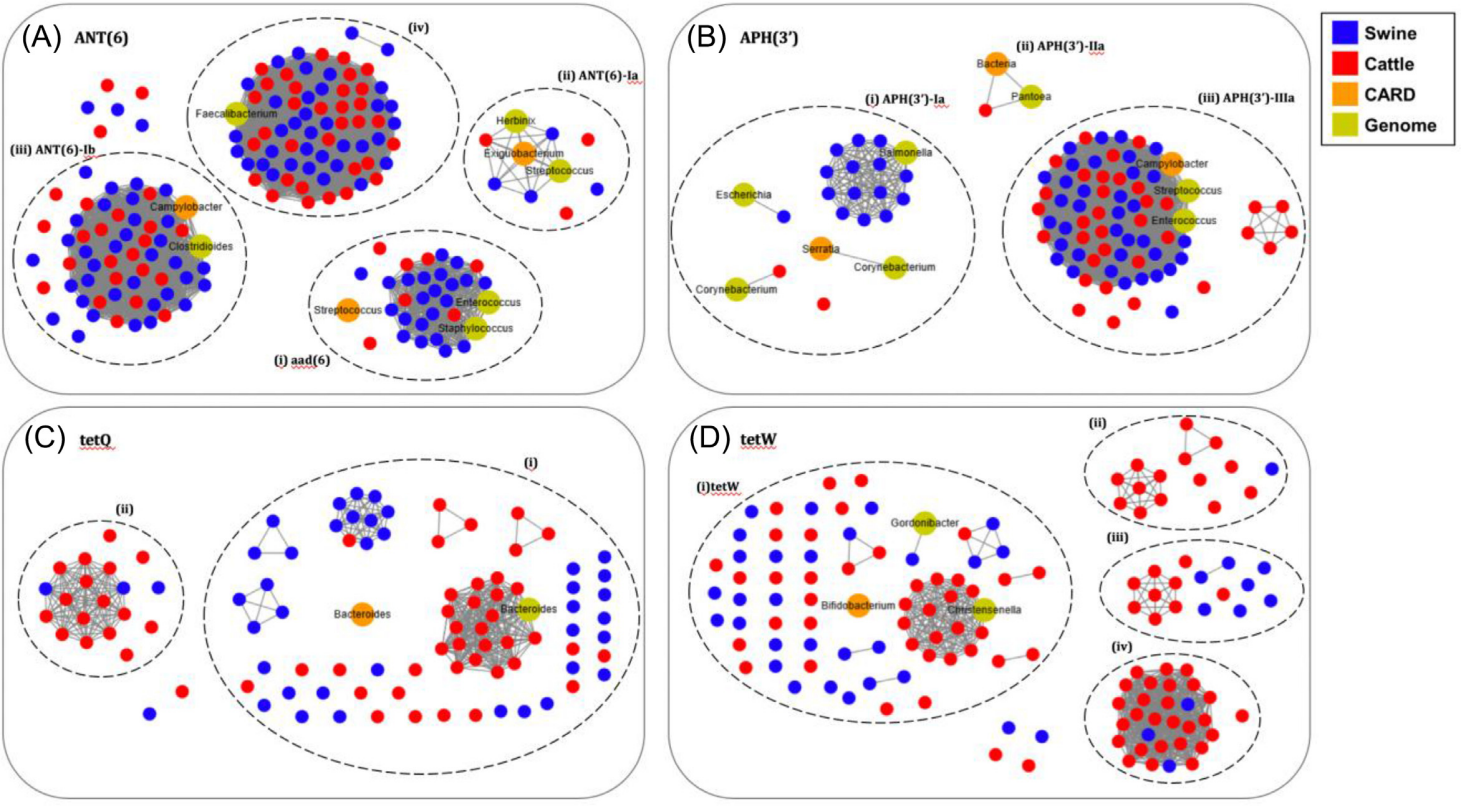
Network analysis of resistance genes and their similarity in swine and cattle gut microbiomes. Network of (A) *ANT(6)*, (B) *APH(3′)*, (C) *tetQ*, and (D) *tetW*. The nodes in the network are resistance genes identified in swine (blue) and cattle (red), stored in the CARD database (orange) and identified from the bacterial complete genomes (yellow). The solid lines connecting nodes represent 100% similarity between 2 ARG sequences. The same ARG sequences from the samples were connected as a cluster. The clusters in each dotted circle show ≥98% similarity.

*ANT(6)* was the most prevalent gene in both swine and cattle; it was found in all samples, except 1 sample of cattle (Fig. 4A). For the network analysis on the prevalent aminoglycoside resistance genes, *ANT(6)* genes were also identified from the bacterial genomes in the NCBI repository to reveal the bacterial source of ARGs in our samples. The *ANT(6)* genes found in the samples were homologous with 3 known genes: *ANT(6)-Ia* of *Exiguobacterium, ANT(6)-Ib* of *Campylobacter*, and *aad(6)* of *Streptococcus* (Fig. 5A; in orange color). In particular, a large amount of the resistance genes assembled from the samples were mainly associated with *ANT(6)-Ib* and *aad(6)*. In the graphs shown in Fig. 5, the genes that share 100% sequence similarity were connected as a cluster and the clusters that share ≥98% sequence similarity were grouped in a dotted circle. For the *ANT(6)* gene family, *ANT(6)-Ib* genes are common in both swine and cattle (Fig. 5A). The *ANT(6)-Ib* genes were also clustered together with the genes in *Clostridioides difficile* and *Campylobacter fetus* (Fig. 5A). Interestingly, there was a cluster (iv) (see Fig. 5A) of genes that shared ∼73% similarity to the protein sequence of *ANT(6)-Ib*. This cluster might be a gene family that is prevalent in Korean swine and cattle. In our study, this particular gene was found in all cattle samples. Notably, the *aad(6)* gene was also found in all the cattle samples; it mostly originates from *Staphylococcus* and *Enterococcus* species.

*APH(3′)* was also prevalent in both swine and cattle (Fig. 5B). Two genes, *APH(3′)-Ia* and *APH(3′)-IIIa*, were enhanced in our samples. Most of the *APH(3′)-Ia* genes were found in swine; *APH(3′)-IIIa* was found in both swine and cattle, but its abundance was much higher in swine (Fig. 5B). The *APH(3′)-Ia* genes were found in *Salmonella, Escherichia, Corynebacterium*, and *Serratia* species. The 14 *APH(3′)-Ia* genes in the swine were the same as those in *Salmonella*. The *APH(3′)-IIIa* genes in swine and cattle were identical to those in *Enterococcus* and *Streptococcus* species.

For tetracycline, there were 2 most abundant and prevalent genes: *tetQ* and *tetW* (Fig. 5C and D). In the search with the *tetQ* gene, 2 homologous clusters were built. The ARGs in the bigger cluster (Fig. 5C(i)) showed 100% sequence similarity with the genes in *Bacteroides* and *Prevotella*. The *tetW* gene family consisted of more diverse gene clusters. The biggest cluster that contains genes from swine and cattle was similar to the annotated genes of *Bifidobacterium* in the CARD database (Fig. 5D(i)). The other clusters showed lower sequence similarity against the known genes, which was ∼93% for cluster (ii), 88% for cluster (iii), and 78% for cluster (iv). For more accurate annotation, they need to be validated by antibiotic susceptibility testing.

## Discussion

Farm animals such as swine and cattle are usually treated with antibiotics to prevent infectious diseases and to promote their growth [5]. Moreover, manure or wastewater from animal farms contains more abundant resistance genes than other environments such as soil and rivers [26]. A large-scale study on the prevalence and diversity of antibiotic resistance genes in farm animals should help better elucidate the current situation of antibiotic resistance prevalence, and accordingly guide the development of public health policies. In this work, we comprehensively investigated resistomes in farm animals such as swine and cattle using a set of unbiased shotgun sequencing data. From a total of 36 swine in 12 farms and 41 cattle in 13 farms, gut microbiomes were collected nationwide and sequenced to identify resistance genes.

Swine are administered more antibiotics than cattle because of their dense breeding environment and higher exposure rate to bacterial diseases. Antibiotic sales data in Korea showed ∼510 tons of antibiotics for swine each year in 2017 and 2018, whereas 88 tons for cattle (Supplementary Fig. S3). The sales rates for β-lactams, tetracyclines, aminoglycosides, sulfonamides, macrolides, and phenicols were particularly high, which is consistent with the abundance of resistance gene determinants that we identified in the swine and cattle gut microbiomes. In summary, our study has revealed a general association between antibiotic usage, resistance phenotype, and resistance genes in the host gut microbiomes (Supplementary Fig. S5). Overall, antibiotic sales, resistance rates from the susceptibility tests, and the abundance of resistance genes in the gut microbiomes were higher in swine than in cattle. Aminoglycoside, β-lactam, tetracycline, and MLS are the abundant resistance classes in terms of antibiotic sales, susceptibility testing, and genes accumulated in the gut microbiomes.

The sequence homology of resistance genes was investigated to determine the possibility of gene transfer between swine and cattle. Among the aminoglycoside resistance genes, 2 gene families, *APH(3′)* and *ANT(6)*, were prevalent in both swine and cattle. *APH(3′)*, however, showed strong conservation separately in swine and cattle. Specifically, the *APH(3′)-la* gene was dominant in swine, whereas the *APH(3′)-llla* gene was in both cattle and swine. On the other hand, *ANT(6)* showed different patterns. The *ANT(6)-lb* gene, identified originally in *Campylobacter*, was found in both swine and cattle.

The most prevalent and abundant tetracycline genes that we found in this study were *tetQ, tetQ, tet32, tet44*, and *tetW*. In a previous study [26]*tet32* was one of the most prevalent families in swine. *Tet32, tetO, tetQ*, and *tetW* were identified as the most prevalent genes in humans in China, Denmark, and Spain [3, 4]. These genes were also observed abundantly in the manure, but not in regular soil [5]. This observation might suggest that they are human- and animal-related resistance genes.

In Korea, penicillins, tetracyclines, and aminoglycosides are the 3 most frequently used antibiotics for cattle. For swine, penicillins, phenicols, and tetracyclines are the 3 most highly used antibiotics. While the abundant resistance determinants found in our study were related to these antibiotics, the association with the amount of use of these antibiotics was not strong. For example, phenicol resistance genes were not abundant in our samples, although phenicol is one of the most frequently administered antibiotics. A similar discrepancy was also observed in a previous study [5]. However, the observation that phenicol resistance genes were observed in swine, but not in cattle, is consistent with the antibiotic usage trend in Korea.

## Potential Implications

From the gut microbiomes of 36 swine and 41 cattle, large-scale metagenomic analysis was performed to find the prevalence and diversity of ARGs in 2 different types of farm animals in Korea. This genome-level investigation of ARGs in multiple farm animals should provide valuable information to better elucidate horizontal and vertical transfer of ARGs in farm animals. In particular, the investigation of tetracycline ARGs identified in the microbiomes showed that identical *tetQ* and *tetX* genes were found in both swine and cattle, while several types of ARG sequences were quite different between the 2 animals. This observation establishes the presence of 2 different patterns of resistance genes: ' type is host-dedicated, and the other is prevalent in different hosts. An in-depth study of resistomes should also help analyze how antibiotic resistance genes spread among livestock, environments, and human microbiomes.

## Methods

### Sample collection

A total of 41 fecal samples of cattle were collected from 13 farms located in 6 provinces. For each farm, 3 or 4 samples were collected from different animals to compare the diversity depending on the farming environment. Of the 13 farms, 3 had <50 head, 2 had 50–100 head, and 8 had >100 head. The age of cattle ranged from 19 to 90 months (mean 34 months). In each farm, 5 cattle were randomly chosen. From these 5 samples, 3 samples with different antibiotic resistance patterns of *E. coli* were selected (Supplementary Table S2).

A total of 36 fecal samples were collected from 12 swine farms located in 6 provinces. For each farm, 3 samples were collected from different animals to compare the diversity depending on the farming environment. Of the 12 farms, 1 farm had <1,000 head, 9 had 1,000–5,000 head, 1 had 5,001–10,000 head, and 1 had >10,000 head. The age of the swine ranged from 150 to 230 days. In each farm, 5 swine were randomly chosen. From these 5 samples, 3 samples with different antibiotic resistance patterns of *E. coli* isolated were selected (Supplementary Table S3).

### DNA preparation

The samples were immediately transported to the laboratory in ice-cooled containers and stored at –70˚C until DNA extraction was performed. Each sample was thoroughly mixed using a spatula and divided into 250–300 mg aliquots. The total DNA was extracted using the Fast DNA SPIN Kit for Feces (MP Biomedicals, #116,570,200) following the manufacturer's instructions. DNA purity and concentration were evaluated by measuring the absorbances (ABS) at 260 and 280 nm using a NanoDropTM spectrophotomer (NanoDropTM 2000, Thermo Fisher Scientific Inc., Wilmington, DE, USA). All the DNA samples had ABS_260_/ABS_280_ ratios of 1.8–2.0. Illumina HiSeq4000 Platform (Illumina, San Diego, CA, USA) was used to sequence the DNA samples. We used the TruSeq DNA PCR Free Kit (Illumina, San Diego, CA, USA) and did not include a PCR amplification step.

### Sequencing and read filtering

A total of 77 gut microbiomes were sequenced from swine and cattle for this study. For every sample, 151-bp paired-end sequences were generated from the insert of 350 bps. An average of 38 M pairs of reads (ranging between 25 M and 75 M) were generated from each sample after filtering. Low-quality reads were removed using Sickle [28] with the threshold of Phred quality score >20 and read length >90 bp (pe -q 20 -t sanger -l 90). Reads containing “N” were also removed. Finally, host contamination was removed by discarding the reads that were mapped to the swine and cattle genomes provided by NCBI. For this process, bowtie2-align version 2.1.0 was used with the sensitive-local option. The swine reference genome used was Swine –Sscrofa11.1 (GCF_0 00003025.6). The cattle reference genome used was Cattle—Bos_taurus_UMD_3.1.1 (GCF_0 00003055.6).

### Antimicrobial susceptibility testing

Samples were processed, and *E. coli* was isolated as described previously [29] using eosin methylene blue agar (BD, Sparks, MD, USA) and MacConkey agar plates (BD). Species identification was performed using matrix-assisted laser desorption ionization time-of-flight mass spectrometry (bioMérieux, Marcy l'Étoile, France).

Antimicrobial susceptibility was assessed by determining the minimum inhibitory concentrations (MICs) for 16 antimicrobial agents using the broth microdilution method with a commercially available Sensititre® panel KRVP4F (TREK Diagnostic Systems, West Sussex, UK) according to the manufacturer's instructions. The following antibiotics were tested: ampicillin, amoxicillin/clavulanic acid, cefoxitin, ceftiofur, ceftazidime, cefepime, chloramphenicol, ciprofloxacin, colistin, gentamicin, meropenem, nalidixic acid, streptomycin, sulfisoxazole, tetracycline, and trimethoprim/sulfamethoxazole. The reference strain *E. coli* ATCC 25922 was used as quality control when determining MICs. The MIC was interpreted according to the Clinical and Laboratory Standards Institute (CLSI) guidelines (CLSI, 2017). When CLSI breakpoints were not available, the MIC was interpreted according to the Danish Integrated Antimicrobial Resistance Monitoring and Research Programme (DANMAP, 2014). Multidrug resistance was defined as resistance to ≥3 antibiotic subclasses.

### Profiling and statistical analysis of bacterial composition

After the filtering of sequencing reads, bacterial composition was profiled using MetaPhlAn [30] with the default option. For homology search in the MetaPhlAn process, bowtie2 with the very-sensitive option was used. To compare the bacterial composition of swine and cattle, α and β diversity was measured [31, 32] and plotted using the vegan package (ver. 2.5–6). For α diversity, the Shannon index and Simpson index were calculated. From each sample, 25 M reads were sampled and its taxonomy composition was determined by using MetaPhlAn. The genus proportion was used for calculating the Shannon index and Simpson index. Two different thresholds were applied to include genera in the calculation: (i) >0.1% in any of the samples and (ii) without any proportion threshold. For β diversity, NMDS was performed with Bray-Curtis distance. The metaMDS() function in the vegan package was used. The differential abundance of bacterial taxon between swine and cattle was determined using the *t*-test (scipy.stats.ttest_ind() in scipy package). To estimate the correlation between bacterial genera, SparCC was performed on the composition data. To determine the effects of feedlots on bacterial composition, PERMANOVA was performed using the adonis function of the vegan package.

### Profiling the abundance of antibiotic resistance genes

The abundance of ARGs was measured by RPKM, as performed in a previous study [33]. CARD version 2.0.1 was used to create a file consisting of 848 representative ARG sequences after clustering homologous sequences using cd-hit (-c 0.9 -n 8). Minor changes were added to the database file. *AAC(6′)-Ib’* and *OXA-368* sequences from CARD v.3.0.7 were used to update the database file. *BLA1* from class A was excluded because it was clustered with *BcI* from class B, and genes clustered with the *ANT(3′′)-li-AAC(6′)-IId* fusion protein were all excluded and handled separately in the post-processing step. Sample reads were aligned to the representative sequences using bowtie2 (–sensitive-local). The reads aligned were retained if the aligned length was longer than 50% of the ARGs, and their similarity was >70%. By using these quality-controlled reads, RPKM was calculated as follows:

$$\begin{equation*}
{\rm{RPKM\ }} = \ \frac{{{\rm{Number\ of\ reads\ mapped\ to\ reference\ }} \times {\rm{\ }}{{10}^9}}}{{{\rm{Number\ of\ reads\ in\ sample\ }} \times {\rm{\ Reference\ length}}}}.
\end{equation*}$$

Finally, the genes were considered to exist when 70% of the ARG length was covered by the reads.

### Identification of complete antibiotic resistance genes and network analysis

To identify complete antibiotic resistance genes that contain start and stop codons, a 3-step procedure was performed. First, filtered reads were assembled into contigs using MEGAHIT [34] with default options using only paired-end reads. Only contigs of length >500 bp were used for gene prediction. In addition, we further expanded the assembly with the reads collected with 3 different conditions to improve the assembly: (i) all reads that were mapped to the sequences in the ARG database, (ii) a subset with 5% of reads randomly selected, and (iii) reads mapped to the genes of high depth (>100). Because different approaches might generate redundant genes in a sample, clustering was applied using cd-hit-est (-c 1 -n 8) on the predicted genes to create a set of non-redundant ARGs in each sample. Second, to predict genes from contigs, FragGeneScan [35] was applied with the options of no sequencing errors (-w 1 –t complete). Last, genes predicted in the metagenomic data set were aligned with the antibiotic resistance genes annotated in CARD [18]. CARD version 2.0.1 includes a total of 2,252 protein sequences. Antibiotic resistance genes from uncultured bacteria and the genes annotated as regulatory system or efflux pump related were excluded. The resistance genes were classified into 21 ARG classes based on the gene ontology [18]. We added MLS classes that have *Cfr 23S* and *Erm 23S* as subclasses because these 2 subclasses are commonly found in the 3 classes. Blastp [36] was used for ARG annotation with an e-value threshold of 1 × 10^–10^, similarity >70%, and reference coverage >70%.

With the ARGs identified from the samples, network analysis was performed with the annotated genes. To collect the annotated genes, all the genes of the 8,369 complete genomes downloaded from the NCBI repository [37] were searched against the ARG database. A network graph was built with the nodes of ARGs using cytoscape [38]. Colors of the nodes represent either the sample or host genus. The nodes were connected with a full line if 2 ARG sequences were the same (similarity of 100% with 100% coverage). The clusters were further grouped within a dotted circle if they shared ≥98% similarity.

## Availability of Supporting Data and Materials

Sequencing datais available via the EBI project ID PRJEB32496. Other data further supporting this work are openly available in the *GigaScience* GigaDB repository [39].

## Additional Files

Table S1. Sequencing data information for swine and cattle gut microbiomes

Table S2. Sample information for cattle

Table S3. Sample information for swine

Figure S1. Correlation on bacterial composition

Figure S2. Bacterial composition at family and species-level in swine and cattle microbiomes

Figure S3. Estimated amount of antibiotics sold per year in swine and cattle during 2017-2018

Figure S4. Alpha diversity of bacterial composition in swine and cattle

## Abbreviations

AAC: aminoglycoside acetyltransferase; ANT: aminoglycoside nucleotidyltransferase; APH: aminoglycoside phosphotransferase; ARG: antibiotic resistance gene; BD: Becton Dickinson; bp: base pairs; CARD: Comprehensive Antibiotic Resistance Database; CLSI: Clinical and Laboratory Standards Institute; MIC: minimum inhibitory concentration; MLS: macrolide-lincosamide-streptogramin shared; NCBI: National Center for Biotechnology Information; NMDS: nonmetric multidimensional scaling; PERMANOVA: permutational multivariate analysis of variance; RPKM: reads per kilobase and million reads; SparCC: sparse correlations for compositional data.

## Competing Interests

The authors declare that they have no competing interests.

## Funding

This research was supported by a grant (2017NER54070) from Research of Korea Centers for Disease Control and Preventionto S.L. and M.R.

## Authors' Contributions

S.L. and M.R. conceived and designed the study. S.L. performed the experiments and analysis. D.K., Y.C., and M.R. performed the analysis. D.M. prepared the samples and performed experiments. All authors wrote the manuscript. All authors read and approved the final manuscript.

## Supplementary Material

giaa043_GIGA-D-19-00340_Original_Submission

giaa043_GIGA-D-19-00340_Revision_1

giaa043_GIGA-D-19-00340_Revision_2

giaa043_Response_to_Reviewer_Comments_Original_Submission

giaa043_Response_to_Reviewer_Comments_Revision_1

giaa043_Reviewer_1_Report_Original_Submission Arnaud Bridier -- 11/22/2019 Reviewed

giaa043_Reviewer_2_Report_Original_Submission Patrick Munk, Ph.D. -- 11/22/2019 Reviewed

giaa043_Reviewer_2_Report_Revision_1 Patrick Munk, Ph.D. -- 3/12/2020 Reviewed

giaa043_Supplemental_Figures_and_Tables
