# Supplementary material for: Antibiotic resistomes discovered in the gut microbiomes of Korean swine and cattle
Source: Gigascience. 2020 May 5;9(5):giaa043. doi: 10.1093/gigascience/giaa043 (PMC7317084; doi:10.1093/gigascience/giaa043)
Supplement: giaa043_GIGA-D-19-00340_Original_Submission [file giaa043_giga-d-19-00340_original_submission.pdf]

GigaScience

Antibiotic resistomes discovered in the gut microbiomes of swine and cattle

--Manuscript Draft--

**Manuscript Number:**	GIGA-D-19-00340
**Full Title:**	Antibiotic resistomes discovered in the gut microbiomes of swine and cattle
**Article Type:**	Research
**Funding Information:**	Korea Centers for Disease Control and Prevention (2017NER54070) — Dr. Suk-Kyung Lim<br>Dr Mina Rho
**Abstract:**	ABSTRACT<br>Background: Antibiotics administered to the farm animals have continuously been increased to accumulate in the microbiome and environments. While antibiotics treatments help cure infectious diseases of the farm animals, the possibility of spreading antibiotic resistance genes into the environment and human microbiome raises significant concerns. Through long-term evolution, antibiotic resistance genes are mutated, making the resistance problems even more complicated.<br>Results: In this study, we have performed deep sequencing on the gut microbiomes of 36 swine and 41 cattle in the farms, and metagenomic analysis to understand the diversity and prevalence of antibiotic resistance genes. We found that aminoglycoside, beta-lactam, lincosamide, streptogramin, and tetracycline are the prevalent resistance determinants in both swine and cattle. Aminoglycoside resistance is the most abundant in both swine and cattle. While aminoglycoside phosphotransferase family is significantly more enhanced in swine, aminoglycoside nucleotidyltransferase family is more abundant in cattle. Notably, phenicol is abundant in the swine, but not in the cattle, which correlates with the recent antibiotic usage for the farm animals in Korea. Interestingly, tetracycline genes are found in higher rates in cattle than in swine, with the three most prevalent tetracycline resistance genes of tetM, tetQ, and tetX.<br>Conclusions: Overall, antibiotic resistome is more diverse and pervasive in swine than in cattle. Genomic investigation of specific resistance genes from the gut microbiome of swine and cattle in this study should provide opportunities to better understand the exchange of antibiotic resistance genes in the farm animals.
**Corresponding Author:**	Mina Rho<br><br>KOREA, REPUBLIC OF
**Corresponding Author Secondary Information:**	
**Corresponding Author's Institution:**	
**Corresponding Author's Secondary Institution:**	
**First Author:**	Suk-Kyung Lim
**First Author Secondary Information:**	
**Order of Authors:**	Suk-Kyung Lim
	Dongjun Kim
	Dong-Chan Moon
	Youna Cho
	Mina Rho
**Order of Authors Secondary Information:**	
**Additional Information:**	
**Question**	**Response**
Are you submitting this manuscript to a	No

Powered by Editorial Manager® and ProduXion Manager® from Aries Systems Corporation

special series or article collection?	
**Experimental design and statistics**<br><br>Full details of the experimental design and statistical methods used should be given in the Methods section, as detailed in our Minimum Standards Reporting Checklist. Information essential to interpreting the data presented should be made available in the figure legends.<br><br>Have you included all the information requested in your manuscript?	Yes
**Resources**<br><br>A description of all resources used, including antibodies, cell lines, animals and software tools, with enough information to allow them to be uniquely identified, should be included in the Methods section. Authors are strongly encouraged to cite Research Resource Identifiers (RRIDs) for antibodies, model organisms and tools, where possible.<br><br>Have you included the information requested as detailed in our Minimum Standards Reporting Checklist?	Yes
**Availability of data and materials**<br><br>All datasets and code on which the conclusions of the paper rely must be either included in your submission or deposited in publicly available repositories (where available and ethically appropriate), referencing such data using a unique identifier in the references and in the "Availability of Data and Materials" section of your manuscript.<br><br>Have you have met the above requirement as detailed in our Minimum Standards Reporting Checklist?	Yes

Powered by Editorial Manager® and ProduXion Manager® from Aries Systems Corporation

Powered by Editorial Manager® and ProduXion Manager® from Aries Systems Corporation

Manuscript

Click here to download Manuscript AR_Farm_Animals_1001._new.docx

Antibiotic resistomes discovered in the gut microbiomes of swine and cattle

Suk-Kyung Lim[1], Dongjun Kim[2], Dong-Chan Moon[1], Youna Cho[2], Mina Rho[2,3]

[1]Bacterial Disease Division, Animal and Plant Quarantine Agency, Gimcheon, Korea
[2]Department of Computer Science and Engineering, Hanyang University, Seoul, Korea
[3]Department of Biomedical Informatics, Hanyang University, Seoul, Korea

To whom correspondence should be addressed: Mina Rho (Tel: 82-2-2220-2379; Email: minarho@hanyang.ac.kr).

ABSTRACT

Background: Antibiotics administered to the farm animals have continuously been increased to accumulate in the microbiome and environments. While antibiotics treatments help cure infectious diseases of the farm animals, the possibility of spreading antibiotic resistance genes into the environment and human microbiome raises significant concerns. Through long-term evolution, antibiotic resistance genes are mutated, making the resistance problems even more complicated.

Results: In this study, we have performed deep sequencing on the gut microbiomes of 36 swine and 41 cattle in the farms, and metagenomic analysis to understand the diversity and prevalence of antibiotic resistance genes. We found that aminoglycoside, beta-lactam, lincosamide, streptogramin, and tetracycline are the prevalent resistance determinants in both swine and cattle. Aminoglycoside resistance is the most abundant in both swine and cattle. While aminoglycoside phosphotransferase family is significantly more enhanced in swine, aminoglycoside nucleotidyltransferase family is more abundant in cattle. Notably, phenicol is abundant in the swine, but not in the cattle, which correlates with the recent antibiotic usage for the farm animals in Korea. Interestingly, tetracycline genes are found in higher rates in cattle than in swine, with the three most prevalent tetracycline resistance genes of tetM, tetQ, and tetX.

Conclusions: Overall, antibiotic resistome is more diverse and pervasive in swine than in cattle. Genomic investigation of specific resistance genes from the gut microbiome of swine and cattle in this study should provide opportunities to better understand the exchange of antibiotic resistance genes in the farm animals.

Keywords: Swine gut microbiome, Cattle gut microbiome, Antibiotic resistome, Antibiotic resistance gene

BACKGROUND

Antibiotics have been widely used for curing infectious diseases. In the farm, antibiotics have also been used to treat and prevent diseases or to promote the growth of animals. An increasing administration of antibiotics expedites the development of resistance, and spreads the resistance genes in the farming environment and human population [1]. Moreover, gene transfer from the environment or food chain to human population makes the problems more complicated. In particular, antibiotic resistance genes are more frequently transferred from one bacteria to others in the gut microbiome [2].

With the advances in high throughput sequencing technology and metagenomic analysis, gut microbiome has been investigated to understand the prevalence of antibiotic resistance genes (ARGs) and the compositional changes in the microbiome after treatment. In recent years, ARGs have been extensively studied to understand their diversity and abundance in the human microbiome in terms of the races and ages [3, 4]. The most prevalent resistance determinant in human is tetracycline [4], which is also prevalent in the farm animals [5]. Since tetracycline is widely applied for infection control and growth promotion, several studies suggested a positive correlation between the amount of its usage and prevalence [6]. In human skin microbiome [7] and soil microbiome [8], divergent ARGs that show low sequence similarity against the known genes have also been identified by functional metagenomics, implying that resistance genes have evolved in diverse environments.

For the gut microbiome of farm animals, several studies explored the prevalence of antibiotic resistance genes [5, 9]. A recent study on the ARGs in Chinese, French, and Danish swine showed that the highly prevalent classes of ARGs are tetracycline, beta-lactam, macrolide, streptogramin, and bacitracin [10]. Notably, the profile of ARGs in Chinese swine was different from those in the other two populations, in terms of the composition and abundance. Tetracycline, aminoglycoside, and beta-lactam were also abundant classes in the farm environments for swine [5], which was consistent with the ARG profiles of farm animals. The effects of antibiotics, used as feed additives, on the changes of bacterial composition was discussed. In the cattle microbiome, it was found that tetracycline is the most abundant class, followed by aminoglycoside [11]. A previous study suggested that ARGs in the animal microbiome can be transferred and distributed to other environments [12].

In this work, we have performed metagenomic analysis on the gut microbiome of swine and cattle to investigate the diversity and prevalence of ARGs in different farm environments. An unbiased screening of microbial resistance genes was performed using the metagenomic shotgun sequencing data. To our knowledge, this is the first study that investigates ARGs in multiple types of farm animals raised in Korea. An interesting observation is the presence of two different patterns of resistance genes: one type is host-dedicated, and the other is prevalent in different host animals.

DATA DESCRIPTION

We have performed deep sequencing on the gut microbiomes of 36 swine and 41 cattle in the farms (Supplementary Table S1), and metagenomic analysis to understand the diversity and prevalence of ARGs. All raw sequencing data described in this study is available at European Nucleotide Archive (ENA) with the accession number PRJEB32496.

Fresh fecal samples from healthy finishing swine and adult Korean cattle were collected aseptically on 25 feedlots throughout Korea between August 2017 and June 2018 (Supplementary Table S2 and S3), following guidelines of the Animal Protection Act of Animal and Plant Quarantine Agency. Farm selection was based on two criteria: geographical distribution and farm size.

Illumina HiSeqX Platform (Illumina, San Diego, USA) was used to sequence the DNA samples. A total of 77 gut microbiome were sequenced from swine and cattle for this study. For every sample, 151-bp paired-end sequences were generated from the insert of 350 base pairs. An average of 160 M paired-reads (ranging between 110 M and 229 M) were generated for each sample after filtering.

ANALYSIS

Bacterial composition of the swine and cattle gut microbiomes

A total of 36 gut microbiome from swine and 41 from cattle were collected to investigate the bacterial composition of gut microbiome. For each farm, three or four samples were collected from different animals to compare the diversity depending on the farming environment. Consistent with the previous studies [13-16], the major phylum in swine and cattle were *Bacteroidetes* and *Firmicutes*, which were also commonly observed in human gut microbiome [17]. The proportions, however, were quite different in two different animals: 21.65% and 67.16 % for swine; 4.15% and 58.62% for cattle (Figure 1C and D). The ratio of *Bacteroides* to *Firmicutes* was much higher in swine than in cattle.

The genus-level compositions were significantly different between swine and cattle (Figure 1A and B). In the swine gut microbiome, the major genera were *Lactobacillus* (21.19% as a median proportion), *Prevotella* (20.89%), *Subdoligranulum* (7.75%), and *Selenomonas* (7.06%). *Prevotella* was the major genus in *Bacteroidetes*, whereas *Lactobacillus*, *Subdoligranulum*, and *Selenomonas* were the major genera in *Firmicutes*. The proportion of *Prevotella* showed a negative correlation with those of *Lactobacillus* and *Subdoligranulum* (Supplementary Figure S1). At the species level, *P. copri* was the most abundant species, which comprised 17.23% of the microbiome (Supplementary Figure

S2). This value was significantly higher than any other species, such as *L. amylovorus* (7.80%), *Subdoligranulum* species (7.75%), and *S. bovis* (7.06%).

In the cattle microbiome, the major genera were the *Peptostreptococcaeae* genus (32.56%) and *Butyrivibrio* (10.77%). Several genera including *Treponema* and *Bifidobacterium* were observed, but they constituted lower than 1% of the composition. *Peptostreptococcaeae* genus and *Butyrivibrio* were the major genus in *Firmicutes*. Overall, the genus-level composition was significantly different between swine and cattle (Figure 1E and F). The *Treponema* was the bacterial genus that commonly exists in swine and cattle gut microbiome. The median composition in swine was 3.46 %. Although the median in cattle was below 1%, 19 samples contained *Treponema* species, ranging from 0% to 99%. Several genera showed high correlations in cattle. *Acinetobacter* was positively correlated with *Arthrobacter* and *Enterococcus*. *Treponema* was negatively correlated with *Escherichia* (Supplementary Figure S1B).

Principle component analysis (PCA) also supported the distinct separation between swine and cattle (Figure 2). Since swine and cattle had significantly different compositions, samples from each group were clustered distinctively. Notably, inter-individual diversity was higher in the cattle microbiome, compared to the swine microbiome (Figure 1A, B, and 2A). The intra-individual diversity in cattle was also higher than that in swine. The average number of genera that constituted more than 1% of the composition in swine was 13, while that in cattle was 15.

Pervasive antibiotic resistance genes in the gut microbiome of swine and cattle

The abundance of resistome was investigated by using the counts of resistance genes, which were normalized by the million genes predicted in the microbiome (GPM). According to the antibiotic resistance ontology provided by CARD database [18], the resistance genes identified from the gut microbiome were assigned to the classes based on the determinant types. It should be noted that efflux pump-related genes were excluded in this study since the homology search of such genes were less accurate, as found in previous studies [19, 20]. In both swine and cattle, the median numbers of GPM were higher than zero in six classes: aminoglycoside, bata-lactam, lincosamide, streptogramin, tetracycline, and macrolide-lincosamide-streptogramin shared (MLS) (Figure S3A and B). It implies that these six classes exist in more than half of the population.

Notably, the abundance was higher in swine than in cattle. This observation is consistent with the resistance phenotypes. In the antimicrobial susceptibility testing with *E.coli*, the most frequently observed resistance in both swine and cattle was to aminoglycosides, sulfonamides, and tetracyclines (Table 1). For cattle, the resistance was observed against only four classes of antibiotics: tetracyclines, aminoglycosides, sulfonamides, and quinolone. Moreover, the resistance rates were relatively lower,

compares to those in the swine: 43.6% for tetracyclines, 28.2% for aminoglycosides, 28.2% for sulfonamides, and 12.8% for quinolone. Notably, the resistance rates for such antibiotic classes were high in swine: 66.7% for tetracyclines, 66.7% for aminoglycosides, 66.7% for sulfonamides, and 33.3% for quinolone. In addition, the resistance was observed against most of the antibiotic types in swine (Table 1). On the whole, the prevalence of resistance and the values of MIC_{50} and MIC_{90} in swine were much higher than those in cattle.

Notably, our PCA showed that the samples in each animal were clustered distinctively (Figure 3A). The major factors were aminoglycoside, tetracycline, lincosamide, and beta-lactam. Aminoglycoside was the antibiotic resistance determinant with the most abundant ARGs both in swine and cattle (Figure S3A and B). However, the proportion of aminoglycoside in swine was significantly higher than that in cattle (p-value < 0.01): 8.58 vs 3.28 GPM in swine and cattle, respectively (Figure 3B). In addition, beta-lactam, lincosamide, MLS, and phenicol were significantly more enhanced in swine (p-value < 0.01): 2.80 vs 0.88 GPM in swine and cattle for beta-lactam; 2.33 vs 1.35 for lincosamide; 2.25 vs 1.49 for MLS; 0.97 vs 0 for phenicol. On the other hand, tetracycline was more enhanced in cattle (Figure S3A and B).

In a previous study on the cows in U.S. that were fed with corn, the genes of aminoglycoside, MLS, and tetracycline were mainly detected [11]. The major determinants, such as aminoglycoside and beta-lactam, were also dominant in the human microbiome, since streptomycin and penicillin were the most prevalent antibiotics that were administered for both human and animal [21, 22].

Resistance gene families differentially enhanced in the gut microbiome of swine and cattle

Although aminoglycoside was the most abundant determinant in both swine and cattle, they contained different resistance gene families as major constituents. Among the three gene families of aminoglycoside acetyltransferase (AAC), aminoglycoside phosphotransferase (APH), and aminoglycoside nucleotidyltransferase (ANT), AAC resistance genes were rarely found in cattle (Figure 4A). Moreover, there were one predominant gene family each in ANT and APH of cattle: ANT(6) and APH(3’). The prevalence of ANT(6) and APH(3’) were 98% and 90% of the samples, whereas ANT(3″), APH(2″), and ANT(9) were found in less than half of the samples with the prevalence of 22%, 49%, and 29%, respectively. The prevalence of such genes in swine and cattle were comparable: 94% and 69% for ANT(6) and APH(3’) in swine, respectively. The abundance of such genes were also comparable: 0.89 vs 0.76 GPM for APH(3’) in swine and cattle, respectively; 0.96 vs 1.45 for ANT(6) in swine and cattle, respectively. Even though APH(3’) and ANT(6) are the most abundant gene families in cattle, the overall abundance of swine is evidently higher than that of cattle. In swine, APH family was significantly more abundant than ANT and AAC (Figure 4B). In

particular, APH(2'') was evidently abundant than other gene families in swine (1.98 GPM for median).

Beta-lactam resistance gene families were not diverse in swine and cattle, compared to aminoglycoside (Figure 4C and D). In both swine and cattle, the most abundant beta-lactam gene family was CfxA, which exists mostly in *Bacteroides* and *Prevotella*. The relative abundance of CfxA was higher in swine than in cattle. CfxA was the most abundant in swine, followed by OXA. In particular, OXA-2, OXA-61, and OXA-335 were found with 98–100 % homology in our study. Notably, OXA was significantly more abundant and prevalent in swine than in cow. In fact, 44% of swine has OXA genes in their gut microbiome, but only one sample of cattle has OXA-335 genes.

In contrast to the observations made for aminoglycoside, tetracycline was more prevalent in cattle than in swine (Figure 4E). Interestingly, the five most abundant gene families were common in cattle and swine: tet32, tetM, tetB(P), tetX, and tetQ. The median abundance of these five families were still close to zero in swine, which implies that the prevalence is less than 50%. Tet32 was the most prevalent family in swine, which was also observed in the previous study [23]. In that study, tetM and tetQ were also abundant in swine [23]. Notably, tetM, tetQ, and tetX were abundant in cattle (Figure 4F). TetO was one of the most abundant resistance genes in human gut microbiome [4], but it was not found in swine, and found in only two samples of cattle.

Homologous resistance genes found across different farm animals

ANT(6) was the most prevalent in both swine and cattle; it was found in all samples except for two samples in swine and only one sample in cattle. For the network analysis on the prevalent aminoglycoside resistance genes, ANT(6) genes were also identified from the bacterial genomes in the NCBI repository. The ANT(6) genes found in the samples were homologous with three known genes: ANT(6)-la of *Exiguobacterium*, ANT(6)-lb of *Campylobacter*, and aad(6) of *Streptococcus* (Figure 5A; in orange color). In particular, most of the resistance genes found in the samples were mainly associated with ANT(6)-lb. In the graphs shown in Figure 5A and B, the genes that share 100% sequence similarity were connected as a cluster. For ANT(6) gene family, ANT(6)-lb genes are common in swine and cow (Figure 5A). The ANT(6)-lb genes were also clustered together with the genes in *Clostridioides difficile* and *Campylobacter fetus* (Figure 5A). Interestingly, there was a Cluster I (see Figure 5A) of genes that were all from the cattle, which share 73% similarity to the protein sequence of ANT(6)-lb. This cluster might be a new gene family that is prevalent only in cattle. In our study, this particular gene was found in all cattle samples. Notably, aad(6) gene was also found in all the cattle samples, which mostly originates from *Streptococcus* and *Enterococcus* species.

APH(3′) was also prevalent in both swine and cattle (Figure 5B). Two genes were almost exclusively enhanced. Most of the APH(3′)-Ia genes were found in swine; APH(3′)-IIIa in cattle (Figure 5B). The APH(3′)-Ia and homologous genes were found in *Samonella*, *Escherichia*, *Corynebacterium*, and *Serratia* species. The twenty genes in the swine were the same as those in *Samonella*. The APH(3′)-IIIa genes in cattle were identical to those in *Enterococcus* and *Streptococcus* species.

For tetracycline, there were three prevalent genes: tetQ, tetX, and tetM (Figure 5C, D, and E). In the search with the tetQ gene, a set of 25 tetQ genes in swine and cattle were clustered with 100% sequence similarity, but showed only 88.77% similarity with those of *Bacteroides* and *Prevotella* as the closest tetQ genes (Figure 5C). The TetX genes in swine and cattle were clustered with those in *Bacteroides* as the largest cluster (Figure 5D). Notably, tetQ and tetX were observed in a limited number of genera, and constitute a major cluster of genes shared by swine and cattle. The tetM genes were found in more genera including *Clostridium* and *Streptococcus* (Figure 5E). The tetM genes in swine showed high homology of about 99% with that in *Mycoplasma hominis*. The tetM genes in cattle, however, were significantly different from those in swine, showing relatively low similarity of 70%. The putative tetM genes were remotely homologous to the known ARG genes. For more accurate annotation, they need to be validated with antibiotics susceptibility testing.

DISCUSSION

Farm animals such as swine and cattle are usually treated with antibiotics to prevent infectious diseases and to promote growth [5]. Moreover, manure or wastewater from animal farms contains more abundant resistance genes than other environments such as soil and river [23]. A large-scale study for the prevalence and diversity of antibiotic resistance genes in the farm animals should help better understand the current situation of antibiotic resistance prevalence and develop public health policy. In this work, we have performed a comprehensive investigation of resistomes in the farm animals such as swine and cattle with the unbiased shotgun sequencing data. From a total of 36 swine in 12 farms and 41 cattle in 13 farms, gut microbiomes were collected nationwide and sequenced to identify the resistance genes.

Due to the dense breeding environment and higher expose rate to bacterial diseases, swine is administered more antibiotics. According to the antibiotics sales in Korea, about 510 tons of antibiotics were sold for swine every year between 2017 and 2018, whereas 88 tons for cattle (Supplementary Figure S3). The sales rates for beta-lactams, tetracyclines, sulfonamides, macrolides, phenicols, and aminoglycosides were particularly high, which is consistent with the abundant resistance gene determinants that we identified in the swine and cattle gut microbiome. In sum, our

study has revealed a general correlation among the antibiotics usage, resistance phenotype, and the resistance genes in the host gut microbiome.

The sequence homology of resistance genes was investigated to determine the possibility of gene transfer between swine and cattle. In the aminoglycoside resistance genes, two gene families of APH(3′) and ANT(6) were prevalent in both swine and cattle. APH(3′), however, showed strong conservation separately in swine and cattle. Specifically, APH(3′)-la gene was dominantly abundant in swine, whereas APH(3′)-llla gene in cattle. On the other hand, ANT(6) showed different patterns. The ANT(6)-lb gene, identified originally in *Campylobacter*, was found in both swine and cattle. In addition, there was a separate cluster of ANT(6) genes in cattle, which are remotely homologous to ANT(6)-lb.

The most prevalent tetracycline genes that we found in this study were tetM, tetQ, and tetX. These genes were also observed abundantly in the manure, but not in regular soil [5]. This observation might suggest that they are animal-related resistance genes. Interestingly, tetracycline genes were found in higher rates in cattle than in swine (Figure 6), which is consistent with a previous study. While identical tetQ and tetX genes were found in swine and cattle, the tetM gene sequences were quite different between the two animals (Figure 6D). An interesting observation was the presence of two different patterns of resistance genes: one type is host-dedicated; the other is prevalent in different hosts.

In Korea, penicillins, tetracyclines, and aminoglycosides are the three most frequently administered antibiotics for cattle. For swine, penicillins, phenicols, and tetracyclines are the three highly used antibiotics. While the abundant resistance determinants found in our study were related with these antibiotics, the correlation with the amount of antibiotics usage was not strong. For example, phenicol resistance genes were not abundant in our samples, although it is one of the most frequently administered antibiotics. A similar discrepancy was also observed in previous studies [5]. However, the observation that phenicol resistance genes were observed in swine, but not in cattle, is consistent with the antibiotics usage in Korea.

POTENTIAL IMPLICATION

From the gut microbiomes of 36 swine and 41 cattle, large-scale metagenomic analysis was performed to find the prevalence and diversity of ARGs in two different types of farm animals in Korea. This genomic level investigation of ARGs in the multiple farm animals should provide valuable information to better understand horizontal and vertical transfer of ARGs in the farm animals. In particular, the investigation of tetracycline ARGs identified in the microbiomes showed that

identical tetQ and tetX genes were found both in swine and cattle, while the tetM gene sequences were quite different between the two animals. This observation establishes the presence of two different patterns of resistance genes: one type is host-dedicated, and the other is prevalent in different hosts. An in-depth study of resistome should also help analyze how antibiotic resistance genes spread among livestocks, environments, and human microbiomes.

METHODS

Sample Collection

A total of 41 fecal samples of cattle were collected from 13 farms located in 6 provinces. Of the 13 farms, three have < 50 heads, two have 50–100 heads, and eight have > 100 heads. The age of cattle ranges from 19 to 90 months (average 34 months). In each farm, five cattle were randomly chosen. From these five samples, three samples with different antibiotic resistance patterns of *E. coli* were selected (Supplementary Table S2).

A total of 36 fecal samples were collected from 12 swine farms located in 6 provinces. Of the 12 farms, one farm has < 1,000 heads, nine have 1,000–5,000 heads, one has 5000–10,000, and one has > 10,000 heads. The age of the swine ranges from 150 to 230 days. In each farm, five swine were randomly chosen. From these five samples, three samples with different antibiotic resistance patterns of *E. coli* isolated were selected (Supplementary Table S3).

DNA preparation

The samples were immediately transported to the laboratory in ice-cooled containers and stored at –70 ˚C until DNA extraction was performed. Each sample was thoroughly mixed using a spatula and divided into 250–300 mg aliquots. The total DNA was extracted using the Fast DNA SPIN Kit for Feces (MP Biomedicals, #116570200) following the manufacturer’s instructions. DNA purity and concentration were evaluated by measuring the absorbances (ABS) at 260 nm and 280 nm using a NanoDropTM spectrophotomer (NanoDropTM 2000, Thermo Fisher Scientific Inc, Wilmington, DE, USA). All the DNA samples had ABS_{260}/ABS_{280} ratios of 1.8–2.0. Illumina HiSeqX Platform (Illumina, San Diego, USA) was used to sequence the DNA samples.

Sequencing and sequence filtering

A total of 77 gut microbiome were sequenced from swine and cattle for this study. For every sample, 151-bp paired-end sequences were generated from the insert of 350 base pairs. An average of 160 M paired-reads (ranging between 110 M and 229 M) were generated for each sample after filtering. Low-quality reads were removed using Sickle [25], and reads containing “N” were also removed.

Finally, host contamination was removed by discarding the reads that were mapped to the swine and cattle genomes provided by NCBI.

Antimicrobial susceptibility testing

Samples were processed, and *E. coli* was isolated as described previously [24] using eosin methylene blue agar (Becton Dickinson, Sparks, MD 21152, USA) and MacConkey agar plates (BD). Species identification was performed by matrix-assisted laser desorption ionization time-of-flight mass spectrometry (bioMérieux, Marcy l'Étoile, France).

Antimicrobial susceptibility was assessed by determining the minimum inhibitory concentrations (MICs) for 16 antimicrobial agents using the broth microdilution method with a commercially available Sensititre® panel KRVP4F (TREK Diagnostic Systems, West Sussex, UK) according to the manufacturer's instructions. The following antibiotics were tested: ampicillin, amoxicillin/clavulanic acid, cefoxitin, ceftiofur, ceftazidime, cefepime, chloramphenicol, ciprofloxacin, colistin, gentamicin, meropenem, nalidixic acid, streptomycin, sulfisoxazole, tetracycline and trimethoprim/sulfamethoxazole. The reference strain *E. coli* ATCC 25922 was used as quality control when determining MICs. The interpretation of MIC was carried out according to the Clinical and Laboratory Standards Institute (CLSI) guidelines (CLSI, 2017). When CLSI breakpoints were not available, the MIC interpretation was carried out according to the Danish Integrated Antimicrobial Resistance Monitoring and Research Programme (DANMAP, 2014). Multidrug resistance was defined as resistance to three or more antibiotic subclasses.

Identification of antibiotic resistance genes

To screen the antibiotic resistance genes in the microbiome, a three-step procedure was performed. First, filtered reads were assembled into contigs using MEGAHIT [26], a *de novo* assembler based on *de Bruijn* graph, with default options. Only contigs of length > 500 bp were used for gene prediction. To predict genes from contigs, FragGeneScan [27] was applied with the options of no sequencing errors (-w 0 –t complete). Lastly, genes predicted in the metagenomic data set were aligned with the antibiotic resistance genes annotated in the CARD database [18]. CARD version 2.0.1 includes a total of 2,252 protein sequences. Antibiotic resistance genes from uncultured bacteria and the genes annotated as regulatory systems or efflux pump related were excluded. The resistance genes were classified into 21 ARG classes based on the gene ontology [18]. We added MLS classes (lincosamide, macrolide, and streptogramin shared) that have Cfr 23S and Erm 23S as subclass because these two subclass are commonly found in the three classes. Blastp [28] was used for ARG profiling with an e-value threshold of 1×10^{-10}, similarity exceeding 70%, and reference coverage over 70%.

Subsequently, normalization was performed as the number of antibiotic resistance genes with respect to the number of genes predicted in each sample:

$$\text{GPM (Gene Per Million)} = \frac{\text{Number of antibiotic resistane genes annotated}}{\text{Number of genes predicted}} \times 10^6.$$

To find the taxonomic composition of each sample, MetaPhlAn was used to map the filtered reads onto clade-specific marker genes.

Statistical analysis and network analysis

To compare the composition of bacteria and resistance genes between swine and cattle, PCA was carried out. In addition, t-test was performed to identify the key factors that designate the antibiotic resistance rate in swine and cattle samples. Correlation analysis between resistance classes and genus was also conducted by using R package.

Network analysis was performed to find the origin of each resistance gene annotated in the samples. For this purpose, all the genes of 8,369 complete genomes downloaded from the NCBI repository (https://www.ncbi.nlm.nih.gov/) were searched against the antibiotic resistance genes in CARD [18]. A network graph was built with the nodes of antibiotic resistance genes. Colors of the nodes represent either sample or host genus. The nodes were connected with a full line, if the two nodes met the thresholds of e-value less than 1×10^{-10}, similarity of 100% with 100% coverage. The dotted line represents 70% or more similarity. The dotted lines were drawn after measuring the similarity between a genes from each cluster and an annotated gene from CARD database if the cluster does not include any annotated genes in CARD.

Table 1. Antibiotic resistance of *Escherichia coli* (*n* = 146) isolated from animal faecal samples

Antimicrobial subclass	Antimicrobial agents	Breakpoint (μg/ml)	swine (n=36)			Cattle (n=41)		
			MIC_{50} (μg/ml)	MIC_{90} (μg/ml)	Resistance % (no.)	MIC_{50} (μg/ml)	MIC_{90} (μg/ml)	Resistance % (no.)
Aminoglycosides	Gentamicin (GEN)	≥16	1	32	27.8(10)	1	1	0(0)
	Streptomycin (STR)	≥32	64	128	66.7(24)	16	64	28.2(11)
Aminopenicillin	Ampicillin (AMP)	≥32	64	64	69.4(25)	4	4	0(0)
β-lactam/-lactamase inhibitor combinations	Amoxicillin/ clavulanic acid (AmC)	≥32	8	8	0(0)	2	4	0(0)
Cephamycin	Cefoxitin (FOX)	≥32	4	8	0(0)	4	4	0(0)
Cephalosporin Ⅲ	Ceftiofur (XNL)	≥8	0.5	0.5	2.8(1)	0.5	0.5	0(0)
	Ceftazidime (CAZ)	≥16	1	1	0(0)	1	1	0(0)
Cephalosporin Ⅳ	Cefepime (FEP)	≥16	0.25	0.25	0(0)	0.25	0.25	0(0)
Carbapenem	Meropenem (MEM)	≥4	0.25	0.25	0(0)	0.25	0.25	0(0)
Fluoroquinolone	Ciprofloxacin (CIP)	≥4	0.25	8	16.7(6)	0.12	0.25	0(0)
Folate pathway inhibitors	Trimethoprim/ Sulfamethoxazole (SXT)	≥4	0.25	4	33.3(12)	0.12	0.12	0(0)
Sulfonamides	Sulfisoxazole (FIS)	≥512	512	512	66.7(24)	32	512	28.2(11)
Phenicols	Chloramphenicol (CHL)	≥32	64	64	66.7(24)	8	8	0(0)
Polymyxins	Colistin (COL)	≥4	2	2	0(0)	2	2	0(0)
Quinolone	Nalidixic acid (NAL)	≥32	8	128	33.3(12)	2	64	12.8(5)
Tetracyclines	Tetracycline (TET)	≥16	64	128	66.7(24)	2	128	43.6(17)

MIC_{50} and MIC_{90} are the concentrations at which 50% and 90% of the isolates were inhibited

Availability of supporting data and materials

Table S1. Sequencing data information for swine and cattle gut microbiome

Table S2. Sample information for cattle

Table S3. Sample information for swine

Declaration

List of abbreviations

ARG: Antibiotic resistance gene; PCA: Principle component analysis; GPM: Genes per million genes predicted; MLS: macrolide-lincosamide-streptogramin shared; APH: Aminoglycoside phosphotransferase; ANT: aminoglycoside nucleotidyltransferase; AAC: aminoglycoside acetyltransferase

Consent for publication

Not applicable.

Competing interests

The authors declare that they have no competing interests.

Funding

This research was supported by a grant (2017NER54070 to SL and MR) by Research of Korea Centers for Disease Control and Prevention.

Authors' contributions

SL and MR conceived and designed the study. SL performed the experiment and analysis. DK, YC, and MR performed the analysis. DM prepared the samples and performed experiment. SL, DK, DM, YC, and MR wrote the manuscript. All authors read and approved the final manuscript.

Acknowledgements

We thank Drs. Hyunjoo Pai, Jieun Kim, Chang-Jun Cha, and Inho Park for helpful discussion.

Reference

1. Allen, H.K., et al., *Call of the wild: antibiotic resistance genes in natural environments.* Nat Rev Microbiol, 2010. **8**(4): p. 251-9.
2. Smillie, C.S., et al., *Ecology drives a global network of gene exchange connecting the human microbiome.* Nature, 2011. **480**(7376): p. 241-4.
3. Forslund, K., et al., *Country-specific antibiotic use practices impact the human gut resistome.* Genome Res, 2013. **23**(7): p. 1163-9.
4. Hu, Y.F., et al., *Metagenome-wide analysis of antibiotic resistance genes in a large cohort of human gut microbiota.* Nature Communications, 2013. **4**.
5. Zhu, Y.G., et al., *Diverse and abundant antibiotic resistance genes in Chinese swine farms.* Proc Natl Acad Sci U S A, 2013. **110**(9): p. 3435-40.
6. Pal, C., et al., *The structure and diversity of human, animal and environmental resistomes.* Microbiome, 2016. **4**(1): p. 54.
7. Sommer, M.O.A., G. Dantas, and G.M. Church, *Functional Characterization of the Antibiotic Resistance Reservoir in the Human Microflora.* Science, 2009. **325**(5944): p. 1128-1131.
8. Forsberg, K.J., et al., *The shared antibiotic resistome of soil bacteria and human pathogens.* Science, 2012. **337**(6098): p. 1107-11.
9. Wichmann, F., et al., *Diverse antibiotic resistance genes in dairy cow manure.* MBio, 2014. **5**(2): p. e01017.
10. Xiao, L., et al., *A reference gene catalogue of the pig gut microbiome.* Nature Microbiology, 2016. **1**: p. 16161.
11. Noyes, N.R., et al., *Characterization of the resistome in manure, soil and wastewater from dairy and beef production systems.* Sci Rep, 2016. **6**: p. 24645.
12. Shoemaker, N.B., et al., *Evidence for extensive resistance gene transfer among Bacteroides spp. and among Bacteroides and other genera in the human colon.* Appl Environ Microbiol, 2001. **67**(2): p. 561-8.
13. Gerzova, L., et al., *Characterization of Antibiotic Resistance Gene Abundance and Microbiota Composition in Feces of Organic and Conventional Pigs from Four EU Countries.* PLoS One, 2015. **10**(7): p. e0132892.
14. Quan, J., et al., *A global comparison of the microbiome compositions of three gut locations in commercial pigs with extreme feed conversion ratios.* Sci Rep, 2018. **8**(1): p. 4536.
15. Rice, W.C., et al., *Influence of wet distillers grains diets on beef cattle fecal bacterial community structure.* BMC Microbiol, 2012. **12**: p. 25.
16. Shanks, O.C., et al., *Community structures of fecal bacteria in cattle from different animal feeding operations.* Appl Environ Microbiol, 2011. **77**(9): p. 2992-3001.
17. Human Microbiome Project, C., *Structure, function and diversity of the healthy human microbiome.* Nature, 2012. **486**(7402): p. 207-14.
18. McArthur, A.G., et al., *The comprehensive antibiotic resistance database.* Antimicrob Agents Chemother, 2013. **57**(7): p. 3348-57.
19. Ueda, O., et al., *Sixteen homologs of the mex-type multidrug resistance efflux pump in Bacteroides fragilis.* Antimicrob Agents Chemother, 2005. **49**(7): p. 2807-15.
20. Kroeger, J.K., et al., *Bacillus cereus e ffl ux protein BC3310 - a multidrug transporter of the unknown major facilitator family, UMF-2.* Front Microbiol, 2015. **6**: p. 1063.
21. Elander, R.P., *Industrial production of beta-lactam antibiotics.* Appl Microbiol Biotechnol, 2003. **61**(5-6): p. 385-92.
22. Mingeot-Leclercq, M.P., Y. Glupczynski, and P.M. Tulkens, *Aminoglycosides: activity and resistance.* Antimicrob Agents Chemother, 1999. **43**(4): p. 727-37.

23. Li, B., et al., *Metagenomic and network analysis reveal wide distribution and co-occurrence of environmental antibiotic resistance genes.* ISME J, 2015. **9**(11): p. 2490-502.
24. Nam, H.M., et al., *Prevalence of antimicrobial resistance in fecal Escherichia coli isolates from stray pet dogs and hospitalized pet dogs in Korea.* Microb Drug Resist, 2010. **16**(1): p. 75-9.
25. Joshi NA, F.J., *Sickle: A sliding-window, adaptive, quality-based trimming tool for FastQ files (Version 1.33) [Software]. Available at* https://github.com/najoshi/sickle. 2011.
26. Li, D., et al., *MEGAHIT: an ultra-fast single-node solution for large and complex metagenomics assembly via succinct de Bruijn graph.* Bioinformatics, 2015. **31**(10): p. 1674-6.
27. Rho, M., H. Tang, and Y. Ye, *FragGeneScan: predicting genes in short and error-prone reads.* Nucleic Acids Res, 2010. **38**(20): p. e191.
28. Altschul, S.F., et al., *Basic local alignment search tool.* J Mol Biol, 1990. **215**(3): p. 403-10.

Figure Legend

Figure 1. Bacterial composition of the swine and cattle gut microbiome. Genus-level bacterial composition in (A) swine and (B) cattle. The ten most abundant bacterial phyla in (C) swine and (D) cattle. The ten most abundant bacterial genus in (E) swine and (F) cattle.

Figure 2. Different bacterial compositions in the gut microbiome of swine and cattle. (A) PCA of genus-level bacterial composition in swine and cattle. (B) Differential distribution of genus composition in swine and cattle (p-value < 0.01; the median relative abundance in any sample > 1%).

Figure 3. Composition of antibiotic resistance genes in swine and cattle. (A) PCA with the abundance of antibiotic resistance determinants in swine and cattle. (B) The distribution of significant antibiotic resistance determinants in swine and cattle (p-value < 0.01).

Figure 4. Antibiotic resistance genes in swine and cattle gut microbiome. Binary heatmap showing the presence of the resistance genes for (A) aminoglycoside, (C) tetracycline, and (E) beta-lactam. The five most abundant gene families of antibiotic resistance genes for (B) aminoglycoside, (D) tetracycline, and (F) beta-lactam. The y-axis represents the number of antibiotic resistance genes per million genes predicted.

Figure 5. Network analysis of resistance genes and their similarity in the swine and cattle gut microbiome. Network of (A) ANT(6), (B) APH(3'), (C) tetQ, (D) tetX, (E) tetM. The nodes in the network are resistance genes identified in swine (blue) and cattle (red), stored in CARD database (orange) and the bacterial complete genome (yellow). The solid line represents 100% similarity and the dotted line represents 70% or more similarity. The dotted lines were drawn after measuring the similarity between a genes from each cluster and an annotated gene from CARD database if the cluster does not include any annotated genes in CARD.

Figure

Click here to download Figure AR_Farm_Animals_Figure_1001.pptx

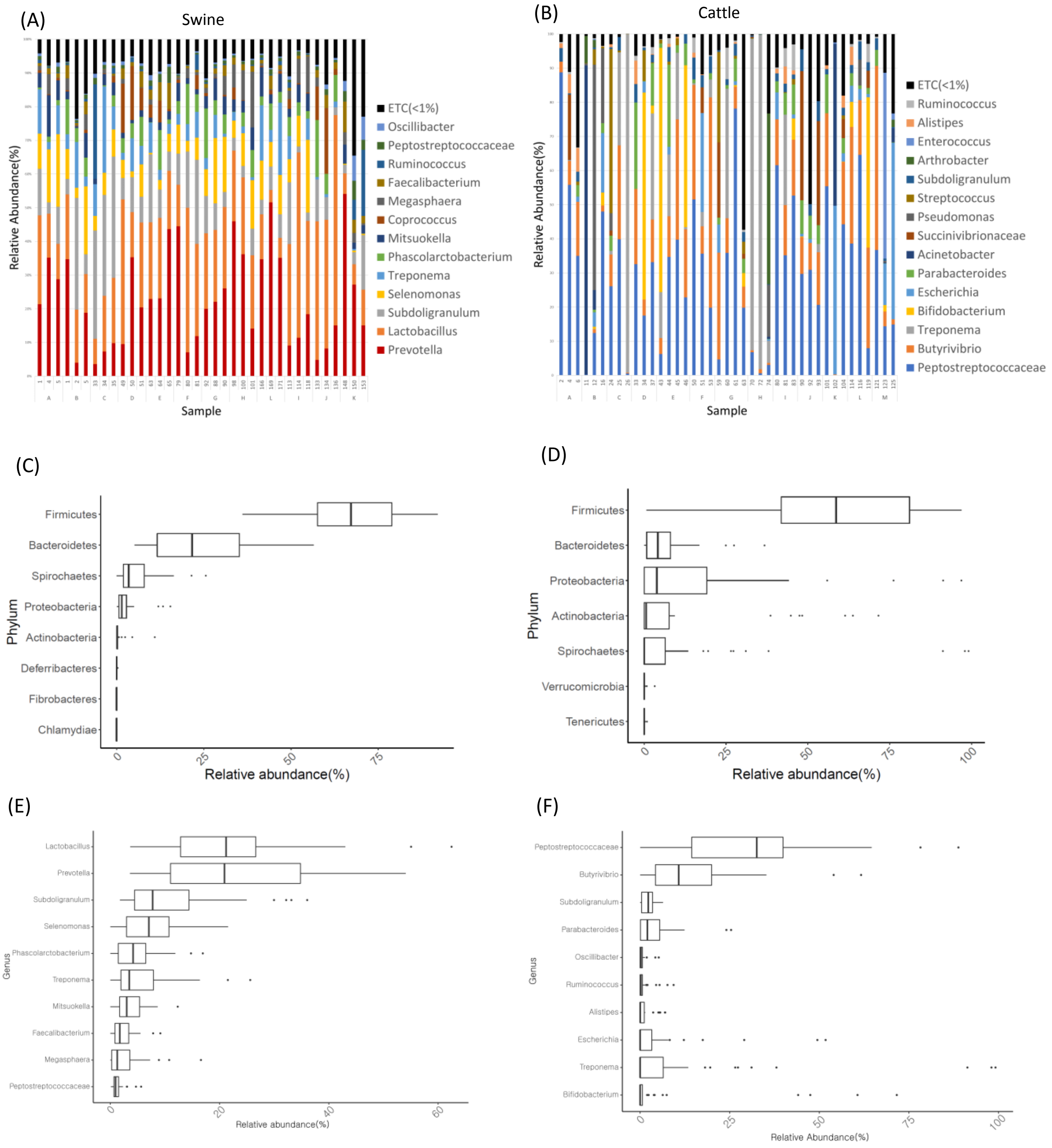

Figure 1. Bacterial composition of the swine and cattle gut microbiome. Genus-level bacterial composition in (A) swine and (B) cattle. The ten most abundant bacterial phyla in (C) swine and (D) cattle. The ten most abundant bacterial genus in (E) swine and (F) cattle.

(A) (B)

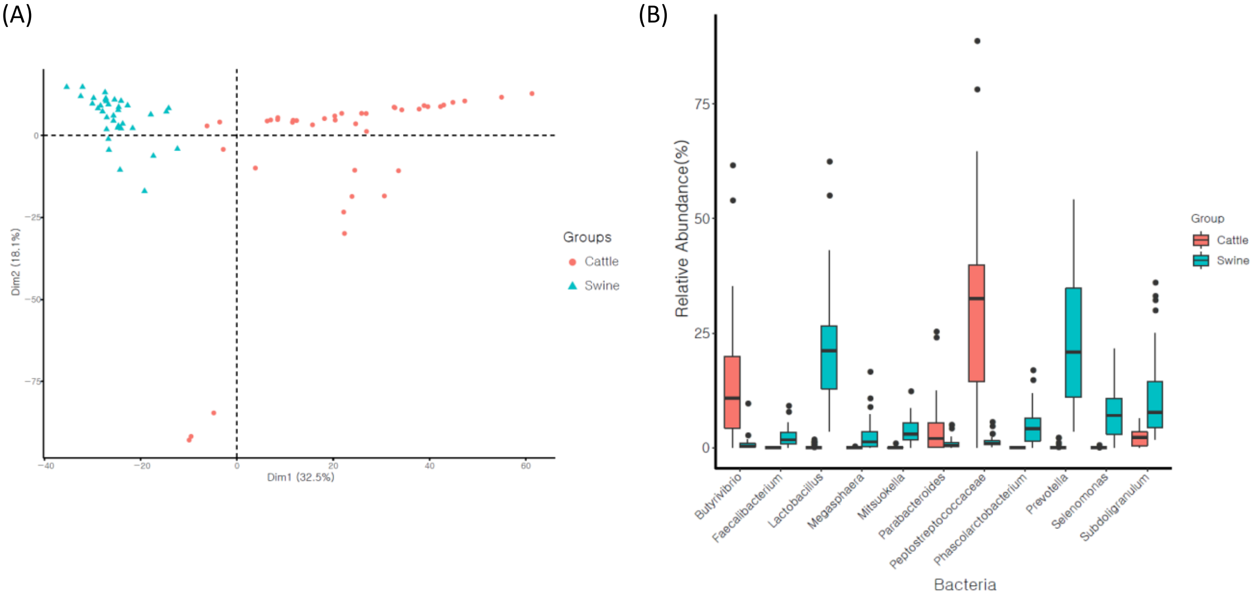

Figure 2. Different bacterial compositions in the gut microbiome of swine and cattle. (A) PCA of genus-level bacterial composition in swine and cattle. (B) Differential distribution of genus composition in swine and cattle (p-value < 0.01; the median relative abundance in any sample > 1%).

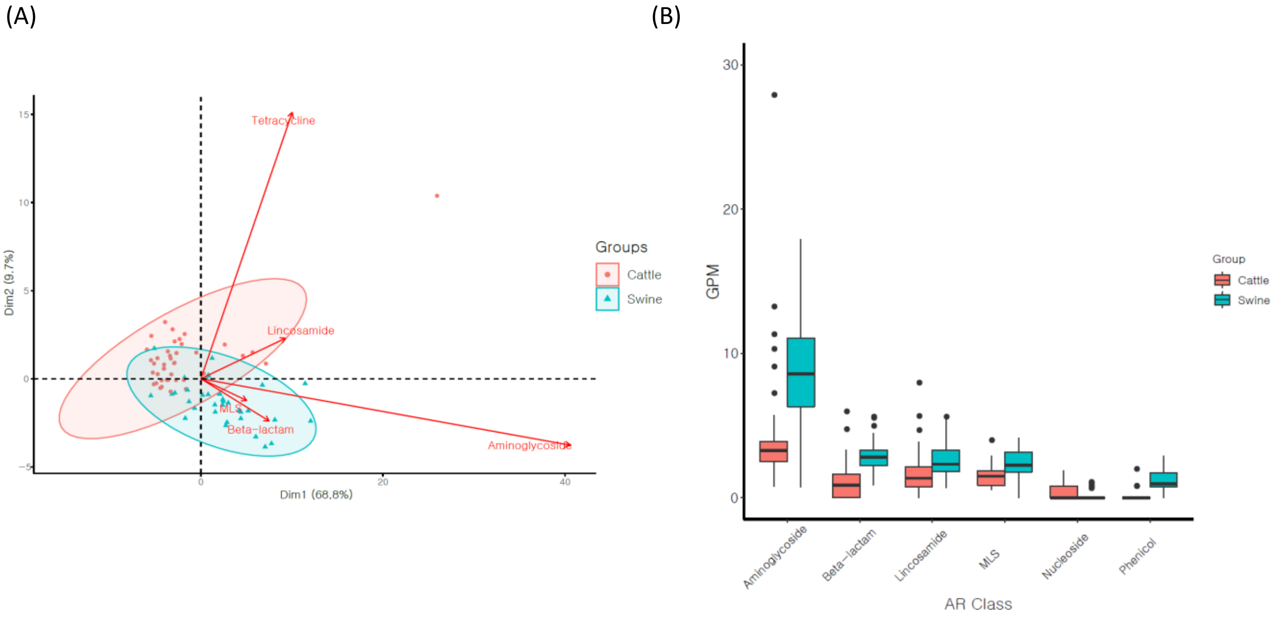

Figure 3. Composition of antibiotic resistance genes in swine and cattle. (A) PCA with the abundance of antibiotic resistance determinants in swine and cattle. (B) The distribution of significant antibiotic resistance determinants in swine and cattle (p-value < 0.01).

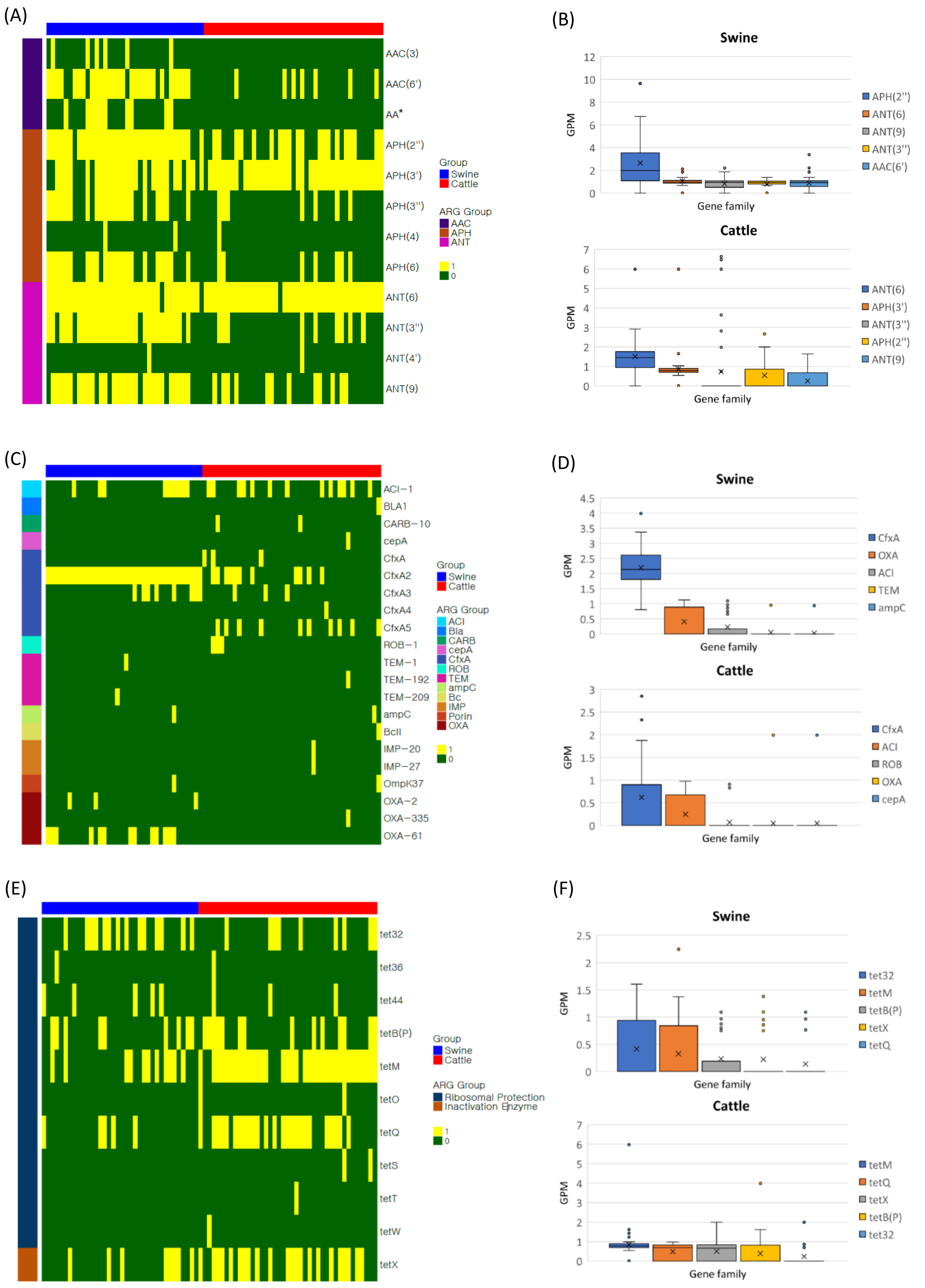

Figure 4. Antibiotic resistance genes in swine and cattle gut microbiome. Binary heatmap showing the presence of the resistance genes for (A) aminoglycoside, (C) beta-lactam, and (E) tetracycline. The five most abundant gene families of antibiotic resistance genes for (B) aminoglycoside, (D) beta-lactam, and (F) tetracycline. The y-axis represents the number of antibiotic resistance genes per million genes predicted.

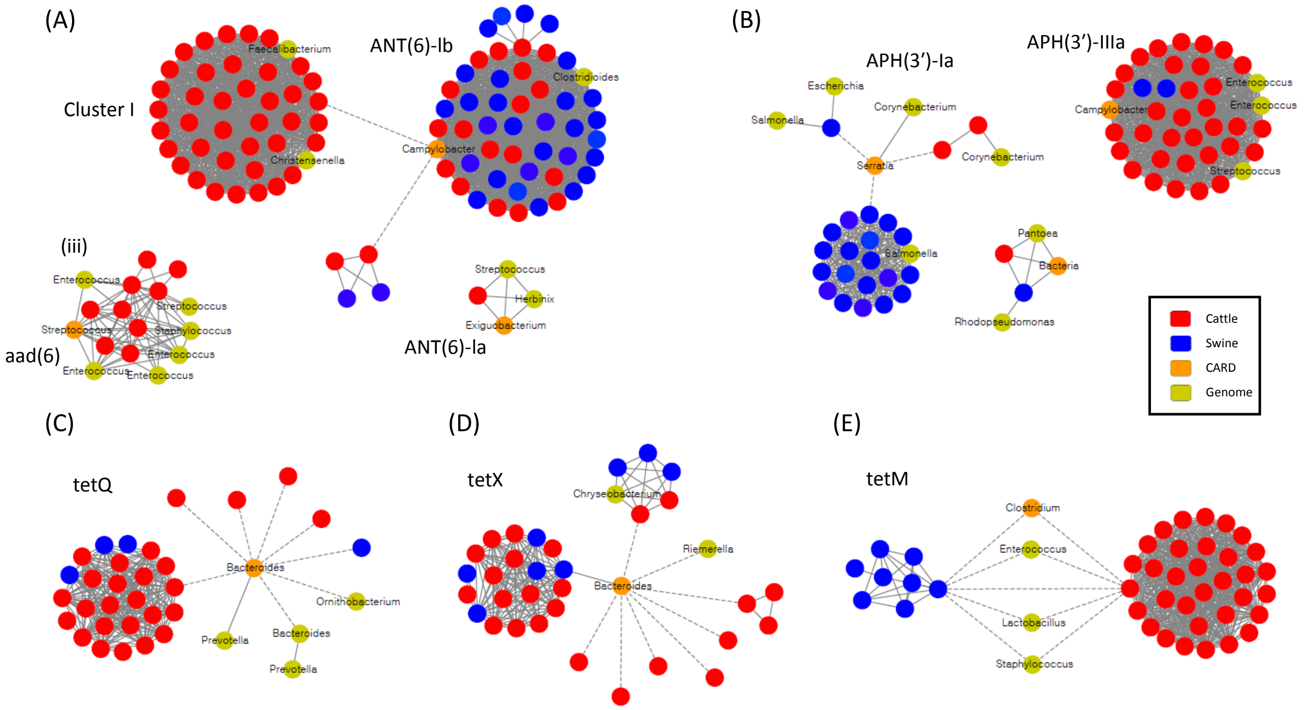

Figure 5. Network analysis of resistance genes and their similarity in the swine and cattle gut microbiome. Network of (A) ANT(6), (B) APH(3'), (C) tetQ, (D) tetX, (E) tetM. The nodes in the network are resistance genes identified in swine (blue) and cattle (red), stored in CARD database (orange) and the bacterial complete genome (yellow). The solid line represents 100% similarity and the dotted line represents 70% or more similarity. The dotted lines were drawn after measuring the similarity between a genes from each cluster and an annotated gene from CARD database if the cluster does not include any annotated genes in CARD.

Supplementary Figure

Click here to access/download
Supplementary Material
AR_Farm_Animals_Supplementary_Figure_1001.pptx

Supplementary Table

Click here to access/download
Supplementary Material
AR_Farm_Animals_Supplementary_Table_1001.docx
