# Supplementary material for: Antibiotic resistomes discovered in the gut microbiomes of Korean swine and cattle
Source: Gigascience. 2020 May 5;9(5):giaa043. doi: 10.1093/gigascience/giaa043 (PMC7317084; doi:10.1093/gigascience/giaa043)
Supplement: giaa043_GIGA-D-19-00340_Revision_2 [file giaa043_giga-d-19-00340_revision_2.pdf]

GigaScience

Antibiotic resistomes discovered in the gut microbiomes of swine and cattle

--Manuscript Draft--

**Manuscript Number:**	GIGA-D-19-00340R2
**Full Title:**	Antibiotic resistomes discovered in the gut microbiomes of swine and cattle
**Article Type:**	Research
**Funding Information:**	Korea Centers for Disease Control and Prevention (2017NER54070) — Dr. Suk-Kyung Lim; Dr Mina Rho
**Abstract:**	ABSTRACT<br>Background: Antibiotics administered to farm animals have led to increasing prevalence of resistance genes in different microbiomes and environments. While antibiotic treatments help cure infectious diseases in farm animals, the possibility of spreading antibiotic resistance genes into the environment and human microbiomes raises significant concerns. Through long-term evolution, antibiotic resistance genes have mutated, thereby complicating the resistance problems.<br>Results: In this study, we performed deep sequencing of the gut microbiomes of 36 swine and 41 cattle in Korean farms, and metagenomic analysis to understand the diversity and prevalence of antibiotic resistance genes. We found that aminoglycoside, beta-lactam, lincosamide, streptogramin, and tetracycline were the prevalent resistance determinants in both swine and cattle. Tetracycline resistance was abundant and prevalent in cattle and swine. Specifically, tetQ , tetW , tetO , tet32 , and tet44 were the five most abundant and prevalent tetracycline resistance genes. Their prevalence was almost 100% in swine and cattle. While tetQ was similarly abundant in both swine and cattle, tetW was more abundant in swine than in cattle. Aminoglycoside was the second highest abundant resistance determinant in swine, but not in cattle. In particular, ANT(6) and APH(3′′) were the dominant resistance gene families in swine. Beta-lactam was also an abundant resistance determinant in both swine and cattle. Cfx was the major contributing gene family conferring resistance against beta-lactams.<br>Conclusions: Antibiotic resistome was more pervasive in swine than in cattle. Specifically, prevalent ARGs (prevalence > 50%) were found more in swine than in cattle, Genomic investigation of specific resistance genes from the gut microbiomes of swine and cattle in this study should provide opportunities to better understand the exchange of antibiotic resistance genes in farm animals.
**Corresponding Author:**	Mina Rho<br><br>KOREA, REPUBLIC OF
**Corresponding Author Secondary Information:**	
**Corresponding Author's Institution:**	
**Corresponding Author's Secondary Institution:**	
**First Author:**	Suk-Kyung Lim
**First Author Secondary Information:**	
**Order of Authors:**	Suk-Kyung Lim<br>Dongjun Kim<br>Dong-Chan Moon<br>Youna Cho<br>Mina Rho
**Order of Authors Secondary Information:**	
**Response to Reviewers:**	Reviewer #2: I am happy to find, that the authors have more or less addressed all my concerns. I have comments for some of their rebuttals and for some of the changes to the manuscript. Also, the authors / journal might want to specify in the paper title reflect that

the scope is Korea, like the authors have now done throughout the manuscript.
Answer to author rebuttal:

1 Looks like a fine approach. What was the difference between the first and second filtering threshold? Could a single "70% pass" give the same result?

Answer) When we compared the number of reads in four samples with and without 70% similarity threshold, there was no difference.

3 I am not going to insist the authors use CODA methods, but as I explained further down, I have some specific objections to their (old and new) analysis, where I don't think it makes sense and could be helped using alternatives suggested by Gloor et al. Also as far as I can see, the transition to CLR is not meaningful when coupled with their chosen analysis.

Answer) Following the reviewer's helpful suggestion, we have performed the correlation analysis with SPARCC. The revised manuscript reports the results in line 396 and lines 112–113, as well as Supplementary Figure S1.

Specific comments:

L31: Language could be a bit more precise in conclusion and throughout. Does "more diverse" refer to a higher alpha diversity / evenness in samples from one livestock species or to a larger beta-diversity within one livestock species compared to the other?

Answer) We used the expression "more diverse" to explain that more genes are prevalent in swine than in cattle. Specifically, for the prevalent ARGs (prevalence > 50%), we found 32 genes in swine, while 19 genes in cattle. We revised the sentence to read "Antibiotic resistome was more pervasive in swine than in cattle. Specifically, prevalent ARGs (prevalence > 50%) were found more in swine than in cattle".

L92: On my request, the authors have now added the information on the actual sequencing instrument (HiSeq4000) which ENA also states. However, the MS also still refers to the HiSeqX. It also still says minimum was 110M PE reads. These discrepancies should be fixed.

Answer) Corrections were made accordingly.

L112: A negative correlation coefficient is given. Is it significant? Prevotella was very common, so it is not surprising there are lower proportions of other bacteria in samples with a lot of Prevotella. See further down, why the test probably does not yield meaningful results.

Answer) In response to the reviewer's suggestion, we performed the correlation analysis with SPARCC. We note that the proportion of Prevotella still showed a negative correlation with that of Subdoligranulum (r = –0.6457).

L169: Were the values normally distributed so the results of t-tests are valid and meaningful? If median cattle phenicol AMR was 0 RPKM, then it seems unlikely? If the authors log transform data (after zero replacement), they will get ALR values which are more likely to be normal.

Answer) The normality test showed that several ARGs including phenicol are not normally distributed. We thus removed the t-test results and just compared RPKM values for abundance between swine and cattle.

L382 "The genus proportion (>0.1%) was used for calculating Shannon index…". Is it correctly understood that rare genera were excluded? That will impact the diversity indexes and seems like it should be justified. I am not convinced its required, especially if the sample was also rarefied (a practice there is some controversy around: https://doi.org/10.1371/journal.pcbi.1003531).

Answer) In response to the reviewer's suggestion, we also calculated the index with the genus without any proportion threshold (Supplementary Figure S4D–F). The result showed the same pattern. The method section was also revised; please see lines 390–392.

Powered by Editorial Manager® and ProduXion Manager® from Aries Systems Corporation

	L388: If the authors use rank-based correlation (Spearman), CLR transformation will not have any effect. If the authors want to implement some of the CODA principles from the papers, this is not a good place, when coupled with a rank-based correlation. Rank-based does not solve the issue of negative correlation bias. In the Gloor paper I linked (GB Gloor et al, 2017), they say e.g. the following:<br>"There are several more rigorous approaches that can be applied to analyze correlation in microbiome datasets, including SPARCC (Friedman and Alm, 2012) and SPieCeasi (Kurtz et al., 2015), both of which assume a sparse data matrix, and the ϕ (Lovell et al., 2015) and ρ…"<br><br>Answer) In response to the reviewer's suggestion, we performed correlation analysis using SPARCC and revised the manuscript. Please see line 396 and lines 112–113, as well as Supplementary Figure S1.<br><br>Supplementary:<br><br>Some of the captions are very short and uninformative.<br>Answer) Corrections were made accordingly.<br><br>The authors claim they do not have estimates of the size of Korean pig and cattle production. Figure S5 has (antibiotic sales in mg/PCU + 1). I am not sure how the authors have managed to adjust the drug use to the size of animal production then. "1 PCU = 1 kilogram of biomass of different categories of livestock and slaughtered animals". This needs explaining and if it is not adjusted to production size / number of animals / meat produced / sold or similar, then than sub-plot can be removed. The lines being higher on the Y axis in one animal is then not very informative.<br><br>Answer) The caption for Figure S5(A) was corrected to read "the amount of antibiotics sold (ton)", which is consistent with Figure S3.
**Additional Information:**	
**Question**	**Response**
Are you submitting this manuscript to a special series or article collection?	No
**Experimental design and statistics**<br><br>Full details of the experimental design and statistical methods used should be given in the Methods section, as detailed in our Minimum Standards Reporting Checklist. Information essential to interpreting the data presented should be made available in the figure legends.<br><br>Have you included all the information requested in your manuscript?	Yes
**Resources**<br><br>A description of all resources used, including antibodies, cell lines, animals and software tools, with enough information to allow them to be uniquely identified, should be included in the Methods section. Authors are strongly encouraged to cite Research Resource Identifiers (RRIDs) for antibodies, model organisms and tools, where possible.<br><br>Have you included the information requested as detailed in our Minimum Standards Reporting Checklist?	Yes

**Availability of data and materials**<br><br>All datasets and code on which the conclusions of the paper rely must be either included in your submission or deposited in publicly available repositories (where available and ethically appropriate), referencing such data using a unique identifier in the references and in the "Availability of Data and Materials" section of your manuscript.<br><br>Have you have met the above requirement as detailed in our Minimum Standards Reporting Checklist?	Yes

Powered by Editorial Manager® and ProduXion Manager® from Aries Systems Corporation

Manuscript with DOI

Click here to access/download;Manuscript;AR_Farm_Animals_0316 with DOI.docx

Antibiotic resistomes discovered in the gut microbiomes of Korean swine and cattle

Suk-Kyung Lim[1], Dongjun Kim[2], Dong-Chan Moon[1], Youna Cho[2], Mina Rho[2,3]

[1]Bacterial Disease Division, Animal and Plant Quarantine Agency, Gimcheon, Korea
[2]Department of Computer Science and Engineering, Hanyang University, Seoul, Korea
[3]Department of Biomedical Informatics, Hanyang University, Seoul, Korea

To whom correspondence should be addressed: Mina Rho (Tel: 82-2-2220-2379; Email: minarho@hanyang.ac.kr).

ABSTRACT

Background: Antibiotics administered to farm animals have led to increasing prevalence of resistance genes in different microbiomes and environments. While antibiotic treatments help cure infectious diseases in farm animals, the possibility of spreading antibiotic resistance genes into the environment and human microbiomes raises significant concerns. Through long-term evolution, antibiotic resistance genes have mutated, thereby complicating the resistance problems.

Results: In this study, we performed deep sequencing of the gut microbiomes of 36 swine and 41 cattle in Korean farms, and metagenomic analysis to understand the diversity and prevalence of antibiotic resistance genes. We found that aminoglycoside, beta-lactam, lincosamide, streptogramin, and tetracycline were the prevalent resistance determinants in both swine and cattle. Tetracycline resistance was abundant and prevalent in cattle and swine. Specifically, *tetQ*, *tetW*, *tetO*, *tet32*, and *tet44* were the five most abundant and prevalent tetracycline resistance genes. Their prevalence was almost 100% in swine and cattle. While *tetQ* was similarly abundant in both swine and cattle, *tetW* was more abundant in swine than in cattle. Aminoglycoside was the second highest abundant resistance determinant in swine, but not in cattle. In particular, *ANT(6)* and *APH(3″)* were the dominant resistance gene families in swine. Beta-lactam was also an abundant resistance determinant in both swine and cattle. *Cfx* was the major contributing gene family conferring resistance against beta-lactams.

Conclusions: Antibiotic resistome was more pervasive in swine than in cattle. Specifically, prevalent ARGs (prevalence > 50%) were found more in swine than in cattle, Genomic investigation of specific resistance genes from the gut microbiomes of swine and cattle in this study should provide opportunities to better understand the exchange of antibiotic resistance genes in farm animals.

Keywords: Swine gut microbiomes, Cattle gut microbiomes, Antibiotic resistome, Antibiotic resistance gene

BACKGROUND

Antibiotics have been widely used to cure infectious diseases. In farms, antibiotics have also been used to treat and prevent diseases or to promote the growth of animals. Increasing administration of antibiotics expedites the development of resistance, and leads to the spread of resistance genes in the farm environment and human population [1]. Moreover, gene transfer from the environment or food chain to the human population further complicates this problem. In particular, antibiotic resistance genes were transferred from one bacterium to others within the human microbiomes, and between human and livestock microbiomes [2].

With advances in high-throughput sequencing technology and metagenomic analysis, gut microbiomes have been investigated to understand the prevalence of antibiotic resistance genes (ARGs) and the compositional changes in the microbiomes after treatment. In recent years, ARGs have been extensively studied to understand their diversity and abundance in the human microbiomes in terms of the race and age [3, 4]. The most prevalent resistance determinant in humans is tetracycline [4], which is also prevalent in farm animals [5]. Since tetracycline is widely applied for infection control and growth promotion, several studies have suggested a positive correlation between its usage and prevalence [6]. In human skin microbiomes [7] and soil microbiomes [8], divergent ARGs that show low sequence similarity to the known genes have also been identified through functional metagenomics, implying that resistance genes have evolved in diverse environments.

For the gut microbiomes of farm animals, several studies have explored the prevalence of antibiotic resistance genes [5, 9]. A recent study on ARGs in Chinese, French, and Danish swine showed that the most prevalent classes of ARGs are tetracycline, beta-lactam, macrolide, streptogramin, and bacitracin [10]. Notably, the profile of ARGs in Chinese swine was different from that in the other two populations, in terms of the composition and abundance. Tetracycline, aminoglycoside, and beta-lactam were also the abundant antibiotic classes in the farm environments for swine [5], which was consistent with the ARG profiles of farm animals. The effects of antibiotics, used as feed additives, on the changes in bacterial composition was discussed. In the cattle microbiomes, it was found that tetracycline was the most abundant class, followed by aminoglycoside [11]. A previous study suggested that ARGs in the animal microbiomes could be transferred and distributed to other environments [12].

In this work, we performed metagenomic analysis on the gut microbiomes of swine and cattle to investigate the diversity and prevalence of ARGs in different farm environments. An unbiased screening of microbial resistance genes was performed using the metagenomic shotgun sequencing data. To our knowledge, this is the first study investigating ARGs in multiple types of farm animals

raised in Korea. We observed the presence of two different patterns of resistance genes: one type is host-dedicated; the other exists in different host animals.

DATA DESCRIPTION

We performed deep sequencing on the gut microbiomes of 36 swine and 41 cattle in the Korean farms (Supplementary Table S1), and metagenomic analysis to understand the diversity and prevalence of ARGs. All raw sequencing data described in this study are available at the European Nucleotide Archive (ENA) with the accession number PRJEB32496.

Fresh fecal samples from healthy finishing swine and adult Korean cattle were collected aseptically in 25 feedlots throughout Korea between August 2017 and June 2018 (Supplementary Table S2 and S3), following the guidelines of the Animal Protection Act of Animal and Plant Quarantine Agency. Farm selection was based on two criteria: geographical distribution and farm size.

The Illumina HiSeq4000 Platform (Illumina, San Diego, USA) was used to sequence the DNA samples. A total of 77 gut microbiomes were sequenced from swine and cattle for this study. For every sample, 151-bp paired-end sequences were generated from the insert of 350 base pairs. An average of 38 M paired-reads (ranging between 25 M and 75 M) were generated for each sample after filtering.

ANALYSIS

Bacterial composition of swine and cattle gut microbiomes

A total of 36 gut microbiomes from swine and 41 from cattle were collected to investigate their bacterial composition. Consistent with the previous studies [13-16], the major phyla in swine and cattle were Bacteroidetes and Firmicutes, which were also commonly observed in human gut microbiomes [17]. Their proportions, however, were quite different in two animals: 21.65% and 67.16 %, respectively, in swine; 4.15% and 58.63%, respectively, for cattle (Figure 1C and D). The ratio of Bacteroidetes to Firmicutes was much higher in swine than in cattle.

In the swine gut microbiomes, the major genera were *Lactobacillus* (21.19%; median proportion), *Prevotella* (20.89%), *Subdoligranulum* (7.75%), and *Selenomonas* (7.06%) (Figure 1A and E). *Prevotella* was the major genus in Bacteroidetes, whereas *Lactobacillus, Subdoligranulum,* and *Selenomonas* were the major genera in Firmicutes. The proportion of *Prevotella* showed a negative correlation with that of *Subdoligranulum* (r = -0.6457; Figure 1A and Supplementary Figure S1A). At the species level, *Prevotella copri* was the most abundant species, which comprised 17.23% of the

microbiomes (Supplementary Figure 2C). This value was higher than that of other species, such as *Lactobacillus. amylovorus* (7.80%), *Subdoligranulum* species (7.75%), and *Streptococcus. bovis* (7.06%).

In the cattle microbiomes, the major genera were the *Peptostreptococcaceae* genus (32.56%; median proportion) and *Butyrivibrio* (10.77%), which were observed in all cattle samples. Even though *Treponema* and *Bifidobacterium* were observed as two of the ten most abundant genera ordered based on their average proportions, their prevalence was lower than 50%, i.e., they were seen in 19 and 14 out of the 41 cattle samples, respectively. In several samples, high proportions of *Treponema* and *Bifidobacterium* were observed (Figure 1B). *Treponema* was the bacterial genus that commonly exists in swine and cattle gut microbiomes. Its prevalence was 46.34% in cattle, while 97.22% in swine.

The genus-level composition was significantly different between swine and cattle (p-value < 0.001, permanova test). Nonmetric multidimensional scaling (NMDS) also showed a distinct separation between swine and cattle (Figure 2A). Since swine and cattle had significantly different compositions, samples from each group were clustered distinctively. In addition, the effect of the farm on the variation of bacterial taxonomic abundance in swine and cattle was significant ($p < 0.001$, permanova test). Notably, the alpha diversity in cattle was lower than that in swine (Supplementary Figure S4). The Shannon diversity for swine was 1.86, while that for cattle was 1.23 on average. Similarly, the inverse Simpson index for swine was 4.91, while that for cattle was 2.96 on average.

Pervasive antibiotic resistance genes in the gut microbiomes of swine and cattle

The abundance of resistomes was investigated by using reads per kilobase and million reads (RPKM) of resistance genes. According to the antibiotic resistance ontology provided by the CARD database [18], the resistance genes identified from the gut microbiomes were assigned to the classes based on the determinant types. It should be noted that efflux pump-related genes were excluded in this study since the homology search of such genes was less accurate, as found in previous studies [19, 20]. In both swine and cattle, the median numbers of RPKM were higher than zero in seven classes: aminoglycoside, beta-lactam, lincosamide, nucleoside, macrolide, tetracycline, and macrolide-lincosamide-streptogramin shared (MLS). This finding implies that the prevalence of each of these seven classes was higher than 50%.

Notably, the abundance of the resistance genes was higher in swine than in cattle. This observation is consistent with the resistant phenotypes. In the antimicrobial susceptibility testing with *Escherichia coli*, frequently observed resistance in both swine and cattle was that against aminoglycosides, sulfonamides, and tetracyclines (Table 1). For cattle, the resistance was observed against only four classes of antibiotics: tetracycline, aminoglycoside, sulfonamide, and quinolone. Moreover, the

resistance rates observed from the antimicrobial susceptibility test with *E.coli* were relatively lower in cattle than in swine (Table 1). In cattle, the resistance rates were 41.5% for tetracyclines, 28.2% for aminoglycosides, 28.2% for sulfonamides, and 12.8% for quinolones. In swine, the resistance rates were 66.7% for tetracyclines, 66.7% for aminoglycosides, 66.7% for sulfonamides, and 33.3% for quinolones. In addition, the resistance was observed against most of the antibiotic types in swine (Table 1). Overall, the prevalence of resistance and the values of MIC_{50} and MIC_{90} values in swine were higher than those in cattle.

Notably, our non-metric multidimensional scaling (NMDS) analysis showed that the samples from each animal were clustered distinctively (Figure 3A). Statistical analysis showed that the distribution of resistance genes was significantly different between swine and cattle (p-value < 0.001, permanova test). The major factors that showed differential abundance were aminoglycoside, tetracycline, lincosamide, beta-lactam, nucleoside, phenicol, and MLS. The abundance of aminoglycoside resistance genes in swine was higher than that in cattle: 427.22 vs 18.40 median RPKM in swine and cattle, respectively (Figure 3). Consistently, the aminoglycoside antibiotic sales were higher in case of swine than in case of cattle (Figure S3). In addition, beta-lactam, lincosamide, MLS, and phenicol resistance genes were more enhanced in swine: 90.29 vs 60.06 RPKM in swine and cattle for beta-lactam; 146.27 vs 111.55 for lincosamide; 82.81 vs 14.96 for MLS; 1.60 vs 0 for phenicol. Notably, the effect of the farm was significant in the variation of resistance gene abundance in swine and cattle ($P<0.001$, permanova test).

In a previous study on cows in U.S. that were fed with corn, the resistance genes of aminoglycoside, MLS, and tetracycline were mainly detected [11]. Zaheer et al. also observed that tetracycline resistance genes were the most abundant class in cattle [21]. In the analysis of ARG abundance in cattle and feedlot environment, Noyes et al. found that tetracycline resistance genes were the most abundant gene families, followed by MLS and aminoglycoside [22]. Wang, C. et al. screened the ARGs in the swine microbiomes and observed that aminoglycoside and tetracycline resistance were the dominant resistance determinants, followed by beta-lactam resistance [23]. The major resistance determinants, such as aminoglycoside and beta-lactam, were also dominant in the human microbiomes, since streptomycin and penicillin were the most prevalent antibiotics that were administered for both humans and animals [24, 25].

Resistance gene families differentially enhanced in the gut microbiomes of swine and cattle

Although aminoglycoside was one of the most abundant resistance determinants in both swine and cattle, major constituents of this class of antibiotics are related to different resistance gene families. Among aminoglycoside acetyltransferase (AAC), aminoglycoside phosphotransferase (APH), and aminoglycoside nucleotidyltransferase (ANT), AAC resistance genes were rarely found in cattle

(Figure 4A). Moreover, there were predominant gene families of ANT and APH in swine and cattle: *ANT(6)* and *APH(3′)*. The prevalence of *ANT(6)*, *ANT(9)*, and *APH(3′)* were 100%, 100%, and 100% in swine, and 97.56%, 100%, and 100% in cattle samples, respectively. Even though *APH(3′)* and *ANT(6)* are the most abundant gene families in cattle, the overall abundance of ARGs in swine is evidently higher than that in cattle: 249.57 vs 1.91 RPKM for *APH(3′)* in swine and cattle; 90.47 vs 3.21 RPKM for *ANT(6)*; 14.97 vs 9.96 RPKM for *ANT(9)*. Most of the ANT, APH, and AAC genes other than *ANT(6), ANT(9),* and *APH(3′)* showed low prevalence in cattle. Notably, *AAC(6′), APH(2″), APH(3″), APH(6),* and *ANT(3″)* were highly prevalent in swine, but not in cattle: 97.22%, 100%, 77.78%, 83.33%, and 94.44% of prevalence for swine; 12.20%, 60.98%, 26.83%, 24.39%, and 24.93% for cattle.

Beta-lactam resistance gene families were not diverse in swine and cattle, compared to aminoglycoside resistance gene families (Figure 4C and D). In both swine and cattle, the most abundant beta-lactam gene family was *CfxA*, which existed mostly in *Bacteroides* and *Prevotella*. *CfxA* was the most abundant in swine, followed by *ACI* and *OXA*. *ACI* and *OXA* were more prevalent in swine than in cattle. The prevalence of *ACI* was 100% and 53.66%, and that of *OXA* was 80.56% and 2.44%, in swine and cattle, respectively. Most of the *OXA* genes in our samples were *OXA-2*, *OXA-61*, and *OXA-335*. Fortunately, extended-spectrum beta-lactamases (ESBL) resistance genes such as *SHV*, *TEM*, and *CTX-M* were rarely found in this study.

Tetracycline was highly prevalent in cattle and swine (Figure 4E). Interestingly, the five most abundant gene families were common in cattle and swine: *tet32*, *tet44*, *tetO*, *tetQ*, and *tetW*. All of the swine and cattle samples contained these resistance genes. The prevalence of these genes was 100%. These five genes were also found as the dominant tetracycline resistance genes in cattle by Zaheer et al. [21]. *Tet32* was one of the most prevalent families in swine; it was also observed in a previous study [26]. *Tet32*, *tetO*, *tetQ*, and *tetW* were identified as the prevalent genes in humans in China, Denmark, and Spain [3, 4]. By means of PCR-based screening, Bryan et al. screened *E. coli* isolates from swine and found higher tetracycline resistance than those from cattle after the analysis of 14 tetracycline resistance genes [27]. In particular, *tetA* and *tetB* were found as dominant genes in swine and cattle.

Homologous resistance genes found across different farm animals

ANT(6) was the most prevalent genes in both swine and cattle; it was found in all samples, except one sample of cattle (Figure 4A). For the network analysis on the prevalent aminoglycoside resistance genes, *ANT(6)* genes were also identified from the bacterial genomes in the NCBI repository to reveal the bacterial source of ARGs in our samples. The *ANT(6)* genes found in the samples were homologous with three known genes: *ANT(6)-Ia* of *Exiguobacterium*, *ANT(6)-Ib* of *Campylobacter*,

and *aad(6)* of *Streptococcus* (Figure 5A; in orange color). In particular, a large amount of the resistance genes assembled from the samples were mainly associated with *ANT(6)-Ib* and *aad(6)*. In the graphs shown in Figure 5, the genes that share higher than 100% sequence similarity were connected as a cluster and the clusters that share higher than 98% sequence similarity were grouped in the dotted circle. For the *ANT(6)* gene family, *ANT(6)-Ib* genes are common in both swine and cattle (Figure 5A). The *ANT(6)-Ib* genes were also clustered together with the genes in *Clostridioides difficile* and *Campylobacter fetus* (Figure 5A). Interestingly, there was a cluster (iv) (see Figure 5A) of genes, which shared about 73% similarity to the protein sequence of *ANT(6)-Ib*. This cluster might be a gene family that is prevalent in Korean swine and cattle. In our study, this particular gene was found in all cattle samples. Notably, *aad(6)* gene was also found in all the cattle samples; it mostly originates from *Staphylococcus* and *Enterococcus* species.

APH(3') was also prevalent in both swine and cattle (Figure 5B). Two genes, *APH(3')-Ia* and *APH(3')-IIIa,* were enhanced in our samples. Most of the *APH(3')-Ia* genes were found in swine; *APH(3')-IIIa* was found in both swine and cattle, but its abundance was much higher in swine (Figure 5B). The *APH(3')-Ia* genes were found in *Salmonella*, *Escherichia*, *Corynebacterium*, and *Serratia* species. The 14 *APH(3')-Ia* genes in the swine were the same as those in *Salmonella*. The *APH(3')-IIIa* genes in swine and cattle were identical to those in *Enterococcus* and *Streptococcus* species.

For tetracycline, there were two most abundant and prevalent genes: *tetQ* and *tetW* (Figure 5C and D). In the search with the *tetQ* gene, two homologous clusters were built. The ARGs in the bigger cluster (Figure 5C(i)) showed 100% sequence similarity with the genes in *Bacteroides* and *Prevotella*. The *tetW* gene family consisted of more diverse gene clusters. The biggest cluster that contains genes from swine and cattle were similar with the annotated genes of *Bifidobacterium* in the CARD database (Figure 5D(i)). The other clusters showed lower sequence similarity against the known genes, which was about 93% for cluster (ii), 88% for cluster (iii), and 78% for cluster (iv). For more accurate annotation, they need to be validated by antibiotics susceptibility testing.

DISCUSSION

Farm animals such as swine and cattle are usually treated with antibiotics to prevent infectious diseases and to promote their growth [5]. Moreover, manure or wastewater from animal farms contains more abundant resistance genes than other environments such as soil and rivers [26]. A large-scale study on the prevalence and diversity of antibiotic resistance genes in the farm animals should help better understand the current situation of antibiotic resistance prevalence, and accordingly develop public health policies. In this work, we comprehensively investigated resistomes in the farm animals such as swine and cattle using a set of unbiased shotgun sequencing data. From a total of 36

swine in 12 farms and 41 cattle in 13 farms, gut microbiomes were collected nationwide and sequenced to identify resistance genes.

Swine are administered more antibiotics because of their dense breeding environment and higher exposure rate to bacterial diseases. Data on the antibiotics sales in Korea showed that approximately 510 tons of antibiotics for swine every year between 2017 and 2018, whereas 88 tons for cattle (Supplementary Figure S3). The sales rates for beta-lactams, tetracyclines, aminoglycosides, sulfonamides, macrolides, and phenicols were particularly high, which is consistent with the abundance of resistance gene determinants that we identified in the swine and cattle gut microbiomes. In summary, our study has revealed a general association between the antibiotics usage, resistance phenotype, and resistance genes in the host gut microbiomes (Supplementary Figure S5). Overall, antibiotics sales, resistance rates from the susceptibility tests, and the abundance of resistance genes in the gut microbiomes were higher in swine than in cattle. Aminoglycoside, beta-lactam, tetracycline, and MLS are the abundant resistance classes in terms of antibiotic sales, susceptibility testing, and genes accumulated in the gut microbiomes.

The sequence homology of resistance genes was investigated to determine the possibility of gene transfer between swine and cattle. Among the aminoglycoside resistance genes, two gene families, *APH(3')* and *ANT(6)*, were prevalent in both swine and cattle. *APH(3')*, however, showed strong conservation separately in swine and cattle. Specifically, the *APH(3')-la* gene was dominant in swine, whereas the *APH(3')-llla* gene was in both cattle and swine. On the other hand, *ANT(6)* showed different patterns. The *ANT(6)-lb* gene, identified originally in *Campylobacter*, was found in both swine and cattle.

The most prevalent and abundant tetracycline genes that we found in this study were *tetQ*, *tetQ*, *tet32*, *tet44*, and *tetW*. *Tet32* was one of the most prevalent families in swine in a previous study [26]. *Tet32*, *tetO*, *tetQ*, and *tetW* were identified as the most prevalent genes in humans in China, Denmark, and Spain [3, 4]. These genes were also observed abundantly in the manure, but not in regular soil [5]. This observation might suggest that they are human- and animal-related resistance genes.

In Korea, penicillins, tetracyclines, and aminoglycosides are the three most frequently used antibiotics for cattle. For swine, penicillins, phenicols, and tetracyclines are the three most highly used antibiotics. While the abundant resistance determinants found in our study were related to these antibiotics, the association with the amount of usage of these antibiotics was not strong. For example, phenicol resistance genes were not abundant in our samples, although phenicol is one of the most frequently administered antibiotics. A similar discrepancy was also observed in a previous study [5]. However,

the observation that phenicol resistance genes were observed in swine, but not in cattle, is consistent with the antibiotics usage trend in Korea.

POTENTIAL IMPLICATIONS

From the gut microbiomes of 36 swine and 41 cattle, large-scale metagenomic analysis was performed to find the prevalence and diversity of ARGs in two different types of farm animals in Korea. This genome-level investigation of ARGs in the multiple farm animals should provide valuable information to better understand horizontal and vertical transfer of ARGs in farm animals. In particular, the investigation of tetracycline ARGs identified in the microbiomes showed that identical *tetQ* and *tetX* genes were found both in swine and cattle, while several types of ARG sequences were quite different between the two animals. This observation establishes the presence of two different patterns of resistance genes: one type is host-dedicated, and the other is prevalent in different hosts. An in-depth study of resistomes should also help analyze how antibiotic resistance genes spread among livestock, environments, and human microbiomes.

METHODS

Sample Collection

A total of 41 fecal samples of cattle were collected from 13 farms located in 6 provinces. For each farm, three or four samples were collected from different animals to compare the diversity depending on the farming environment. Of the 13 farms, three had < 50 heads, two had 50–100 heads, and eight had > 100 heads. The age of cattle ranged from 19 to 90 months (average 34 months). In each farm, five cattle were randomly chosen. From these five samples, three samples with different antibiotic resistance patterns of *E. coli* were selected (Supplementary Table S2).

A total of 36 fecal samples were collected from 12 swine farms located in 6 provinces. For each farm, three samples were collected from different animals to compare the diversity depending on the farming environment. Of the 12 farms, one farm had < 1,000 heads, nine had 1,000–5,000 heads, one had 5000–10,000, and one had > 10,000 heads. The age of the swine ranged from 150 to 230 days. In each farm, five swine were randomly chosen. From these five samples, three samples with different antibiotic resistance patterns of *E. coli* isolated were selected (Supplementary Table S3).

DNA preparation

The samples were immediately transported to the laboratory in ice-cooled containers and stored at –70˚C until DNA extraction was performed. Each sample was thoroughly mixed using a spatula and

divided into 250–300 mg aliquots. The total DNA was extracted using the Fast DNA SPIN Kit for Feces (MP Biomedicals, #116570200) following the manufacturer's instructions. DNA purity and concentration were evaluated by measuring the absorbances (ABS) at 260 nm and 280 nm using a NanoDropTM spectrophotomer (NanoDropTM 2000, Thermo Fisher Scientific Inc, Wilmington, DE, USA). All the DNA samples had ABS_{260}/ABS_{280} ratios of 1.8–2.0. Illumina HiSeq4000 Platform (Illumina, San Diego, USA) was used to sequence the DNA samples. We used the TruSeq DNA PCR Free Kit (Illumina, San Diego, USA) and did not include PCR amplification step.

Sequencing and read filtering

A total of 77 gut microbiomes were sequenced from swine and cattle for this study. For every sample, 151-bp paired-end sequences were generated from the insert of 350 bps. An average of 38 M pairs of reads (ranging between 25 M and 75 M) were generated from each sample after filtering. Low-quality reads were removed using Sickle [28] with the threshold of Phred quality score > 20 and read length > 90 bps (pe -q 20 -t sanger -l 90). Reads containing "N" were also removed. Finally, host contamination was removed by discarding the reads that were mapped to the swine and cattle genomes provided by NCBI. For this process, bowtie2-align version 2.1.0 was used with the sensitive-local option. The swine reference genome used was Swine –Sscrofa11.1 (GCF_000003025.6). The cattle reference genome used was Cattle - Bos_taurus_UMD_3.1.1 (GCF_000003055.6).

Antimicrobial susceptibility testing

Samples were processed, and *E. coli* was isolated as described previously [29] using eosin methylene blue agar (Becton Dickinson, Sparks, MD 21152, USA) and MacConkey agar plates (BD). Species identification was performed using matrix-assisted laser desorption ionization time-of-flight mass spectrometry (bioMérieux, Marcy l'Étoile, France).

Antimicrobial susceptibility was assessed by determining the minimum inhibitory concentrations (MICs) for 16 antimicrobial agents using the broth microdilution method with a commercially available Sensititre® panel KRVP4F (TREK Diagnostic Systems, West Sussex, UK) according to the manufacturer's instructions. The following antibiotics were tested: ampicillin, amoxicillin/clavulanic acid, cefoxitin, ceftiofur, ceftazidime, cefepime, chloramphenicol, ciprofloxacin, colistin, gentamicin, meropenem, nalidixic acid, streptomycin, sulfisoxazole, tetracycline, and trimethoprim/sulfamethoxazole. The reference strain *E. coli* ATCC 25922 was used as quality control when determining MICs. The MIC was interpreted according to the Clinical and Laboratory Standards Institute (CLSI) guidelines (CLSI, 2017). When CLSI breakpoints were not available, the MIC was interpreted according to the Danish Integrated Antimicrobial Resistance Monitoring and

Research Programme (DANMAP, 2014). Multidrug resistance was defined as resistance to three or more antibiotic subclasses.

Profiling and statistical analysis of bacterial composition

After the filtering of sequencing reads, bacterial composition was profiled using MetaPhlAn [30] with the default option. For homology search in the MetaPhlAn process, bowtie2 with the very-sensitive option was used. To compare the bacterial composition of swine and cattle, alpha and beta diversity was measured [31, 32] and plotted using the vegan package (ver. 2.5-6). For alpha diversity, Shannon index and Simpson index were calculated. From each sample, 25 M reads were sampled and its taxonomy composition was determined by using MetaPhlAn. The genus proportion was used for calculating Shannon index and Simpson index. Two different thresholds were applied to include genera in the calculation: i) > 0.1% in any of the samples, and ii) without any proportion threshold. For beta diversity, nonmetric multidimensional scaling was performed with Bray-Curtis distance. The metaMDS() function in the vegan package was used. The differential abundance of bacterial taxon between swine and cattle was determined using t-test (scipy.stats.ttest_ind() in scipy package). To estimate the correlation between bacterial genera, SPARCC was performed on the composition data. To determine the effects of feedlots on bacterial composition, permutational multivariate analysis of variance (permanova) was performed using the *adonis* function of the vegan package.

Profiling the abundance of antibiotic resistance genes

The abundance of ARGs was measured by RPKM, as performed in a previous study [33]. CARD version 2.0.1 was used to create a file consisting of 848 representative ARG sequences after clustering homologous sequences using cd-hit (-c 0.9 -n 8). Minor changes were added to the database file. *AAC(6′)-Ib’* and *OXA-368* sequences from CARD v.3.0.7 were used to update the database file. *BLA1* from class A was excluded since it was clustered with *BcI* from class B, and genes clustered with the *ANT(3″)-li-AAC(6′)-IId* fusion protein were all excluded and handled separately in the post-processing step. Sample reads were aligned to the representative sequences using bowtie2 (--sensitive-local). The reads aligned were retained if the aligned length was longer than 50% of the ARGs, and their similarity was greater than 70%. By using these quality-controlled reads, RPKM was calculated as follows:

$$\mathrm{RPKM} = \frac{\text{Number of reads mapped to reference} \times 10^9}{\text{Number of reads in sample} \times \text{Reference length}}$$

Finally, the genes were considered to exist when 70% of the ARG length was covered by the reads.

Identification of complete antibiotic resistance genes and network analysis

To identify complete antibiotic resistance genes that contain start and stop codons, a three-step procedure was performed. First, filtered reads were assembled into contigs using MEGAHIT [34] with default options using only paired-end reads. Only contigs of length > 500 bp were used for gene prediction. In addition, we further expanded the assembly with the reads collected with three different conditions to improve the assembly: (i) all reads that were mapped to the sequences in the ARG database; (ii) a subset with 5% of reads randomly selected; (iii) reads mapped to the genes of high depth (>100). Since different approaches might generate redundant genes in a sample, clustering was applied using cd-hit-est (-c 1 -n 8) on the predicted genes to create a set of non-redundant ARGs in each sample. Secondly, to predict genes from contigs, FragGeneScan [35] was applied with the options of no sequencing errors (-w 1 –t complete). Lastly, genes predicted in the metagenomic data set were aligned with the antibiotic resistance genes annotated in the CARD database [18]. CARD version 2.0.1 includes a total of 2,252 protein sequences. Antibiotic resistance genes from uncultured bacteria and the genes annotated as regulatory system- or efflux pump-related were excluded. The resistance genes were classified into 21 ARG classes based on the gene ontology [18]. We added MLS classes (lincosamide, macrolide, and streptogramin shared) that have *Cfr 23S* and *Erm 23S* as subclasses, because these two subclasses are commonly found in the three classes. Blastp [36] was used for ARG annotation with an e-value threshold of 1×10^{-10}, similarity exceeding 70%, and reference coverage over 70%.

With the ARGs identified from the samples, network analysis was performed with the annotated genes. To collect the annotated genes, all the genes of the 8,369 complete genomes downloaded from the NCBI repository (https://www.ncbi.nlm.nih.gov/) were searched against the ARGs database. A network graph was built with the nodes of ARGs using cytoscape (https://cytoscape.org). Colors of the nodes represent either the sample or host genus. The nodes were connected with a full line, if two ARG sequences are same (similarity of 100% with 100% coverage). The clusters were further grouped by the dotted circle if the clusters share 98% or higher similarity with any of the clusters.

Table 1. Antibiotic resistance of *Escherichia coli* (*n* = 77) isolated from animal fecal samples

Antimicrobial subclass	Antimicrobial agents	Breakpoint (µg/ml)	swine (n=36)			Cattle (n=41)		
			MIC_{50} (µg/ml)	MIC_{90} (µg/ml)	Resistance % (no.)	MIC_{50} (µg/ml)	MIC_{90} (µg/ml)	Resistance % (no.)
Aminoglycosides	Gentamicin (GEN)	≥16	1	32	27.8(10)	1	1	0(0)
	Streptomycin (STR)	≥32	64	128	66.7(24)	16	64	26.8(11)
Aminopenicillin	Ampicillin (AMP)	≥32	64	64	69.4(25)	4	4	0(0)
β-lactam/-lactamase inhibitor combinations	Amoxicillin/ clavulanic acid (AmC)	≥32/16	8	8	0(0)	2	4	0(0)
Cephamycin	Cefoxitin (FOX)	≥32	4	8	0(0)	4	4	0(0)
Cephalosporin III	Ceftiofur (XNL)	≥8	0.5	0.5	2.8(1)	0.5	0.5	0(0)
	Ceftazidime (CAZ)	≥16	1	1	0(0)	1	1	0(0)
Cephalosporin IV	Cefepime (FEP)	≥16	0.25	0.25	0(0)	0.25	0.25	0(0)
Carbapenem	Meropenem (MEM)	≥4	0.25	0.25	0(0)	0.25	0.25	0(0)
Fluoroquinolone	Ciprofloxacin (CIP)	≥4	0.25	8	16.7(6)	0.12	0.25	0(0)
Folate pathway inhibitors	Trimethoprim/ Sulfamethoxazole (SXT)	≥4/76	0.25	4	33.3(12)	0.12	0.12	0(0)
Sulfonamides	Sulfisoxazole (FIS)	≥512	512	512	66.7(24)	32	512	26.8(11)
Phenicols	Chloramphenicol (CHL)	≥32	64	64	66.7(24)	8	8	0(0)
Polymyxins	Colistin (COL)	≥4	2	2	0(0)	2	2	0(0)
Quinolone	Nalidixic acid (NAL)	≥32	8	128	33.3(12)	2	64	12.2(5)
Tetracyclines	Tetracycline (TET)	≥16	64	128	66.7(24)	2	128	41.5(17)

MIC_{50} and MIC_{90} are the concentrations at which 50% and 90% of the isolates, respectively, were inhibited.

Availability of supporting data and materials

Table S1. Sequencing data information for swine and cattle gut microbiomes

Table S2. Sample information for cattle

Table S3. Sample information for swine

Other data further supporting this work are openly available in the GigaScience repository, GigaDB [37].

Declaration

List of abbreviations

ARG: Antibiotic resistance gene; PCA: Principle component analysis; GPM: Genes per million genes predicted; MLS: macrolide-lincosamide-streptogramin shared; APH: Aminoglycoside phosphotransferase; ANT: aminoglycoside nucleotidyltransferase; AAC: aminoglycoside acetyltransferase

Consent for publication

Not applicable.

Competing interests

The authors declare that they have no competing interests.

Funding

This research was supported by a grant (2017NER54070) from Research of Korea Centers for Disease Control and Prevention to SL and MR.

Authors' contributions

SL and MR conceived and designed the study. SL performed the experiments and analysis. DK, YC, and MR performed the analysis. DM prepared the samples and performed experiments. SL, DK, DM, YC, and MR wrote the manuscript. All authors read and approved the final manuscript.

Acknowledgements

We thank Drs. Hyunjoo Pai, Jieun Kim, Chang-Jun Cha, and Inho Park for their helpful discussions.

References

1. Allen, H.K., et al., *Call of the wild: antibiotic resistance genes in natural environments.* Nat Rev Microbiol, 2010. **8**(4): p. 251-9.
2. Smillie, C.S., et al., *Ecology drives a global network of gene exchange connecting the human microbiome.* Nature, 2011. **480**(7376): p. 241-4.
3. Forslund, K., et al., *Country-specific antibiotic use practices impact the human gut resistome.* Genome Res, 2013. **23**(7): p. 1163-9.
4. Hu, Y.F., et al., *Metagenome-wide analysis of antibiotic resistance genes in a large cohort of human gut microbiota.* Nature Communications, 2013. **4**.
5. Zhu, Y.G., et al., *Diverse and abundant antibiotic resistance genes in Chinese swine farms.* Proc Natl Acad Sci U S A, 2013. **110**(9): p. 3435-40.
6. Pal, C., et al., *The structure and diversity of human, animal and environmental resistomes.* Microbiome, 2016. **4**(1): p. 54.
7. Sommer, M.O.A., G. Dantas, and G.M. Church, *Functional Characterization of the Antibiotic Resistance Reservoir in the Human Microflora.* Science, 2009. **325**(5944): p. 1128-1131.
8. Forsberg, K.J., et al., *The shared antibiotic resistome of soil bacteria and human pathogens.* Science, 2012. **337**(6098): p. 1107-11.
9. Wichmann, F., et al., *Diverse antibiotic resistance genes in dairy cow manure.* MBio, 2014. **5**(2): p. e01017.
10. Xiao, L., et al., *A reference gene catalogue of the pig gut microbiome.* Nature Microbiology, 2016. **1**: p. 16161.
11. Noyes, N.R., et al., *Characterization of the resistome in manure, soil and wastewater from dairy and beef production systems.* Sci Rep, 2016. **6**: p. 24645.
12. Shoemaker, N.B., et al., *Evidence for extensive resistance gene transfer among Bacteroides spp. and among Bacteroides and other genera in the human colon.* Appl Environ Microbiol, 2001. **67**(2): p. 561-8.
13. Gerzova, L., et al., *Characterization of Antibiotic Resistance Gene Abundance and Microbiota Composition in Feces of Organic and Conventional Pigs from Four EU Countries.* PLoS One, 2015. **10**(7): p. e0132892.
14. Quan, J., et al., *A global comparison of the microbiome compositions of three gut locations in commercial pigs with extreme feed conversion ratios.* Sci Rep, 2018. **8**(1): p. 4536.
15. Rice, W.C., et al., *Influence of wet distillers grains diets on beef cattle fecal bacterial community structure.* BMC Microbiol, 2012. **12**: p. 25.
16. Shanks, O.C., et al., *Community structures of fecal bacteria in cattle from different animal feeding operations.* Appl Environ Microbiol, 2011. **77**(9): p. 2992-3001.
17. Human Microbiome Project, C., *Structure, function and diversity of the healthy human microbiome.* Nature, 2012. **486**(7402): p. 207-14.
18. McArthur, A.G., et al., *The comprehensive antibiotic resistance database.* Antimicrob Agents Chemother, 2013. **57**(7): p. 3348-57.
19. Ueda, O., et al., *Sixteen homologs of the mex-type multidrug resistance efflux pump in Bacteroides fragilis.* Antimicrob Agents Chemother, 2005. **49**(7): p. 2807-15.
20. Kroeger, J.K., et al., *Bacillus cereus e ffl ux protein BC3310 - a multidrug transporter of the unknown major facilitator family, UMF-2.* Front Microbiol, 2015. **6**: p. 1063.
21. Zaheer, R., et al., *Comparative diversity of microbiomes and Resistomes in beef feedlots, downstream environments and urban sewage influent.* BMC Microbiol, 2019. **19**(1): p. 197.

22. Noyes, N.R., et al., *Resistome diversity in cattle and the environment decreases during beef production.* Elife, 2016. **5**: p. e13195.
23. Wang, C., et al., *Characterization of the Pig Gut Microbiome and Antibiotic Resistome in Industrialized Feedlots in China.* mSystems, 2019. **4**(6).
24. Elander, R.P., *Industrial production of beta-lactam antibiotics.* Appl Microbiol Biotechnol, 2003. **61**(5-6): p. 385-92.
25. Mingeot-Leclercq, M.P., Y. Glupczynski, and P.M. Tulkens, *Aminoglycosides: activity and resistance.* Antimicrob Agents Chemother, 1999. **43**(4): p. 727-37.
26. Li, B., et al., *Metagenomic and network analysis reveal wide distribution and co-occurrence of environmental antibiotic resistance genes.* ISME J, 2015. **9**(11): p. 2490-502.
27. Bryan, A., N. Shapir, and M.J. Sadowsky, *Frequency and distribution of tetracycline resistance genes in genetically diverse, nonselected, and nonclinical Escherichia coli strains isolated from diverse human and animal sources.* Appl Environ Microbiol, 2004. **70**(4): p. 2503-7.
28. Joshi NA, F.J., *Sickle: A sliding-window, adaptive, quality-based trimming tool for FastQ files (Version 1.33) [Software]. Available at* https://github.com/najoshi/sickle. 2011.
29. Nam, H.M., et al., *Prevalence of antimicrobial resistance in fecal Escherichia coli isolates from stray pet dogs and hospitalized pet dogs in Korea.* Microb Drug Resist, 2010. **16**(1): p. 75-9.
30. Truong, D.T., et al., *MetaPhlAn2 for enhanced metagenomic taxonomic profiling.* Nat Methods, 2015. **12**(10): p. 902-3.
31. Gloor, G.B., et al., *Microbiome Datasets Are Compositional: And This Is Not Optional.* Front Microbiol, 2017. **8**: p. 2224.
32. Calle, M.L., *Statistical Analysis of Metagenomics Data.* Genomics Inform, 2019. **17**(1): p. e6.
33. Munk, P., et al., *Abundance and diversity of the faecal resistome in slaughter pigs and broilers in nine European countries.* Nat Microbiol, 2018. **3**(8): p. 898-908.
34. Li, D., et al., *MEGAHIT: an ultra-fast single-node solution for large and complex metagenomics assembly via succinct de Bruijn graph.* Bioinformatics, 2015. **31**(10): p. 1674-6.
35. Rho, M., H. Tang, and Y. Ye, *FragGeneScan: predicting genes in short and error-prone reads.* Nucleic Acids Res, 2010. **38**(20): p. e191.
36. Altschul, S.F., et al., *Basic local alignment search tool.* J Mol Biol, 1990. **215**(3): p. 403-10.
37. Lim S; Kim D; Moon D; Cho Y; Rho M: Supporting data for "Antibiotic resistomes discovered in the gut microbiomes of Korean swine and cattle" GigaScience Database. 2020. http://dx.doi.org/10.5524/100730

Figure Legend

Figure 1. Bacterial composition of swine and cattle gut microbiomes. Genus-level bacterial composition in (A) swine and (B) cattle. The ten most abundant bacterial phyla in (C) swine and (D) cattle. The ten most abundant bacterial genera in (E) swine and (F) cattle.

Figure 2. Different bacterial compositions in the gut microbiomes of swine and cattle. (A) Nonmetric multidimensional scaling analysis of genus-level bacterial composition in swine and cattle. (B) Differential distribution of genus composition in swine and cattle (p-value < 0.01; median relative abundance in any sample > 1%).

Figure 3. Composition of antibiotic resistance genes in swine and cattle. (A) Nonmetric multidimensional scaling analysis with the abundance of antibiotic resistance genes in swine and cattle. (B) Distribution of significant antibiotic resistance determinants in swine and cattle (p-value < 0.01).

Figure 4. Antibiotic resistance genes in swine and cattle gut microbiomes. Binary heatmap showing the presence of resistance genes for (A) aminoglycosides, (C) beta-lactams, and (E) tetracyclines. The five most abundant gene families of antibiotic resistance genes for (B) aminoglycosides, (D) beta-lactams, and (F) tetracyclines. The y-axis represents log-transformed RPKM.

Figure 5. Network analysis of resistance genes and their similarity in swine and cattle gut microbiomes. Network of (A) ANT(6), (B) APH(3′), (C) tetQ, and (D) tetW. The nodes in the network are resistance genes identified in swine (blue) and cattle (red), stored in the CARD database (orange) and identified from the bacterial complete genomes (yellow). The solid lines connecting nodes represent 100% similarity between two ARG sequences. The same ARG sequences from the samples were connected as a cluster. The clusters in each dotted circle show 98% or higher similarity.

Figure

Click here to access/download;Figure;AR_Farm_Animals_Figure_0229.pptx

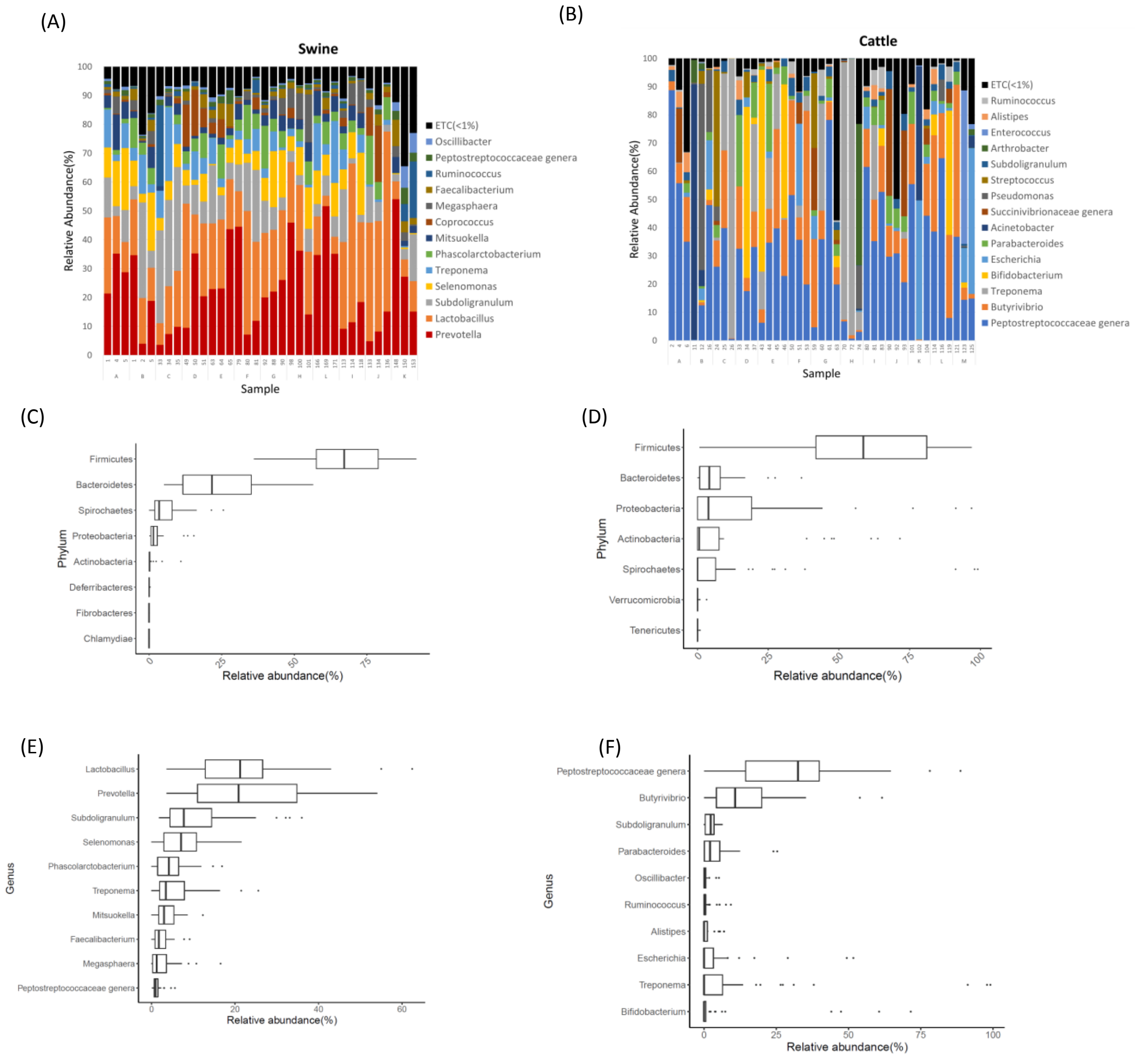

Figure 1. Bacterial composition of swine and cattle gut microbiomes. Genus-level bacterial composition in (A) swine and (B) cattle. The ten most abundant bacterial phyla in (C) swine and (D) cattle. The ten most abundant bacterial genera in (E) swine and (F) cattle.

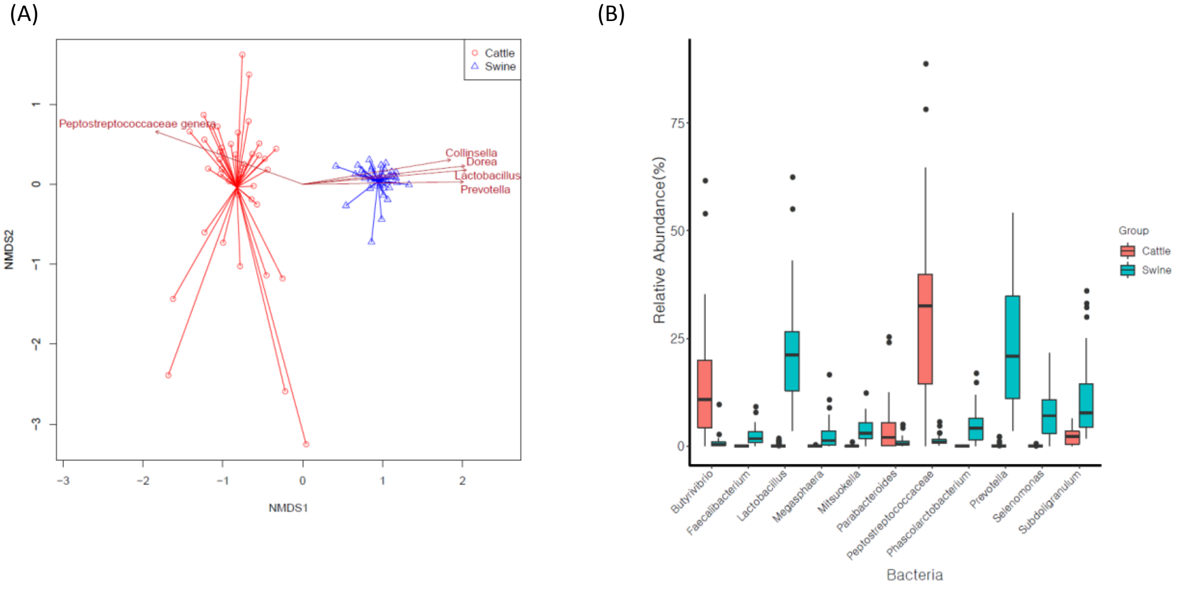

Figure 2. Different bacterial compositions in the gut microbiomes of swine and cattle. (A) Nonmetric multidimensional scaling analysis of genus-level bacterial composition in swine and cattle. (B) Differential distribution of genus composition in swine and cattle (p-value < 0.01; the median relative abundance in any sample > 1%).

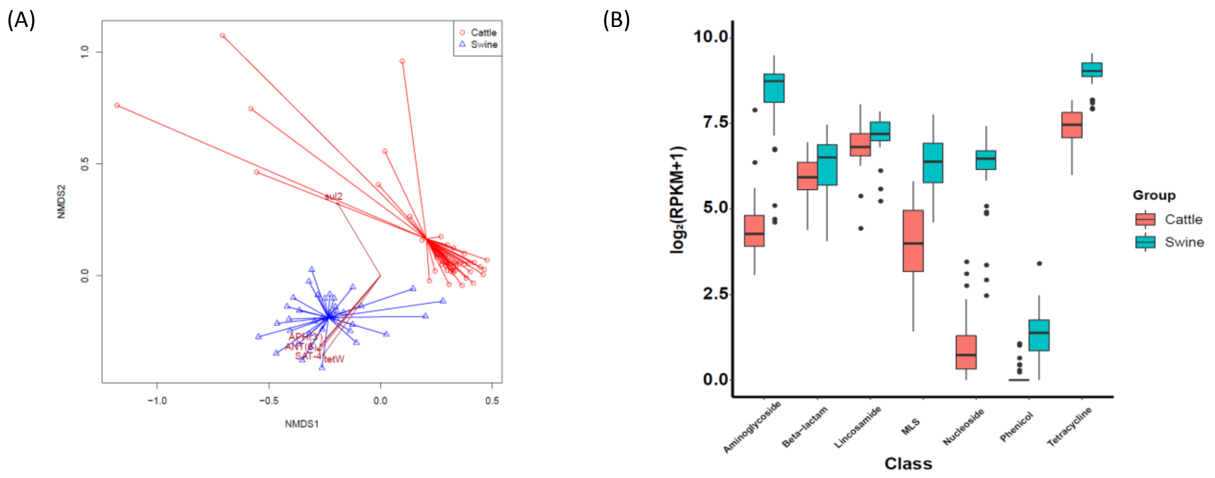

Figure 3. Composition of antibiotic resistance genes in swine and cattle. (A) Nonmetric multidimensional scaling analysis with the abundance of antibiotic resistance genes in swine and cattle. (B) The distribution of significant antibiotic resistance determinants in swine and cattle (p-value < 0.01).

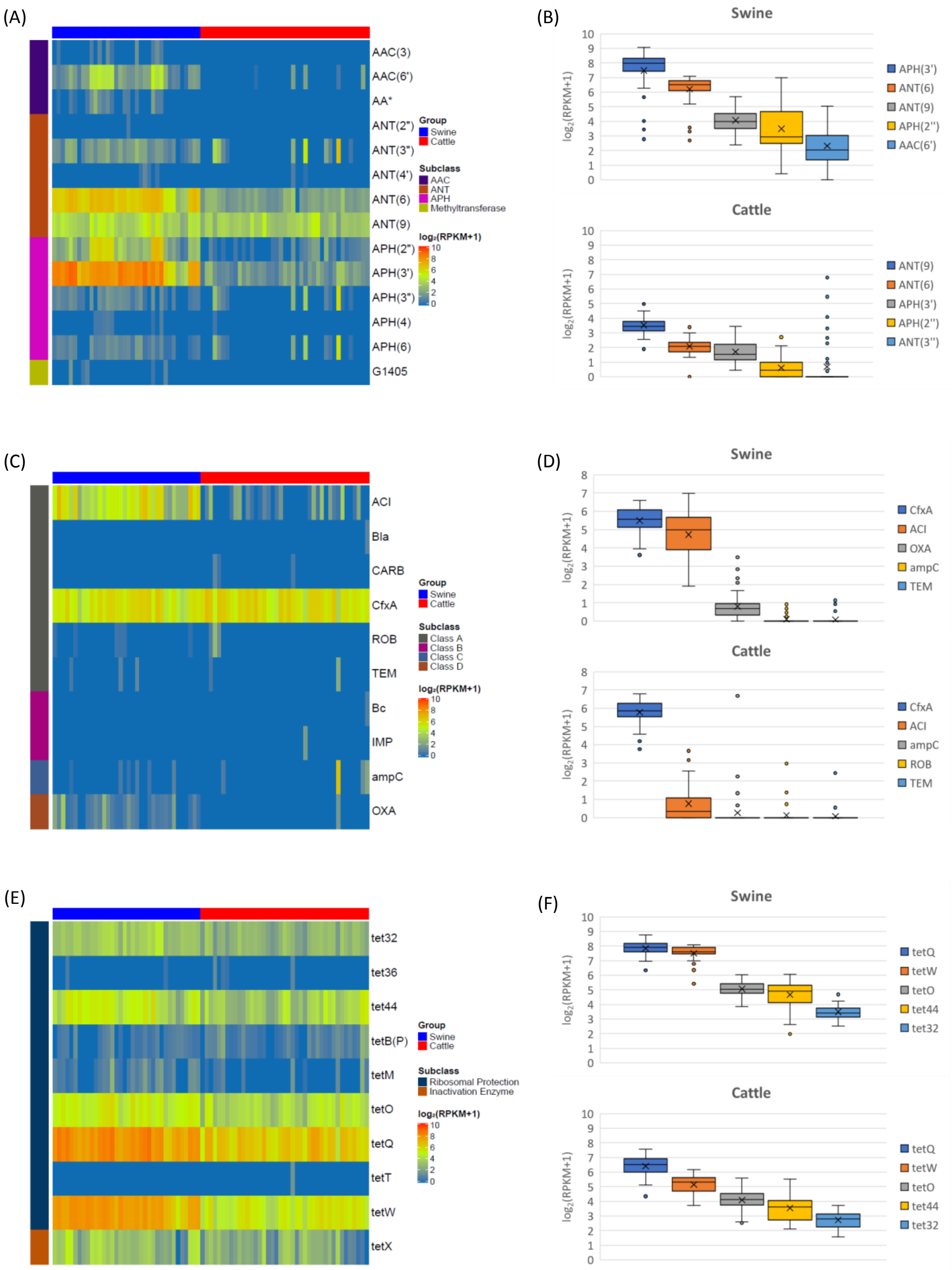

Figure 4. Antibiotic resistance genes in swine and cattle gut microbiomes. Binary heatmap showing the presence of the resistance genes for (A) aminoglycosides, (C) beta-lactams, and (E) tetracyclines. The five most abundant gene families of antibiotic resistance genes for (B) aminoglycoside, (D) beta-lactam, and (F) tetracycline. The y-axis represents log-transformed RPKM values.

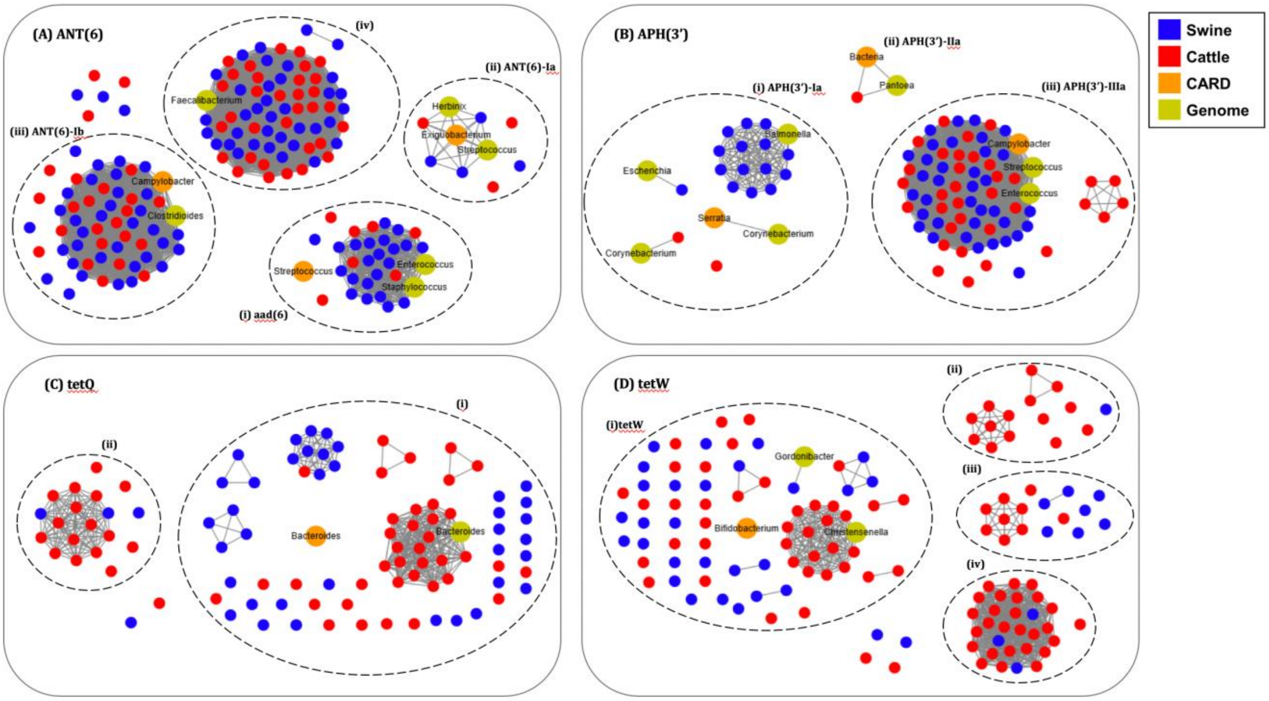

Figure 5. Network analysis of resistance genes and their similarity in the swine and cattle gut microbiomes. Network of (A) ANT(6), (B) APH(3'), (C) tetQ, and (D) tetW. The nodes in the network are resistance genes identified in swine (blue) and cattle (red), stored in CARD database (orange) and identified from the bacterial complete genomes (yellow). The solid lines connecting nodes represent 100% similarity between two ARG sequences. The same ARG sequences from the samples were connected as a cluster. The clusters in each dotted circle show 98% or more similarity.

Click here to access/download
Supplementary Material
AR_Farm_Animals_Supplementary_Table_0229.docx

Click here to access/download
Supplementary Material
AR_Farm_Animals_Supplementary_Figure_0316.pptx

Personal Cover Click here to access/download;Personal Cover;Reviewer_response_0316.docx

Mina Rho, Associate Professor
Department of Computer Science and Engineering
Hanyang University
222 Wangsimni-ro, Seongdong-gu
Seoul, Republic of Korea
Phone: (82)-2-2220-2379
minarho@hanyang.ac.kr

March 21th, 2020

Executive Editor
Giga Science

Re: **GIGA-D-19-00340, "Antibiotic resistomes discovered in the gut microbiomes of swine and cattle"**

Dear Editor,

Enclosed please find the revised version of the above titled manuscript that we are submitting for your consideration for publication in *Giga Science*. We very much appreciate the helpful comments from the second reviewer. Listed below are our responses.

We believe that these changes have satisfactorily addressed all the points raised by the reviewers, and greatly improved the manuscript. We hope that this work can be considered for publication in *Giga Science*.

Thank you for your efforts and best wishes.

Sincerely,
Mina Rho

Reviewer #2: I am happy to find, that the authors have more or less addressed all my concerns. I have comments for some of their rebuttals and for some of the changes to the manuscript. Also, the authors / journal might want to specify in the paper title reflect that the scope is Korea, like the authors have now done throughout the manuscript.

Answer to author rebuttal:

1 Looks like a fine approach. What was the difference between the first and second filtering threshold? Could a single "70% pass" give the same result?

Answer) When we compared the number of reads in four samples with and without 70% similarity threshold, there was no difference.

3 I am not going to insist the authors use CODA methods, but as I explained further down, I have some specific objections to their (old and new) analysis, where I don't think it makes sense and could be helped using alternatives suggested by Gloor et al. Also as far as I can see, the transition to CLR is not meaningful when coupled with their chosen analysis.

Answer) Following the reviewer's helpful suggestion, we have performed the correlation analysis with SPARCC. The revised manuscript reports the results in line 396 and lines 112–113, as well as Supplementary Figure S1.

Specific comments:

L31: Language could be a bit more precise in conclusion and throughout. Does "more diverse" refer to a higher alpha diversity / evenness in samples from one livestock species or to a larger beta-diversity within one livestock species compared to the other?

Answer) We used the expression "more diverse" to explain that more genes are prevalent in swine than in cattle. Specifically, for the prevalent ARGs (prevalence > 50%), we found 32 genes in swine, while 19 genes in cattle. We revised the sentence to read "Antibiotic resistome was more pervasive in swine than in cattle. Specifically, prevalent ARGs (prevalence > 50%) were found more in swine than in cattle".

L92: On my request, the authors have now added the information on the actual sequencing instrument (HiSeq4000) which ENA also states. However, the MS also still refers to the HiSeqX. It also still says minimum was 110M PE reads. These discrepancies should be fixed.

Answer) Corrections were made accordingly.

L112: A negative correlation coefficient is given. Is it significant? Prevotella was very common, so it is not surprising there are lower proportions of other bacteria in samples with a lot of Prevotella. See further down, why the test probably does not yield meaningful results.

Answer) In response to the reviewer's suggestion, we performed the correlation analysis with SPARCC. We note that the proportion of *Prevotella* still showed a negative correlation with that of *Subdoligranulum* ($r = –0.6457$).

L169: Were the values normally distributed so the results of t-tests are valid and meaningful? If median cattle phenicol AMR was 0 RPKM, then it seems unlikely? If the authors log transform data (after zero replacement), they will get ALR values which are more likely to be normal.

Answer) The normality test showed that several ARGs including phenicol are not normally distributed. We thus removed the t-test results and just compared RPKM values for abundance between swine and cattle.

L382 "The genus proportion (>0.1%) was used for calculating Shannon index…". Is it correctly understood that rare genera were excluded? That will impact the diversity indexes and seems like it should be justified. I am not convinced its required, especially if the sample was also rarefied (a practice there is some controversy around: https://doi.org/10.1371/journal.pcbi.1003531).

Answer) In response to the reviewer's suggestion, we also calculated the index with the genus without any proportion threshold (Supplementary Figure S4D–F). The result showed the same pattern. The method section was also revised; please see lines 390–392.

L388: If the authors use rank-based correlation (Spearman), CLR transformation will not have any effect. If the authors want to implement some of the CODA principles from the papers, this is not a good place, when coupled with a rank-based correlation. Rank-based does not solve the issue of negative correlation bias. In the Gloor paper I linked (GB Gloor et al, 2017), they say e.g. the following:

"There are several more rigorous approaches that can be applied to analyze correlation in microbiome datasets, including SPARCC (Friedman and Alm, 2012) and SPieCeasi (Kurtz et al., 2015), both of which assume a sparse data matrix, and the ϕ (Lovell et al., 2015) and ρ…"

Answer) In response to the reviewer's suggestion, we performed correlation analysis using SPARCC and revised the manuscript. Please see line 396 and lines 112–113, as well as Supplementary Figure S1.

Supplementary:

Some of the captions are very short and uninformative.
Answer) Corrections were made accordingly.

The authors claim they do not have estimates of the size of Korean pig and cattle production. Figure

S5 has (antibiotic sales in mg/PCU + 1). I am not sure how the authors have managed to adjust the drug use to the size of animal production then. "1 PCU = 1 kilogram of biomass of different categories of livestock and slaughtered animals". This needs explaining and if it is not adjusted to production size / number of animals / meat produced / sold or similar, then than sub-plot can be removed. The lines being higher on the Y axis in one animal is then not very informative.

Answer) The caption for Figure S5(A) was corrected to read “the amount of antibiotics sold (ton)”, which is consistent with Figure S3.
