# Supplementary material for: Antibiotic resistomes discovered in the gut microbiomes of Korean swine and cattle
Source: Gigascience. 2020 May 5;9(5):giaa043. doi: 10.1093/gigascience/giaa043 (PMC7317084; doi:10.1093/gigascience/giaa043)
Supplement: giaa043_Supplemental_Figures_and_Tables [file giaa043_supplemental_figures_and_tables.zip › AR_Farm_Animals_Supplementary_Figure_0316.pptx]

## Slide 1
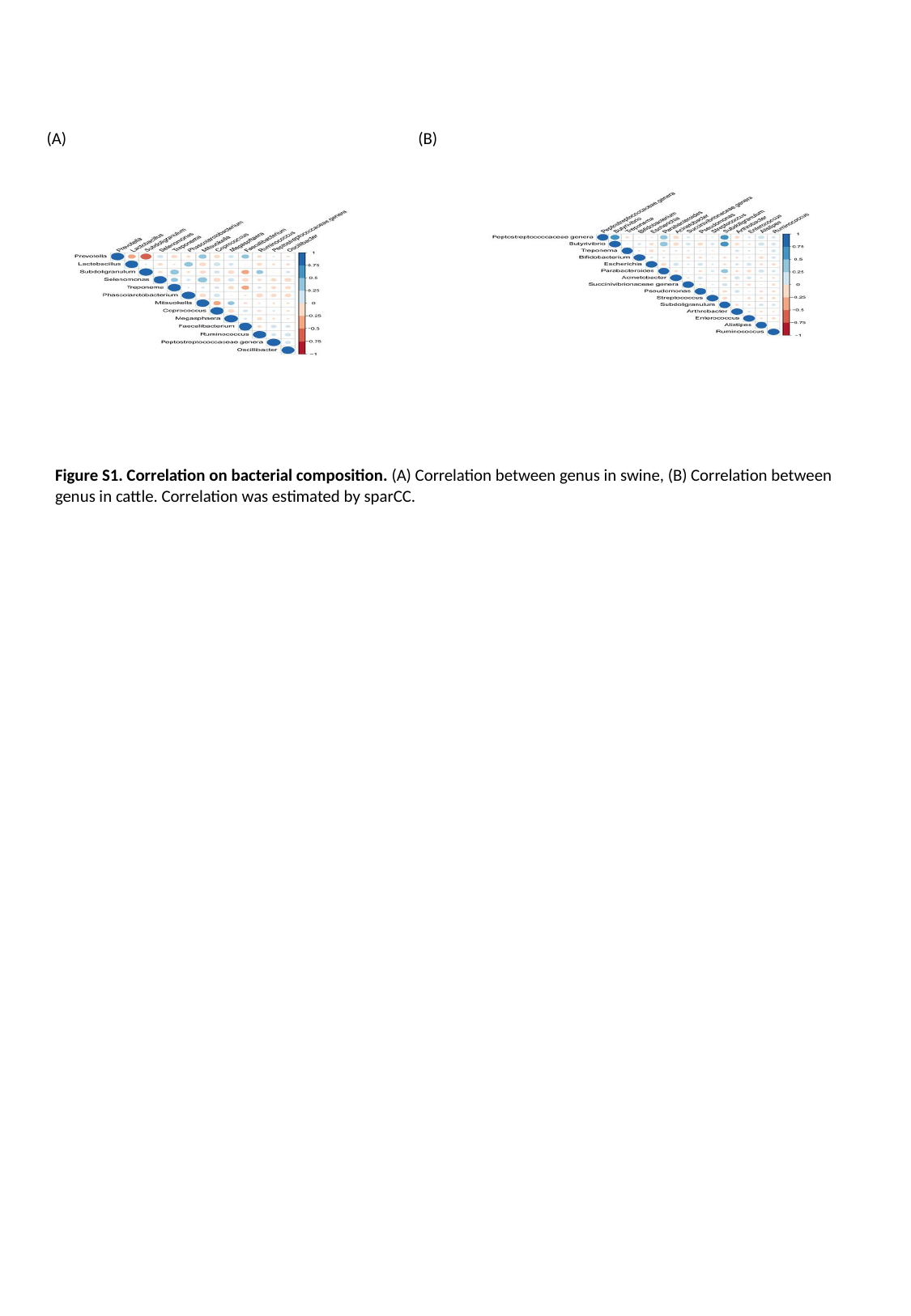

(B)
(A)
Figure S1. Correlation on bacterial composition. (A) Correlation between genus in swine, (B) Correlation between genus in cattle. Correlation was estimated by sparCC.

## Slide 2
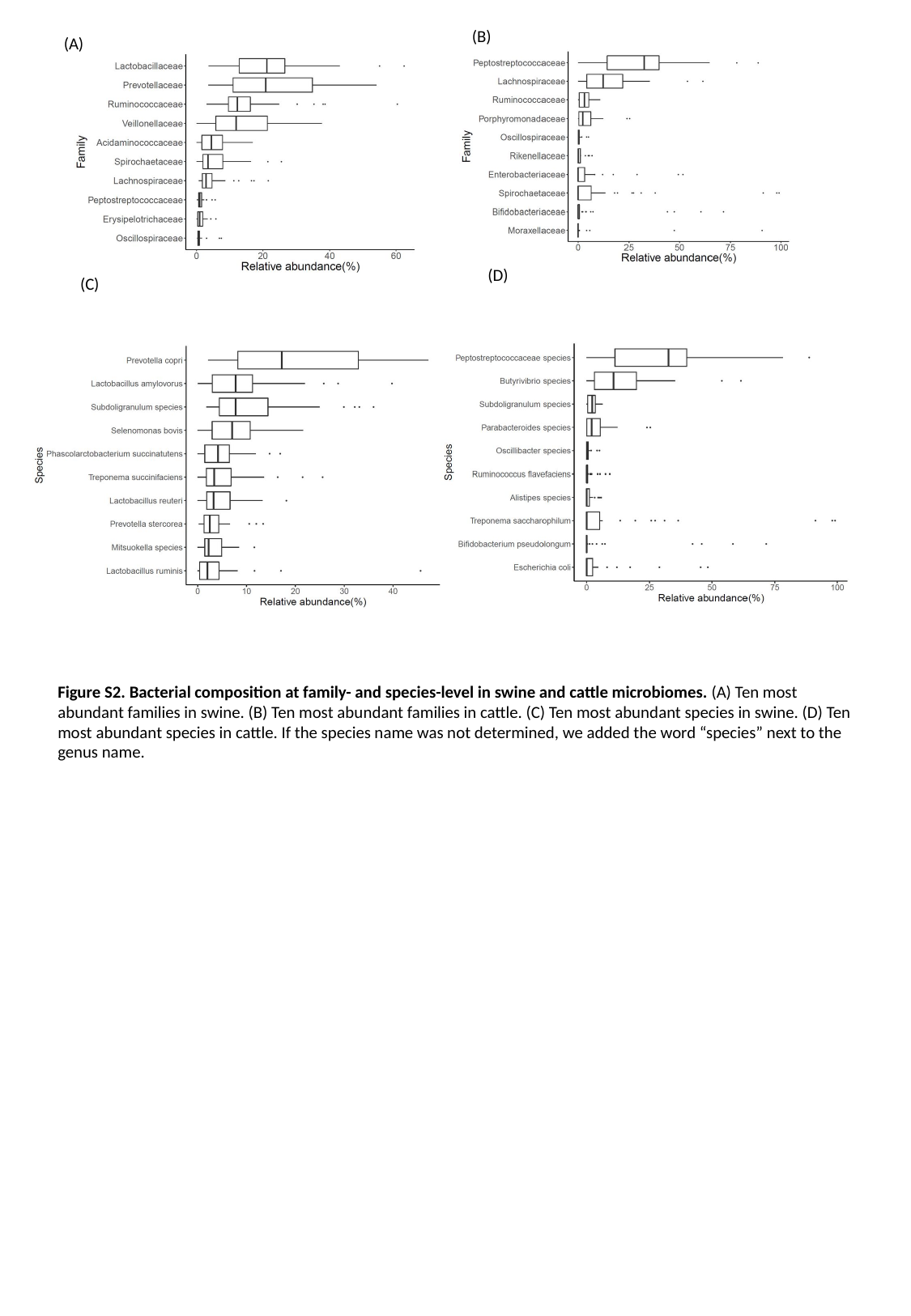

(B)
(A)
(D)
(C)
Figure S2. Bacterial composition at family- and species-level in swine and cattle microbiomes. (A) Ten most abundant families in swine. (B) Ten most abundant families in cattle. (C) Ten most abundant species in swine. (D) Ten most abundant species in cattle. If the species name was not determined, we added the word “species” next to the genus name.

## Slide 3
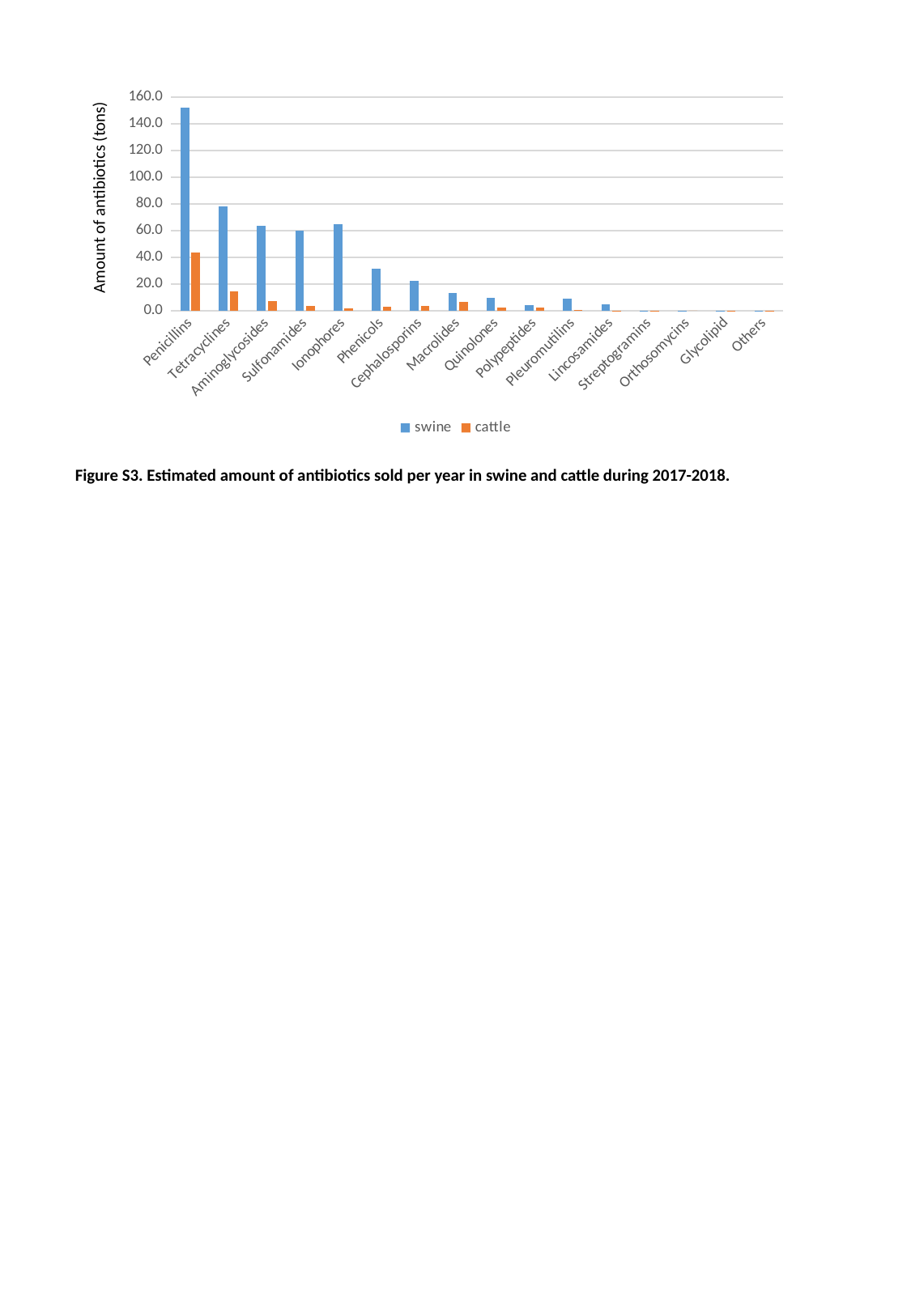

### Chart
| Category | swine | cattle |
|---|---|---|
| Penicillins | 151.8595 | 43.484300000000005 |
| Tetracyclines | 78.4585 | 14.551 |
| Aminoglycosides | 63.4565 | 7.0705 |
| Sulfonamides | 59.969 | 3.523 |
| Ionophores | 64.954 | 1.5925 |
| Phenicols | 31.3465 | 3.1645 |
| Cephalosporins | 22.548 | 3.4275 |
| Macrolides | 13.5535 | 6.414 |
| Quinolones | 9.651 | 2.0885 |
| Polypeptides | 4.389 | 2.326 |
| Pleuromutilins | 8.747 | 0.3235 |
| Lincosamides | 4.91 | 0.1805 |
| Streptogramins | 0.075 | 0.04 |
| Orthosomycins | 0.11 | 0.0 |
| Glycolipid | 0.04 | 0.052 |
| Others | 0.039 | 0.0955 |Amount of antibiotics (tons)
Figure S3. Estimated amount of antibiotics sold per year in swine and cattle during 2017-2018.

## Slide 4
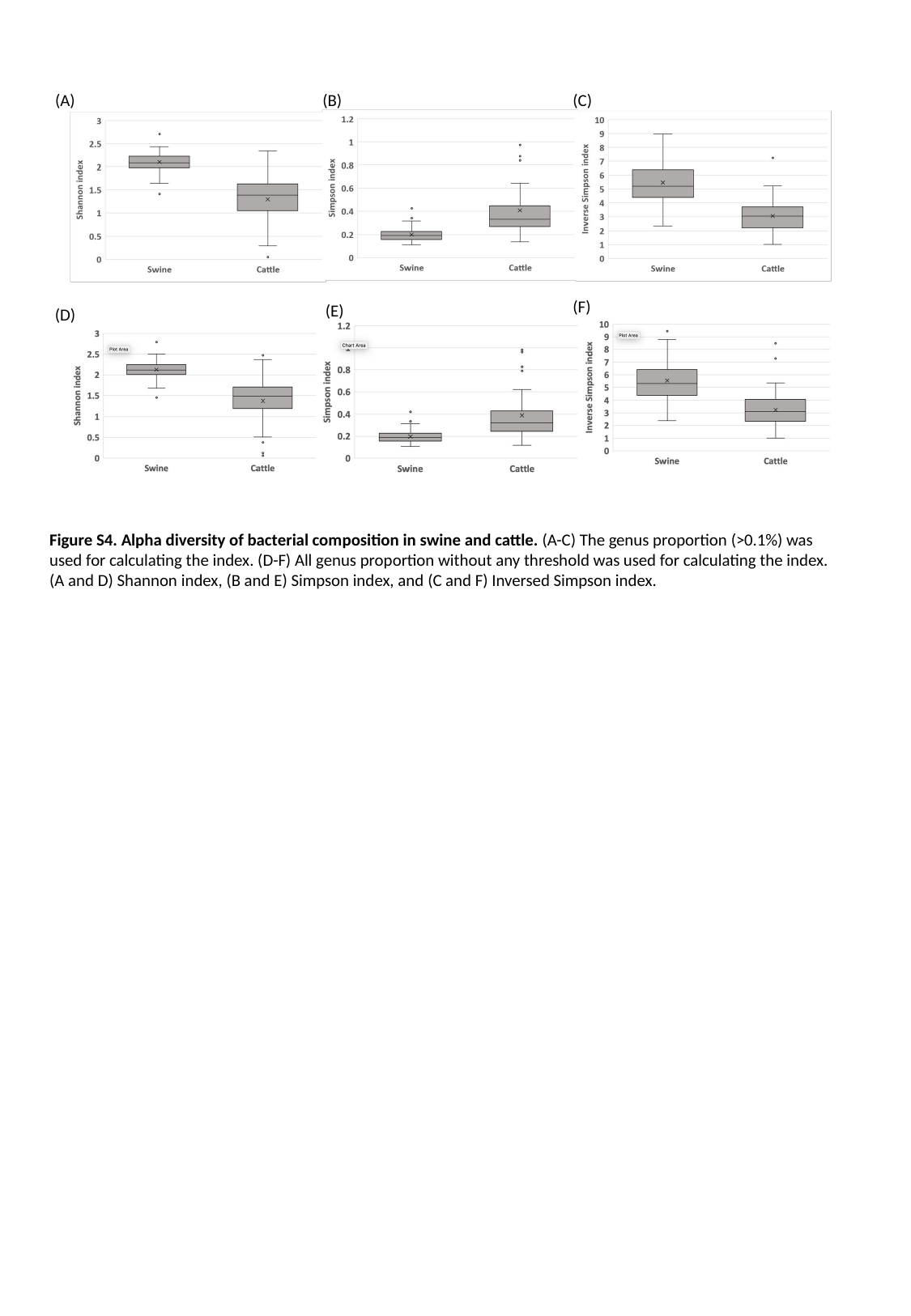

(A)
(B)
(C)
(F)
(E)
(D)
Figure S4. Alpha diversity of bacterial composition in swine and cattle. (A-C) The genus proportion (>0.1%) was used for calculating the index. (D-F) All genus proportion without any threshold was used for calculating the index. (A and D) Shannon index, (B and E) Simpson index, and (C and F) Inversed Simpson index.

## Slide 5
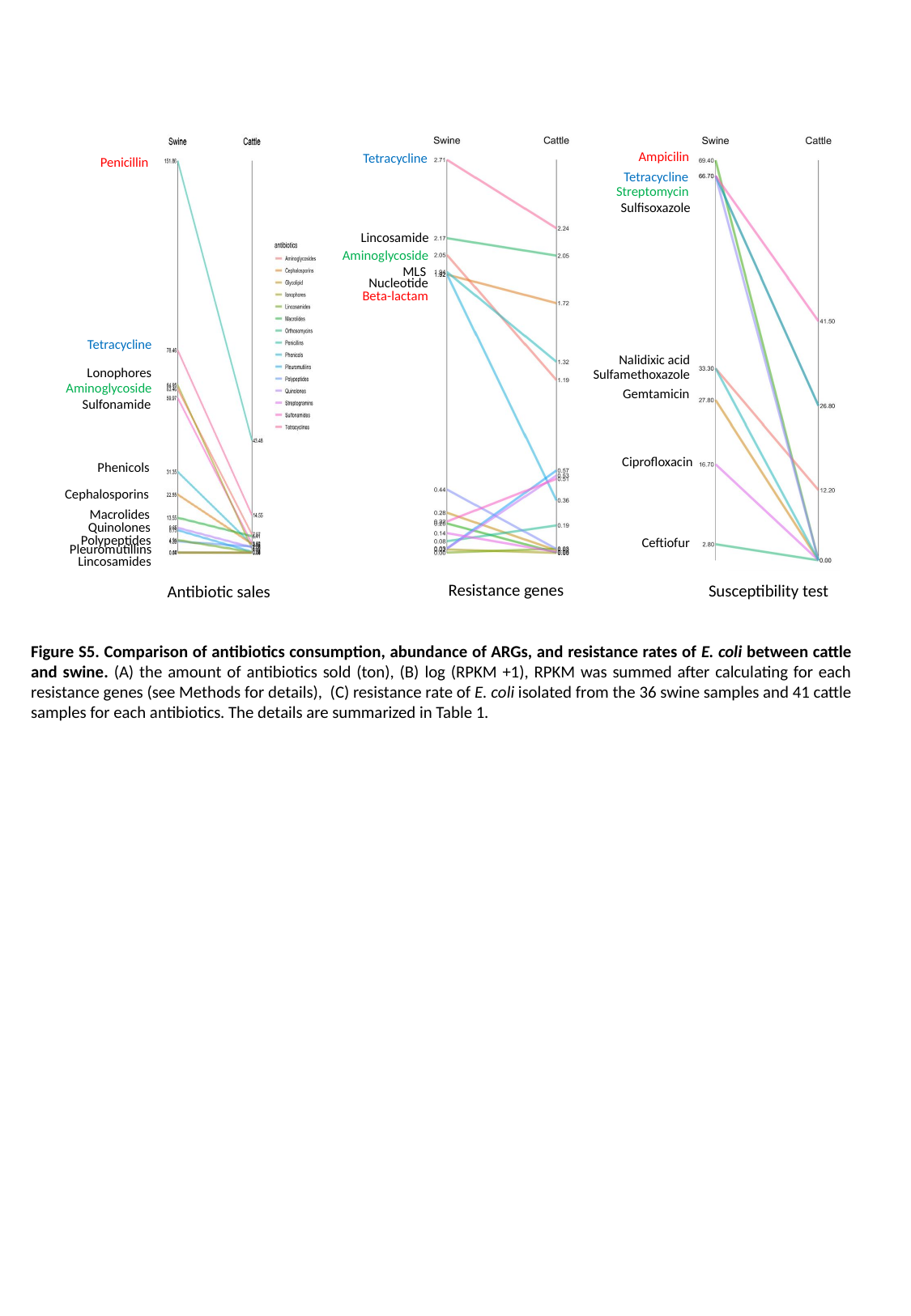

Ampicilin
Tetracycline
Penicillin
Tetracycline
Streptomycin
Sulfisoxazole
Lincosamide
Aminoglycoside
MLS
Nucleotide
Beta-lactam
Tetracycline
Nalidixic acid
Lonophores
Sulfamethoxazole
Aminoglycoside
Gemtamicin
Sulfonamide
Ciprofloxacin
Phenicols
Cephalosporins
Macrolides
Quinolones
Polypeptides
Ceftiofur
Pleuromutillins
Lincosamides
Resistance genes
Susceptibility test
Antibiotic sales
Figure S5. Comparison of antibiotics consumption, abundance of ARGs, and resistance rates of E. coli between cattle and swine. (A) the amount of antibiotics sold (ton), (B) log (RPKM +1), RPKM was summed after calculating for each resistance genes (see Methods for details), (C) resistance rate of E. coli isolated from the 36 swine samples and 41 cattle samples for each antibiotics. The details are summarized in Table 1.
