# Supplementary material for: Antibiotic resistomes discovered in the gut microbiomes of Korean swine and cattle
Source: Gigascience. 2020 May 5;9(5):giaa043. doi: 10.1093/gigascience/giaa043 (PMC7317084; doi:10.1093/gigascience/giaa043)
Supplement: giaa043_Supplemental_Figures_and_Tables [file giaa043_supplemental_figures_and_tables.zip › AR_Farm_Animals_Supplementary_Table_0229.docx]

Table S1. Sequencing data information for swine and cattle gut microbiome

| **Group** | **Farm** | **Name** | **# of raw reads** | **# of raw bases (bp)** | **# of trimmed reads** | **# of trimmed bases (bp)** | **# of contigs (>500bp)** | **# of Genes** |
| --- | --- | --- | --- | --- | --- | --- | --- | --- |
|  |  |  |  |  |  |  |  |  |
| Swine | A | A_1 | 89,332,164 | 13,489,156,764 | 85,636,070 | 12,807,497,424 | 629,443 | 1,309,608 |
| Swine | A | A_4 | 90,709,554 | 13,697,142,654 | 86,202,398 | 12,864,447,258 | 467,479 | 967,704 |
| Swine | A | A_5 | 74,611,368 | 11,266,316,568 | 70,803,740 | 10,562,761,824 | 558,447 | 1,128,253 |
| Swine | B | B_1 | 89,042,028 | 13,445,346,228 | 85,849,860 | 12,852,621,702 | 547,201 | 1,143,452 |
| Swine | B | B_2 | 77,914,506 | 11,765,090,406 | 73,797,882 | 11,017,103,540 | 492,096 | 1,060,987 |
| Swine | B | B_5 | 93,639,322 | 14,139,537,622 | 89,118,724 | 13,318,146,544 | 591,611 | 1,246,238 |
| Swine | C | C_33 | 61,345,982 | 9,263,243,282 | 55,323,138 | 8,193,762,440 | 445,483 | 914,185 |
| Swine | C | C_34 | 59,090,360 | 8,922,644,360 | 52,439,742 | 7,742,200,645 | 436,406 | 907,977 |
| Swine | C | C_35 | 63,515,794 | 9,590,884,894 | 54,882,092 | 8,077,071,922 | 463,552 | 927,528 |
| Swine | D | D_49 | 55,002,294 | 8,305,346,394 | 51,370,436 | 7,650,261,360 | 484,938 | 943,295 |
| Swine | D | D_50 | 75,967,074 | 11,471,028,174 | 67,814,240 | 10,053,994,640 | 544,592 | 1,116,232 |
| Swine | D | D_51 | 64,859,170 | 9,793,734,670 | 57,923,184 | 8,566,567,235 | 470,703 | 946,222 |
| Swine | E | E_63 | 57,667,542 | 8,707,798,842 | 50,236,666 | 7,404,392,723 | 450,833 | 902,822 |
| Swine | E | E_64 | 74,641,476 | 11,270,862,876 | 64,556,350 | 9,510,010,904 | 518,361 | 1,034,388 |
| Swine | E | E_65 | 72,803,650 | 10,993,351,150 | 60,269,742 | 8,830,991,783 | 461,598 | 917,158 |
| Swine | F | F_79 | 96,950,226 | 14,639,484,126 | 74,442,040 | 10,830,181,899 | 546,787 | 1,106,951 |
| Swine | F | F_80 | 94,246,552 | 14,231,229,352 | 80,047,344 | 11,771,288,692 | 503,621 | 1,037,146 |
| Swine | F | F_81 | 70,841,210 | 10,697,022,710 | 58,287,256 | 8,544,959,122 | 444,772 | 905,921 |
| Swine | G | G_92 | 80,944,400 | 12,222,604,400 | 69,707,920 | 10,278,302,037 | 518,521 | 1,053,943 |
| Swine | G | G_88 | 72,417,640 | 10,935,063,640 | 65,917,438 | 9,782,928,716 | 518,489 | 1,056,520 |
| Swine | G | G_90 | 81,040,754 | 12,237,153,854 | 69,712,050 | 10,276,539,517 | 559,472 | 1,078,186 |
| Swine | H | H_98 | 71,322,784 | 10,769,740,384 | 59,562,700 | 8,759,816,759 | 453,192 | 904,061 |
| Swine | H | H_100 | 73,955,592 | 11,167,294,392 | 66,327,338 | 9,820,927,706 | 496,461 | 1,017,750 |
| Swine | H | H_101 | 69,777,314 | 10,536,374,414 | 63,635,518 | 9,441,158,832 | 446,830 | 937,185 |
| Swine | I | I_133 | 88,766,780 | 13,403,783,780 | 72,046,578 | 10,538,041,242 | 576,956 | 1,172,428 |
| Swine | I | I_134 | 97,108,382 | 14,663,365,682 | 81,126,868 | 11,893,887,956 | 439,803 | 890,489 |
| Swine | I | I_118 | 69,353,342 | 10,472,354,642 | 57,291,836 | 8,393,772,737 | 347,928 | 726,157 |
| Swine | J | J_133 | 82,666,040 | 12,482,572,040 | 75,378,654 | 11,191,609,025 | 549,277 | 1,165,123 |
| Swine | J | J_134 | 86,840,432 | 13,112,905,232 | 74,128,820 | 10,908,997,564 | 550,655 | 1,132,973 |
| Swine | J | J_136 | 78,631,086 | 11,873,293,986 | 68,155,062 | 10,060,076,394 | 533,496 | 1,067,982 |
| Swine | K | K_148 | 75,933,280 | 11,465,925,280 | 71,532,572 | 10,706,351,272 | 534,562 | 1,003,115 |
| Swine | K | K_150 | 100,827,168 | 15,224,902,368 | 92,547,634 | 13,795,522,689 | 694,841 | 1,332,980 |
| Swine | K | K_153 | 101,100,728 | 15,266,209,928 | 94,341,690 | 14,078,719,687 | 781,712 | 1,528,415 |
| Swine | L | L_166 | 91,040,076 | 13,747,051,476 | 84,528,372 | 12,608,493,166 | 574,920 | 1,165,570 |
| Swine | L | L_169 | 114,488,998 | 17,287,838,698 | 106,143,376 | 15,828,489,786 | 742,116 | 1,480,312 |
| Swine | L | L_171 | 82,849,038 | 12,510,204,738 | 77,762,034 | 11,615,682,529 | 584,423 | 1,193,919 |

| Cattle | A | 2 | 75,236,568 | 11,360,721,768 | 69,726,120 | 10,365,274,403 | 646,704 | 1,199,883 |
| --- | --- | --- | --- | --- | --- | --- | --- | --- |
| Cattle | A | 4 | 111,242,676 | 16,797,644,076 | 105,150,680 | 15,744,688,437 | 633,401 | 1,404,487 |
| Cattle | A | 6 | 68,489,660 | 10,341,938,660 | 64,693,246 | 9,683,439,597 | 482,577 | 1,051,977 |
| Cattle | B | 11 | 156,329,068 | 23,605,689,268 | 149,232,702 | 22,408,172,503 | 601,270 | 1,207,287 |
| Cattle | B | 12 | 73,359,982 | 11,077,357,282 | 69,754,042 | 10,453,491,611 | 561,511 | 1,099,952 |
| Cattle | B | 16 | 80,598,484 | 12,170,371,084 | 76,832,748 | 11,519,551,101 | 636,529 | 1,224,822 |
| Cattle | C | 24 | 94,431,382 | 14,259,138,682 | 89,385,402 | 13,366,557,972 | 775,747 | 1,428,209 |
| Cattle | C | 25 | 76,464,640 | 11,546,160,640 | 73,075,086 | 10,955,909,785 | 690,862 | 1,289,052 |
| Cattle | C | 26 | 85,312,970 | 12,882,258,470 | 82,137,720 | 12,316,877,178 | 685,405 | 1,316,032 |
| Cattle | D | 33 | 75,450,912 | 11,393,087,712 | 71,629,904 | 10,739,129,099 | 475,069 | 1,027,700 |
| Cattle | D | 34 | 82,289,508 | 12,425,715,708 | 77,546,242 | 11,585,892,072 | 509,416 | 1,053,979 |
| Cattle | D | 37 | 105,440,164 | 15,921,464,764 | 100,619,332 | 15,075,527,513 | 719,003 | 1,452,387 |
| Cattle | E | 43 | 94,839,554 | 14,320,772,654 | 89,941,336 | 13,464,321,703 | 692,065 | 1,404,257 |
| Cattle | E | 44 | 80,350,442 | 12,132,916,742 | 75,901,352 | 11,360,927,692 | 574,009 | 1,200,943 |
| Cattle | E | 45 | 79,191,342 | 11,957,892,642 | 75,083,586 | 11,256,259,074 | 715,845 | 1,376,949 |
| Cattle | E | 46 | 90,286,152 | 13,633,208,952 | 85,958,912 | 12,878,948,574 | 757,478 | 1,481,833 |
| Cattle | F | 50 | 65,012,678 | 9,816,914,378 | 61,264,880 | 9,118,871,086 | 544,464 | 968,624 |
| Cattle | F | 51 | 74,398,194 | 11,234,127,294 | 70,570,408 | 10,575,054,609 | 639,168 | 1,160,602 |
| Cattle | F | 53 | 78,987,734 | 11,927,147,834 | 75,248,694 | 11,285,693,454 | 700,007 | 1,284,955 |
| Cattle | G | 59 | 71,133,780 | 10,741,200,780 | 66,758,788 | 9,991,476,054 | 576,551 | 1,112,051 |
| Cattle | G | 60 | 88,866,108 | 13,418,782,308 | 83,652,042 | 12,487,423,470 | 620,979 | 1,299,980 |
| Cattle | G | 61 | 69,155,188 | 10,442,433,388 | 65,741,288 | 9,848,054,284 | 586,250 | 1,132,209 |
| Cattle | G | 63 | 66,481,522 | 10,038,709,822 | 61,900,064 | 9,252,129,236 | 558,792 | 1,066,899 |
| Cattle | H | 70 | 86,588,732 | 13,074,898,532 | 81,805,292 | 12,221,921,975 | 688,215 | 1,322,335 |
| Cattle | H | 72 | 94,665,266 | 14,294,455,166 | 89,806,338 | 13,447,195,652 | 732,172 | 1,450,112 |
| Cattle | H | 74 | 76,704,094 | 11,582,318,194 | 73,174,778 | 10,958,931,584 | 642,317 | 1,235,929 |
| Cattle | I | 80 | 108,518,056 | 16,386,226,456 | 103,369,172 | 15,473,740,479 | 801,489 | 1,627,469 |
| Cattle | I | 81 | 122,419,854 | 18,485,397,954 | 115,889,126 | 17,354,437,357 | 964,800 | 1,872,210 |
| Cattle | I | 83 | 68,120,686 | 10,286,223,586 | 64,499,854 | 9,659,525,482 | 557,524 | 1,127,653 |
| Cattle | J | 90 | 76,005,432 | 11,476,820,232 | 72,886,680 | 10,968,688,159 | 586,996 | 1,220,852 |
| Cattle | J | 92 | 76,890,536 | 11,610,470,936 | 73,841,880 | 11,113,632,132 | 767,030 | 1,516,850 |
| Cattle | J | 93 | 76,014,106 | 11,478,130,006 | 72,982,348 | 10,982,852,461 | 666,963 | 1,348,958 |
| Cattle | K | 101 | 76,597,848 | 11,566,275,048 | 74,047,024 | 11,147,591,598 | 769,921 | 1,489,502 |
| Cattle | K | 102 | 75,741,162 | 11,436,915,462 | 71,984,504 | 10,807,281,823 | 251,719 | 501,557 |
| Cattle | K | 104 | 76,370,512 | 11,531,947,312 | 72,676,890 | 10,936,498,266 | 547,071 | 1,137,991 |
| Cattle | L | 114 | 76,246,812 | 11,513,268,612 | 73,094,486 | 10,996,561,299 | 701,097 | 1,376,415 |
| Cattle | L | 116 | 75,334,190 | 11,375,462,690 | 72,626,046 | 10,932,556,996 | 701,789 | 1,339,260 |
| Cattle | L | 119 | 76,749,714 | 11,589,206,814 | 73,862,128 | 11,117,164,299 | 712,283 | 1,401,513 |
| Cattle | M | 121 | 77,770,840 | 11,743,396,840 | 74,646,768 | 11,232,299,191 | 696,225 | 1,334,771 |
| Cattle | M | 123 | 77,190,740 | 11,655,801,740 | 72,285,154 | 10,836,839,750 | 611,370 | 1,241,860 |
| Cattle | M | 125 | 73,135,956 | 11,043,529,356 | 68,321,658 | 10,246,038,479 | 496,191 | 1,012,015 |

Table S2. Sample information for cattle

| **Samples** | **Farm ID** | **Farm size**  **(heads)** | **Age**  **(month)** | **Sex** | **Sampling date** | **Farm size**  **(heads)** |
| --- | --- | --- | --- | --- | --- | --- |
| 2 | A | 100 | 62 | F | 2018.4.10 | 100 |
| 4 | A |  | 19 | M | 2018.4.10 | 100 |
| 6 | A |  | 18 | M | 2018.4.10 | 100 |
| 11 | B | 106 | 22 | M | 2018.4.10 | 106 |
| 12 | B |  | 24 | M | 2018.4.10 | 106 |
| 16 | B |  | 25 | M | 2018.4.10 | 106 |
| 22 | C | 162 | 42 | F | 2018.4.10 | 162 |
| 23 | C |  | 69 | F | 2018.4.10 | 162 |
| 24 | C |  | 36 | F | 2018.4.10 | 162 |
| 31 | D | 130 | 30 | F | 2018.4.18 | 130 |
| 32 | D |  | 40 | F | 2018.4.18 | 130 |
| 35 | D |  | 60 | F | 2018.4.18 | 130 |
| 42 | E | 35 | 25 | F | 2018.4.18 | 35 |
| 43 | E |  | 45 | M | 2018.4.18 | 35 |
| 44 | E |  | 48 | M | 2018.4.18 | 35 |
| 52 | F | 350 | 25 | F | 2018.4.23 | 350 |
| 53 | F |  | 25 | F | 2018.4.23 | 350 |
| 55 | F |  | 22 | F | 2018.4.23 | 350 |
| 61 | G | 198 | 26 | F | 2018.4.25 | 198 |
| 63 | G |  | 25 | M | 2018.4.25 | 198 |
| 65 | G |  | 24 | M | 2018.4.25 | 198 |
| 72 | H | 40 | 50 | F | 2018.4.27 | 40 |
| 74 | H |  | 90 | F | 2018.4.27 | 40 |
| 76 | H |  | 70 | F | 2018.4.27 | 40 |
| 81 | I | 350 | 22 | F | 2018.4.27 | 350 |
| 82 | I |  | 23 | M | 2018.4.27 | 350 |
| 84 | I |  | 27 | M | 2018.4.27 | 350 |
| 91 | J | 98 | 23 | M | 2018.6.26 | 98 |
| 93 | J |  | 28 | M | 2018.6.26 | 98 |
| 94 | J |  | 28 | M | 2018.6.26 | 98 |
| 102 | K | 109 | 27 | M | 2018.6.26 | 109 |
| 103 | K |  | 27 | M | 2018.6.26 | 109 |
| 105 | K |  | 29 | M | 2018.6.26 | 109 |
| 112 | L | 93 | 27 | M | 2018.6.27 | 93 |
| 114 | L |  | 29 | M | 2018.6.27 | 93 |
| 117 | L |  | 33 | M | 2018.6.27 | 93 |
| 122 | M | 15 | 31 | M | 2018.6.27 | 15 |
| 124 | M |  | 30 | M | 2018.6.27 | 15 |
| 125 | M |  | 32 | M | 2018.6.27 | 15 |

Table S3. Sample information for swine

| **Samples** | **Farm ID** | **Age(day)** | **Sampling date** | **Farm size(heads)** |
| --- | --- | --- | --- | --- |
| A_1 | A | 150-200 | 2017.8.9 | 20,000 |
| A_4 | A | 150-200 | 2017.8.9 |  |
| A_5 | A | 150-200 | 2017.8.9 |  |
| B_1 | B | 150-200 | 2017.8.9 | 1,200 |
| B_2 | B | 150-200 | 2017.8.9 |  |
| B_5 | B | 170 | 2017.8.9 |  |
| C_33 | C | 180 | 2017.8.24 | 3,500 |
| C_34 | C | 180 | 2017.8.24 |  |
| C_35 | C | 180 | 2017.8.24 |  |
| D_49 | D | 165 | 2017.8.24 | 4,000 |
| D_50 | D | 165 | 2017.8.24 |  |
| D_51 | D | 165 | 2017.8.24 |  |
| E_63 | E | 170 | 2017.8.29 | 4,500 |
| E_64 | E | 170 | 2017.8.29 |  |
| E_65 | E | 170 | 2017.8.29 |  |
| F_79 | F | 170 | 2017.8.29 | 4,000 |
| F_80 | F | 170 | 2017.8.29 |  |
| F_81 | F | 170 | 2017.8.29 |  |
| G_88 | G | 150 | 2017.8.31 | 3,500 |
| G_90 | G | 150 | 2017.8.31 |  |
| G_92 | G | 150 | 2017.8.31 |  |
| H_98 | H | 190 | 2017.8.31 | 1,500 |
| H_100 | H | 190 | 2017.8.31 |  |
| H_101 | H | 190 | 2017.8.31 |  |
| I_113 | I | 180 | 2017.10.16 | 6,500 |
| I_114 | I | 180 | 2017.10.16 |  |
| I_118 | I | 180 | 2017.10.16 |  |
| J_133 | J | 230 | 2017.10.16 | 1,100 |
| J_134 | J | 230 | 2017.10.16 |  |
| J_136 | J | 230 | 2017.10.16 |  |
| K_148 | K | 210 | 2017.10.25 | 150 |
| K_150 | K | 210 | 2017.10.25 |  |
| K_153 | K | 210 | 2017.10.25 |  |
| L_166 | L | 190 ~200 | 2017.10.26 | 1,200 |
| L_169 | L | 190 ~200 | 2017.10.26 |  |
| L_171 | L | 190 ~200 | 2017.10.26 |  |

Table S4. Abundance of antibiotic resistance class in swine

| AR class | Average | Median | Max | Min |
| --- | --- | --- | --- | --- |
| Aminoglycoside | 112.037 | 82.96405 | 554.9763 | 16.18745 |
| Beta-lactam | 81.83332 | 84.17416 | 164.9878 | 15.06674 |
| Diaminopyrimidine | 0.892718 | 0 | 13.96591 | 0 |
| Fluoroquinolone | 0.055791 | 0 | 1.179088 | 0 |
| Fosfomycin | 0.004584 | 0 | 0.165015 | 0 |
| Glycopeptide | 0.595113 | 0.207118 | 10.35973 | 0 |
| Lincosamide | 146.5146 | 142.68 | 226.7659 | 35.754 |
| Macrolide | 0.203409 | 0 | 1.026802 | 0 |
| MLS | 85.3374 | 77.54108 | 201.1947 | 22.78825 |
| Nucleoside | 82.15096 | 86.52513 | 167.5797 | 4.420035 |
| Peptide | 0.072671 | 0 | 0.962711 | 0 |
| Phenicol | 1.735139 | 1.573453 | 9.240042 | 0 |
| Polymyxin | 0.069898 | 0 | 0.99098 | 0 |
| Streptogramin | 0.368319 | 0 | 4.641171 | 0 |
| Sulfonamide | 0.644206 | 0.473973 | 3.237333 | 0 |
| Tetracycline | 510.768 | 513.5857 | 735.0687 | 235.6892 |

Table S5. Abundance of antibiotic resistance class in cattle

| AR class | Average | Median | Max | Min |
| --- | --- | --- | --- | --- |
| Aminoglycoside | 14.52401 | 7.169168 | 250.5528 | 0.337853 |
| Beta-lactam | 51.56143 | 47.50143 | 117.3394 | 16.38807 |
| Diaminopyrimidine | 0.040512 | 0 | 0.757068 | 0 |
| Fluoroquinolone | 0 | 0 | 0 | 0 |
| Fosfomycin | 0.06884 | 0 | 1.707749 | 0 |
| Glycopeptide | 0.00515 | 0 | 0.211138 | 0 |
| Lincosamide | 110.7985 | 100.6741 | 253.7977 | 19.61995 |
| Macrolide | 0.547237 | 0 | 15.75983 | 0 |
| MLS | 19.73986 | 14.87418 | 54.32585 | 1.650383 |
| Nucleoside | 1.301568 | 0.661189 | 9.965543 | 0 |
| Peptide | 2.699918 | 0 | 104.2819 | 0 |
| Phenicol | 0.063992 | 0 | 1.089959 | 0 |
| Polymyxin | 2.41215 | 0 | 93.63014 | 0 |
| Streptogramin | 0.013909 | 0 | 0.570259 | 0 |
| Sulfonamide | 2.23741 | 0 | 29.99811 | 0 |
| Tetracycline | 171.5553 | 172.1516 | 282.8193 | 61.36273 |
