# Supplementary material for: Antibiotic resistomes discovered in the gut microbiomes of Korean swine and cattle
Source: Gigascience. 2020 May 5;9(5):giaa043. doi: 10.1093/gigascience/giaa043 (PMC7317084; doi:10.1093/gigascience/giaa043)
Supplement: giaa043_Response_to_Reviewer_Comments_Original_Submission [file giaa043_response_to_reviewer_comments_original_submission.pdf]

Author's Response To Reviewer Comments

Close

Dear Reviewer and Editor,

Enclosed please find the revised version of the above titled manuscript that we are submitting for your consideration for publication in Giga Science. We very much appreciate the helpful comments from two reviewers. Listed below are our responses.

Reviewer #1: In the manuscript entitled "Antibiotic resistomes discovered in the gut mirobiomes of swine and cattle", Lim et al. applied deep sequencing and metagenomic analyses on 36 swine and 41 cattle feces from 25 feedlots in South Korea to characterize and compare prevalence and diversity of antibiotic resistance genes in these animals. Authors found that prevalence of resistance is higher in swines. They identified aminoglycoside resistance as the most prevalent both in cattle and swine but involving different gene families.

General comments
Nowadays, the topic of antimicrobial resistance (AMR) is becoming one of the most critical public health issues and it is crucial to better understand the distribution of resistance determinants and identify the reservoirs and the routes of dissemination involved in AMR emergence in a One Health approach. By performing shotgun metagenomics on a consequent number of samples (totals of 77 36 swine and 41 cattle feces metagenomes) collected nationwide, Lim et al., generated valuable data on a representative panel in this perspective. Sample information for swine and cattle are well presented in Table S2 and S3. All sequencing data were available at ENA (accession number PRJEB32496) and showed all a relevant sequencing depths for such resistome analyses. Interesting results are provided about the specific patterns of resistance in swine and cattle and with the identification of shared resistance gene sequence (for example tetQ and tetX) or specific sequence depending on animal species (tetM). Such results are important to better understand resistance gene prevalence and their dynamics among farm animals and generally in the environement.

1. The main limitation is that the manuscript in the current form remains too superficial in methodologies description and result discussion. First, bioinformatics and statistical methods performed on metagenomics data are not sufficiently detailed. For instance, there is also no clear description of the tools and statistics used for correlation studies between resistance classes and genus (which R package was used?) or for the network analysis. Thresholds and parameters used during metagenomics data processing (as for bacterial taxa identification with MetaPhlAn for instance) should also be added to the methods section to facilitate the reuse of the analysis for the reader. Please describe methodologies applied to metagenomics data and statistics with more details in the Methods section.

Answer) We very much appreciate the reviewer's thoughtful suggestions. Details of the method are now provided in the Methods section. In the revised manuscript, statistical analysis for the correlation of bacterial genera and the effects of feedlots on the bacterial distribution were added in lines 389-394. The network analysis was rewritten in lines 411-435. The method for bacterial taxa identification using MetaPhlAn was added in lines 380-382. The method for estimating resistome abundance was added in lines 397-408.

2. In addition, the presentation and interpretation of data could also be thorough. Results should be discussed in light of previous studies in an extensive manner. Some papers recently published on resistome studies in pig and/or cattle could be cited and used to confront results found here as for instance: PMID: 15066850; PMID: 30761096; PMID: 26952213; PMID: 31455230.

Answer) We have revised the Results and Discussion sections to include discussion and comparison with previous studies as the reviewer suggested (lines 174-180, 210-219, and 290-294).

Specific comments

3. Concerning the analysis of bacterial composition, how the sample locations (farms) impact the populations? There are especially 3 samples which are isolated on the bottom of the PCA (Fig. 2). Are these samples were sampled in the same feedlot? Do the authors have some elements to explain such microbiome specificities?

Answer) Among the 3 isolated samples, 2 were from the same farm and 1 was from another farm. The composition of these samples were observed to have different proportions of bacterial compositions in Figure 1(B). They have extremely high proportions of Treponema. In the revised version of the manuscript, we used metaMDS in R package to use nonmetric multidimensional scaling (NMDS) with factors in Figure 2(A). We found that the plot based on NMDS could better show the bacterial distribution among the samples.

To determine the impact of the sample location (farm), we preformed permutational multivariate analysis of variance (permanova). Bacterial compositions were statistically different among different farms for swine and cattle (p-value < 0.001 for swine; p-value < 0.001 for cattle). We added the results in the revised manuscript (lines 127-132).

4. The Spearman's correlation figure (ig. S1) is important and could be integrated in the core manuscript text rather than in supplementary materials.

Answer) The primary focus of this paper is resistome analysis, rather than microbial composition. As such, we believe that Figure S1 would fit better as a supplementary data, rather than being included as part of the main text.

5. Authors mentioned in Discussion that the study revealed a general correlation among antibiotic usages, resistance phenotypes, and resistome. This is a very interesting point but that should be supported by statistical analysis or at least with a dedicated figure summarizing and combining such data to clearly show this trend.

Answer) We added Figure S6 to show the trend of general association among antibiotic sales, resistance phenotypes, and resistome.

6. It would be interesting to have a discussion on the link between bacteria and resistance gene or, in other words, to investigate if resistance genes that are commonly found if swine and cattle, are carrying by the same bacteria in both animals.

Answer) In response to the reviewer’s suggestion, we investigated three aminoglycoside or tetracycline ARGs: ANT(6)-Ib, tetQ, and tetW genes. As shown in Figure 5, ANT(6)-Ib-homologous genes (72% homologous to the ANT(6)-Ib gene in CARD) that were found from both swine and cattle were clustered with 100% sequence similarity (cluster A(iv) in Figure 5). This pattern was also observed in fine-scale by using read mapping against a contig that contains the ANT(6)-Ib-homologous gene and was assembled from the sample. The contig was fully aligned with the reads from each sample of swine and cattle. Such observation might imply that the ANT(6)-Ib-homologous gene was carried by the same bacteria in both swine and cattle. For the tetQ gene (cluster C(i) in Figure 5)) and tetW gene (cluster D(i) in Figure 5) that were commonly found in swine samples, we also observed that the contig containing such ARGs were fully covered by the reads from both swine and cattle samples. Considering the relatively small number of contigs available in this study, however, we feel that a more comprehensive set of contigs are needed to draw any meaningful conclusion. Our future efforts will be directed in such directions. We thank the reviewer for bringing this interesting scientific question to our attention.

7. In discussion, authors referred to a previous study: "…, tetracycline genes were found in higher rate in cattle than I swine (Figure 6), which is consistent with a previous study" but there is no reference.

Answer) In this revised manuscript, we re-analyze the abundance of tetracycline genes by using the RPKM instead of using gene diversity (please see our response to Reviewer #2’s comment #1). The new results showed that the abundance of tetracycline measured by RPKM is higher in swine than in cattle (please see revised Figure 4E and F). We have revised the sentences to report this finding, and added the references accordingly (lines 207-212).

8. There is no line numbers that complicate the review.

Answer) We added the line numbers accordingly.

9. There are some typing errors along the paper, please correct. In addition, english revision of the text would improve clarity of the manuscript.

Answer) We thank the reviewer for her/his careful reading of our manuscript. Corrections were made by fixing grammatical/typographical errors.

10. Some references to figure S3A and B were made in "Pervasive antibiotic resistance genes in the gut microbiome of swine and cattle" of Analysis section instead of referring to figure 5 (fig. S3 indeed correspond to the amounts of antibiotic sold.) Please correct.

Answer) Corrections were made accordingly.

11. Gene names should be figured in italic.

Answer) Corrections were made accordingly throughout the manuscript.

Reviewer #2: GENERAL:
The study is primarily focused with characterizing 77 fecal samples from Korean cattle and swine through metagenomic sequencing. Analysis on both the resistome and general phylogentic distribution is performed and MIC panels are used on a collection of isolated E. coli.
The field work, lab work and bioinformatics analysis itself appears good, the network analysis of homologous genes is nice, but the downstream statistical analyses and interpretations need quite a bit of work. More details are also needed before the study could be considered reproducible. I recommend the authors add the following to the MS:

1. Actual abundance estimation of the individual ARGs in their samples (see specific comments later down). Basically the equivalent of metaphlan, but for ARGs - Map the reads to an ARG database like ResFinder or CARD or your metagenome-assembled-determined ARGs to determine how abundant they are. See e.g. how we have done it, but there are a lot of ways to estimate how abundant the genes are:

Munk, P., Knudsen, B. E., Lukjacenko, O., Duarte, A. S. R., Van Gompel, L., Luiken, R. E. C., … Aarestrup, F. M. (2018). Abundance and diversity of the faecal resistome in slaughter pigs and broilers in nine European countries. Nature Microbiology, 3(8), 898-908. https://doi.org/10.1038/s41564-018-0192-9

Answer) We very much appreciate the reviewer's comments. In this revised manuscript, actual abundance of ARGs was re-estimated using RPKM as suggested (lines 396-408 in the Method section). The analysis sections, "Pervasive antibiotic resistance genes in the gut microbiome of swine and cattle" and "Resistance gene families differentially enhanced in the gut microbiome of swine and cattle", were revised to report the new results. In addition, Figure 4 was revised accordingly.

2. Do testing on dissimilarity matrices based on the abundances - The authors can use e.g. permanova to see how much of the variation in their taxonomic and resistance abundances can be explained by e.g. host species and the specific farm. Because they have several animal samples from each farm, they can calculate if same-farm animals are more similar and if so, how much.

Answer) As suggested by the reviewer, we tested the effects of host species and farms on the variation in taxonomic and resistance abundance. We found that the effects of host species in taxonomic and resistance abundance were indeed significant (adonis2, $P<0.001$ for both). In addition, the effects of farm in taxonomic abundance and resistance abundance were significant for swain and cattle (adonis2, $P<0.001$). Revisions were made to report these results in lines 127-131 and 160-166.

3. Consider analyzing abundance matrices as compositional data (CODA). See recent work by e.g. Gloor and Calle on why this is appropriate and consider calculating e.g. alpha and beta diversities using the methods suggested by them. This could help especially with the authors current attempts of correlating different genera with each other, which is prone to spurious correlations.

Gloor, G. B., Macklaim, J. M., Pawlowsky-Glahn, V., & Egozcue, J. J. (2017). Microbiome datasets are compositional: And this is not optional. Frontiers in Microbiology, 8(NOV), 1-6. https://doi.org/10.3389/fmicb.2017.02224

Calle, M. L. (2019). Statistical Analysis of Metagenomics Data. Genomics & Informatics, 17(1), e6. https://doi.org/10.5808/GI.2019.17.1.e6

Answer) We thank the reviewer for bringing these papers to our attention. As suggested by the reviewer, we re-analyzed the compositional data (CODA) by using alpha and beta diversity based on the methods suggested in these references (lines 127-134 and Supplementary Figure S4). For alpha diversity, Shannon index and Simpson index were used. For beta diversity, nonmetric multidimensional scaling was used.

4. The written English in the article is not of high quality. In most places, I can guess the authors meaning, but in other places the meaning is uncertain. The manuscript would greatly benefit from a native English speaker going through and improving it.

Answer) For this revision, we used professional English editing service to improve the quality of the manuscript.

5. The genome assemblies, predicted genes and their translations are all available on the FTP server, which is much appreciated. In the spirit of sharing, it would be nice to also make e.g. read counts and calculated bacterial abundances /biom files from metaphlan avilable. These basically fit in small tab-delimited files or spreadsheets for people to easily review and analyse.

Answer) According to the reviewer's kind suggestion, we have uploaded the read counts and biom file to the FTP server.

6. Lastly, line numbers would have been much appreciated during review!

Answer) Line numbers were included in the revised manuscript.

SPECIFIC COMMENTS:
P2
7. The very first sentence of the abstract doesn't make sense to me. I think the authors mean that antimicrobial resistance genes are getting enriched in different reservoirs, not that the antibiotic molecules themselves are accumulating over time?

Answer) To clarify the meaning, we corrected the sentence to read "Antibiotics administered to the farm animals have led to increasing prevalence of resistance genes in different microbiomes and environments." (lines 14-15).

8. "deep sequencing on the gut microbiomes of 36 swine and 41 cattle in the farms,…" - I would already in this sentence mention that the scope of the study is specifically Korean farms.

Answer) We corrected the sentence to read "… we have performed deep sequencing on the gut microbiomes of 36 swine and 41 cattle in Korean farms." (line 20)

9. "Aminoglycoside resistance is the most abundant in both swine and cattle" - This implies higher number of genes which was not determined. The authors looked at the ARG richness (how many different genes), rather than abundance.

Answer) We rewrote the abstract to report the results from the ARG abundance analysis (lines 22-30).

P3
10. "antibiotic resistance genes are more frequently transferred from one bacteria to others in the gut microbiome" - not clear what this refers to. More frequently than what?

Answer) This sentence simply states the fact that antibiotic resistance genes have been transferred between bacteria. We did not intend to compare the frequency. To clarify the meaning, the sentence was revised to read "antibiotic resistance genes were transferred from one bacterium to others within the human microbiomes, and between human and livestock microbiomes" (lines 45-47).

11. "An interesting observation is the presence of two…" - I think this might be phrased slightly odd. It is not odd/interesting/surprising that some ARGs are unique to one of the livestock species and vice versa. That is quite common in these kind of studies.

Answer) We changed the sentence to read “We observed the presence of two different patterns of resistance genes: one type is host-dedicated; the other exists in different host animals.”

P4
12. "A total of 36 gut microbiome" - I think it should be "microbiomes" in plural throughout the manuscript.

Answer) Corrections were made accordingly.

13. "For each farm, three or four samples.." - I think explanations of sampling scheme, rationale etc. should be moved up to the "Data description" or keep it in methods, rather than having it in the "analysis" section.

Answer) We moved the sentence to the Methods section as suggested by reviewer (lines 323-325).

14. "The genus-level compositions were significantly different in swine and cattle" - If the authors say "significant", I think the author should add the name of the test and p-value, e.g. "significant (p=0.023, permanova / anosim / other test)". This also applies to other places in the MS where something is claimed to be significant.

Answer) We added the p-values from permanova test or t-test to denote statistical significance (lines 106, 132, 136, 240, 242, and 244).

P5
15. "… were the major genus in Firmicutes" - genus is "genera" (the authors have correctly used "genera" in other places). Firmicutes (a phylum) should not be in italic.

Answer) Corrections were made accordingly.

16. "Several genera showed high correlations in cattle" - Is this relevant? Don't we expect some genera to correlate in abundance simply by chance? Do we have more or less than expected by chance?

Answer) To address this point, we carried out spearman’s rank correlation test and performed linear regression (Figure S2 and S3). We thank the reviewer for raising this question, which prompted our detailed analysis that improved the quality of the manuscript.

17. "Treponema and Bifidobacterium were observed, but they constituted lower than 1% of the composition" (in cattle) - 2 sentences later, you say that Treponema range between 0% and 99% (!) in cattle. So I assume the first 1% refered to was the median? Looking at the variability and e.g. range of Treponema, I think it would be wise to establish whether something funny is going on. 99% Treponema sounds odd to me and it would be nice to hear the authors reflections on this. Also to see the metaphlan output available to verify independently.

Answer) The “1%” in the first line indicates the average proportion of Treponema and Bifidobacterium. To clarify this point, we revised the sentence to read “Even though Treponema and Bifidobacterium were observed as one of ten most abundant genera ordered by average proportion, their prevalence is lower than 50%: 19 and 14 out of 41 cattle samples, respectively“ (lines 120-122). We also confirmed that the maximum proportion of Treponema is 99% in one sample. In addition, the other two samples have more than 90% of Treponema in their gut microbiomes. It is true that this is an extremely high value, but we observed it in our analysis. We also uploaded the Metaphlan results in the FTP server.

18. "X was positively correlated with Y" - The authors should be aware that correlations between microbiome features can be hard to interpret because data is compositional - the flow cell can only sequence X DNA molecules that get read, so if bacterium A becomes more abundant, B gets displaced giving a natural tendency towards negative correlations. The different read counts within a single sample can thus not be considered independent.

Answer) We very much appreciate the reviewer’s legitimate concern. In order to address this point, we carried out linear regression to find statistically meaningful correlation between the two species. Figures S2 is now added in the revised manuscript to make this point clear. We thank the reviewer for her/his comment.

19. I strongly suggest re-naming "intra-individual diversity" to "alpha diversity" which is the common phrase of the within-sample distribution of organisms in ecology, also adopted by many microbiome studies.

Answer) Corrections were made accordingly.

20. "The average number of genera that constituted…" - I suggest calculating a more classic alpha diversity index (e.g. Shannon or Simpson diversity).

Answer) Following the reviewer's suggestion, we calculated Shannon index and Simpson index. The revised sentence now reads "The Shannon diversity for swine is 1.86, while that for cattle is 1.23 on average. Similarly, inverse Simpson index for swine is 4.91, while that for cattle is 2.96 on average." (lines 135-137). We also added Supplementary Figure S3 containing the distribution of alpha diversity throughout the samples.

21. "Normalized by the million genes predicted in the microbiome (GPM)" - I think the authors way of quantifying AMR in the samples is not appropriate or at least should not stand alone. MEGAHIT is fine for metagenomic assembly, but the authors are very strict in requiring the full length gene gets assembled in each sample. That lowers their detection threshold many fold. For taxonomic assignment, they match the reads directly to references (using metaphlan). Their GPM is actually not an "abundance" in that it doesn't capture how much resistance there is in each sample, but rather the ARG richness relative to total gene richness.
The GPM is fine to report, but without a relative abundance also, it is akin to omitting to report how much there is of each genera in their samples.
Therefore, I think the authors should also map reads directly to ARGs and account for differences in gene lengths and sequencing depth differences. If they then choose the conservative approach of only mapping reads to the genes they manage to assemble in some of their samples, that is okay. But I would suggest mapping their reads directly to a database such as ResFinder or CARD and analyzing the output.

Answer) We very much appreciate the reviewer's suggestions. In this revised manuscript, the actual abundance of ARGs was estimated using RPKM. In addition, the assembled genes were used to show the diversity of the resistance genes in swine and cattle (Supplementary Figure S4). The results are now rewritten in the analysis sections of "Pervasive antibiotic resistance genes in the gut microbiome of swine and cattle" and "Resistance gene families differentially enhanced in the gut microbiome of swine and cattle". Figure 3 was also replaced with the data of resistance gene abundance measured by RPKM.

22. "It implies that these six classes exist in more than half the population" - I do not understand this inference and would like it explained better.

Answer) We revised the sentence to read "It implies that the prevalence of each of these seven classes was higher than 50%." (lines 144-145).

23. "Moreover, the resistance rates were relatively lower…" - Does this refer to the proportion of animals in which the authors were able to assemble a gene of x phenotype?

Answer) This resistance rate is from the antimicrobial susceptibility test with E.coli isolated from the samples (Table 1). We revised the sentence to clarify the meaning, which now reads "Moreover, the resistance rates that were observed from the antimicrobial susceptibility test with E.coli were relatively lower in cattle, compared to those in the swine (Table 1) "(lines 152-153).

P6
24. "Among the three gene families…" - The term "gene family" is used inconsistently. In one sentence it is used to refer to all aminoglycoside nucleotidyltransferases, in the next it refers to ANT(6) genes.

Answer) Gene family is used to denote a collection of related resistance genes. For example, ANT(6) is a gene family that includes a collection of specific ANT(6) genes such as ANT(6)-Ia and ANT(6)-IIa. Corrections were made throughout the manuscript for consistency.

25. "The overall abundance of swine is evidently higher than that of cattle" - I assume abundance of ARGs is meant and not the actual pigs?

Answer) The sentence was revised to read “The overall abundance of ARGs in swine is evidently higher than that in cattle”

P9
26. When the authors say something "correlated", I think they should make it clear whether a correlation test was performed or there was an apparent association was determined visually.

Answer) We changed the term “correlation” to “association” on page 9. Throughout the revised manuscript, we used the term “correlated” only for the results of correlation test. Otherwise, we used the word “association” instead.

P10
27. "Low-quality reads were removed using Sickle" - How was the program run / with what parameters (same goes for megahit)? Is it correctly understood that entire reads were removed, rather than trimming to the high-quality parts of the reads? An example command would be nice for reproducibility. Were singleton reads kept if the mate was discarded? I know MEGAHIT can accommodate a combination of single and PE reads, but the details are not clear.

Answer) The option for sickle is pe -q 20 -t sanger -l 90, which trims the low quality part and removes the read if the read is shorter the length threshold of 90 bps (lines 350-353). Since we used the default option for MEGAHIT, only PE reads were used for assembly. The sentence was revised to clarify the threshold values (lines 412-414).

28. "An average of 160 M paired-reads (ranging between 110 M and 229 M)" - This does not agree with the supplementary Table S1. There, I see e.g. a line with 26M trimmed read pairs (sample C_34). Is this average, perhaps calculated per farm instead of per sample? If so, it should be changed to per sample/individual fecal sample.

Answer) The sentence was revised to read “An average of 38 M pairs of reads (ranging between 25 M and 75 M) were generated from each sample after filtering‟ (line 350-351).

29. It would be good to also mention what DNA library preparation kit was used for the sequencing: specifically, did it include a PCR amplification step?

Answer) We used TruSeq DNA PCR Free Kit (Illumina), and did not include PCR amplification step (lines 345-346).

P11
30. "Host contamination was removed by discarding the reads…" - What software, version, arguments was used against what exact NCBI accessions to complete this task?

Answer) We used bowtie2-align version 2.1.0 with the option of sensitive-local. The swine genome used was Swine –Sscrofa11.1 (GCF_000003025.6). The cattle genome used was Cattle - Bos_taurus_UMD_3.1.1 (GCF_000003025.6). We added this information in the revised manuscript (lines 555-558).

OTHER
31. On ENA, it seems like the reads are annotated as coming from a HiSeq4000 instrument. If so, should the manuscript reflect that too?

Answer) HiSeq4000 instrument was used for sequencing. This specification was included in the revised manuscript (line 344).

FIGURES AND TABLES:
32. Figure1: Not strictly required, but I think a different plotting program that allowed less white in-between the bars would make the figure much more readable.

Answer) According to the reviewer’s thoughtful suggestion, Figure 1 was revised to reduce the width of white space between the bars.

33. Figure S2: It seems like a lot is wrong with the caption and/or plots. The first sentence indicates the

overall plot contains phylum-level data, which is not the case. The sub-plots are wrongly referenced: (A) and (B) are both family-level taxonomy, so I assume (B) is Family in cattle, rather than species in swine? Also (A) and (B) seem to be entirely "family", while (C) and (D) are not really "species" , but some mixture of families, genera and species (see Peptostreptococaecae, Alistipes and E. coli in (D)).

Answer) The caption and the species names in Figure S2 were corrected. For some species, species in a specific genus was identified even though the species name was not determined. Such species was annotated as “Genus_species”. For example, Alistipes_species denotes a species in Alistipes genus.

34. Figure S3: Are there some kind of production values available for Korea that the authors could use to account for how much biomass/animal is consuming the antibiotics? The raw tonnage can be misleading in case the country produced 1000-fold more of one type of meat

Answer) Currently, we only have official statistics for the antibiotic sales for livestocks in Korea. How much of these antibiotics are actually administered over a given period is beyond our knowledge. At this point, this is the most information that we can collect. We will try to analyze more informative data in the future study.

35. Table S1: The "Read file size" column doesn't make much sense. Single file? Sum of R1 and R2? Interleaved? Compressed? It will be influenced by e.g. whether sequencing provider utilizes the 3rd comment line of every read. Consider deleting and using the extra horizontal space to provide the number of basepairs per sample or e.g. the raw number of reads pre-quality-trimming.

Answer) We revised the Supplementary Table S1 to provide the read file information including the number of base pairs and the number of paired reads per sample for pre-quality-trimming and post-quality-trimming.

Close
