# Supplementary material for: Antibiotic resistomes discovered in the gut microbiomes of Korean swine and cattle
Source: Gigascience. 2020 May 5;9(5):giaa043. doi: 10.1093/gigascience/giaa043 (PMC7317084; doi:10.1093/gigascience/giaa043)
Supplement: giaa043_Response_to_Reviewer_Comments_Revision_1 [file giaa043_response_to_reviewer_comments_revision_1.pdf]

# Author's Response To Reviewer Comments

Close

Reviewer #2: I am happy to find, that the authors have more or less addressed all my concerns. I have comments for some of their rebuttals and for some of the changes to the manuscript. Also, the authors / journal might want to specify in the paper title reflect that the scope is Korea, like the authors have now done throughout the manuscript.
Answer to author rebuttal:

1 Looks like a fine approach. What was the difference between the first and second filtering threshold? Could a single "70% pass" give the same result?

Answer) When we compared the number of reads in four samples with and without 70% similarity threshold, there was no difference.

3 I am not going to insist the authors use CODA methods, but as I explained further down, I have some specific objections to their (old and new) analysis, where I don't think it makes sense and could be helped using alternatives suggested by Gloor et al. Also as far as I can see, the transition to CLR is not meaningful when coupled with their chosen analysis.

Answer) Following the reviewer's helpful suggestion, we have performed the correlation analysis with SPARCC. The revised manuscript reports the results in line 396 and lines 112–113, as well as Supplementary Figure S1.

Specific comments:

L31: Language could be a bit more precise in conclusion and throughout. Does "more diverse" refer to a higher alpha diversity / evenness in samples from one livestock species or to a larger beta-diversity within one livestock species compared to the other?

Answer) We used the expression "more diverse" to explain that more genes are prevalent in swine than in cattle. Specifically, for the prevalent ARGs (prevalence > 50%), we found 32 genes in swine, while 19 genes in cattle. We revised the sentence to read "Antibiotic resistome was more pervasive in swine than in cattle. Specifically, prevalent ARGs (prevalence > 50%) were found more in swine than in cattle".

L92: On my request, the authors have now added the information on the actual sequencing instrument (HiSeq4000) which ENA also states. However, the MS also still refers to the HiSeqX. It also still says minimum was 110M PE reads. These discrepancies should be fixed.

Answer) Corrections were made accordingly.

L112: A negative correlation coefficient is given. Is it significant? Prevotella was very common, so it is not surprising there are lower proportions of other bacteria in samples with a lot of Prevotella. See further down, why the test probably does not yield meaningful results.

Answer) In response to the reviewer's suggestion, we performed the correlation analysis with SPARCC. We note that the proportion of Prevotella still showed a negative correlation with that of Subdoligranulum (r = –0.6457).

L169: Were the values normally distributed so the results of t-tests are valid and meaningful? If median cattle phenicol AMR was 0 RPKM, then it seems unlikely? If the authors log transform data (after zero replacement), they will get ALR values which are more likely to be normal.

Answer) The normality test showed that several ARGs including phenicol are not normally distributed. We thus removed the t-test results and just compared RPKM values for abundance between swine and cattle.

L382 "The genus proportion (>0.1%) was used for calculating Shannon index…". Is it correctly understood that rare genera were excluded? That will impact the diversity indexes and seems like it should be justified. I am not convinced its required, especially if the sample was also rarefied (a practice there is some controversy around: https://doi.org/10.1371/journal.pcbi.1003531).

Answer) In response to the reviewer's suggestion, we also calculated the index with the genus without any proportion threshold (Supplementary Figure S4D–F). The result showed the same pattern. The method section was also revised; please see lines 390–392.

L388: If the authors use rank-based correlation (Spearman), CLR transformation will not have any effect. If the authors want to implement some of the CODA principles from the papers, this is not a good place, when coupled with a rank-based correlation. Rank-based does not solve the issue of negative correlation bias. In the Gloor paper I linked (GB Gloor et al, 2017), they say e.g. the following: "There are several more rigorous approaches that can be applied to analyze correlation in microbiome datasets, including SPARCC (Friedman and Alm, 2012) and SPieCeasi (Kurtz et al., 2015), both of which assume a sparse data matrix, and the ϕ (Lovell et al., 2015) and ρ…"

Answer) In response to the reviewer's suggestion, we performed correlation analysis using SPARCC and revised the manuscript. Please see line 396 and lines 112–113, as well as Supplementary Figure S1.

Supplementary:

Some of the captions are very short and uninformative.
Answer) Corrections were made accordingly.

The authors claim they do not have estimates of the size of Korean pig and cattle production. Figure S5 has (antibiotic sales in mg/PCU + 1). I am not sure how the authors have managed to adjust the drug use to the size of animal production then. "1 PCU = 1 kilogram of biomass of different categories of livestock and slaughtered animals". This needs explaining and if it is not adjusted to production size / number of animals / meat produced / sold or similar, then than sub-plot can be removed. The lines being higher on the Y axis in one animal is then not very informative.

Answer) The caption for Figure S5(A) was corrected to read "the amount of antibiotics sold (ton)", which is consistent with Figure S3.

Close
