# Supplementary material for: Antibiotic resistomes discovered in the gut microbiomes of Korean swine and cattle
Source: Gigascience. 2020 May 5;9(5):giaa043. doi: 10.1093/gigascience/giaa043 (PMC7317084; doi:10.1093/gigascience/giaa043)
Supplement: giaa043_Reviewer_1_Report_Original_Submission — Arnaud Bridier -- 11/22/2019 Reviewed [file giaa043_reviewer_1_report_original_submission.pdf]

Reviewer Report

Title: Antibiotic resistomes discovered in the gut microbiomes of swine and cattle

Version: Original Submission **Date:** 11/22/2019

Reviewer name: Arnaud Bridier

Reviewer Comments to Author:

In the manuscript entitled "Antibiotic resistomes discovered in the gut mirobiomes of swine and cattle", Lim et al. applied deep sequencing and metagenomic analyses on 36 swine and 41 cattle feces from 25 feedlots in South Korea to characterize and compare prevalence and diversity of antibiotic resistance genes in these animals. Authors found that prevalence of resistance is higher in swines. They identified aminoglycoside resistance as the most prevalent both in cattle and swine but involving different gene families.

General comments

Nowadays, the topic of antimicrobial resistance (AMR) is becoming one of the most critical public health issues and it is crucial to better understand the distribution of resistance determinants and identify the reservoirs and the routes of dissemination involved in AMR emergence in a One Health approach. By performing shotgun metagenomics on a consequent number of samples (totals of 77 36 swine and 41 cattle feces metagenomes) collected nationwide, Lim et al., generated valuable data on a representative panel in this perspective. Sample information for swine and cattle are well presented in Table S2 and S3. All sequencing data were available at ENA (accession number PRJEB32496) and showed all a relevant sequencing depths for such resistome analyses. Interesting results are provided about the specific patterns of resistance in swine and cattle and with the identification of shared resistance gene sequence (for example tetQ and tetX) or specific sequence depending on animal species (tetM). Such results are important to better understand resistance gene prevalence and their dynamics among farm animals and generally in the environement.

The main limitation is that the manuscript in the current form remains too superficial in methodologies description and result discussion. First, bioinformatics and statistical methods performed on metagenomics data are not sufficiently detailed. For instance, there is also no clear description of the tools and statistics used for correlation studies between resistance classes and genus (which R package was used?) or for the network analysis. Thresholds and parameters used during metagenomics data processing (as for bacterial taxa identification with MetaPhlAn for instance) should also be added to the methods section to facilitate the reuse of the analysis for the reader. Please describe methodologies applied to metagenomics data and statistics with more details in the Methods section.

In addition, the presentation and interpretation of data could also be thorough. Results should be discussed in light of previous studies in an extensive manner. Some papers recently published on resistome studies in pig and/or cattle could be cited and used to confront results found here as for instance: PMID: 15066850; PMID: 30761096; PMID: 26952213; PMID: 31455230.

Specific comments

Concerning the analysis of bacterial composition, how the sample locations (farms) impact the

populations? There are especially 3 samples which are isolated on the bottom of the PCA (fig. 2). Are these samples were sampled in the same feedlot? Do the authors have some elements to explain such microbiome specificities?
The Spearman's correlation figure (fig. S1) is important and could be integrated in the core manuscript text rather than in supplementary materials.
Authors mentioned in Discussion that the study revealed a general correlation among antibiotic usages, resistance phenotypes, and resistome. This is a very interesting point but that should be supported by statistical analysis or at least with a dedicated figure summarizing and combining such data to clearly show this trend.
It would be interesting to have a discussion on the link between bacteria and resistance gene or, in other words, to investigate if resistance genes that are commonly found if swine and cattle, are carrying by the same bacteria in both animals.
In discussion, authors referred to a previous study: "…, tetracycline genes were found in higher rate in cattle than I swine (Figure 6), which is consistent with a previous study" but there is no reference.
There is no line numbers that complicate the review.
There are some typing errors along the paper, please correct. In addition, english revision of the text would improve clarity of the manuscript.
Some references to figure S3A and B were made in "Pervasive antibiotic resistance genes in the gut microbiome of swine and cattle" of Analysis section instead of referring to figure 5 (fig. S3 indeed correspond to the amounts of antibiotic sold.) Please correct.
Gene names should be figured in italic.

Methods

Are the methods appropriate to the aims of the study, are they well described, and are necessary controls included? Choose an item.

Conclusions

Are the conclusions adequately supported by the data shown? Choose an item.

Reporting Standards

Does the manuscript adhere to the journal's guidelines on minimum standards of reporting? Choose an item.

Choose an item.

Statistics

Are you able to assess all statistics in the manuscript, including the appropriateness of statistical tests used? Choose an item.

Quality of Written English

Please indicate the quality of language in the manuscript: Choose an item.

Declaration of Competing Interests

Please complete a declaration of competing interests, considering the following questions:

- Have you in the past five years received reimbursements, fees, funding, or salary from an organisation that may in any way gain or lose financially from the publication of this manuscript, either now or in the future?
- Do you hold any stocks or shares in an organisation that may in any way gain or lose financially from the publication of this manuscript, either now or in the future?
- Do you hold or are you currently applying for any patents relating to the content of the manuscript?
- Have you received reimbursements, fees, funding, or salary from an organization that holds or has applied for patents relating to the content of the manuscript?
- Do you have any other financial competing interests?
- Do you have any non-financial competing interests in relation to this paper?

If you can answer no to all of the above, write 'I declare that I have no competing interests' below. If your reply is yes to any, please give details below.

I declare that I have no competing interests' below.

I agree to the open peer review policy of the journal. I understand that my name will be included on my report to the authors and, if the manuscript is accepted for publication, my named report including any attachments I upload will be posted on the website along with the authors' responses. I agree for my report to be made available under an Open Access Creative Commons CC-BY license (http://creativecommons.org/licenses/by/4.0/). I understand that any comments which I do not wish to be included in my named report can be included as confidential comments to the editors, which will not be published.

Choose an item.

To further support our reviewers, we have joined with Publons, where you can gain additional credit to further highlight your hard work (see: https://publons.com/journal/530/gigascience). On publication of this paper, your review will be automatically added to Publons, you can then choose whether or not to claim your Publons credit. I understand this statement.

Yes Choose an item.
