# Supplementary material for: Antibiotic resistomes discovered in the gut microbiomes of Korean swine and cattle
Source: Gigascience. 2020 May 5;9(5):giaa043. doi: 10.1093/gigascience/giaa043 (PMC7317084; doi:10.1093/gigascience/giaa043)
Supplement: giaa043_Reviewer_2_Report_Original_Submission — Patrick Munk, Ph.D. -- 11/22/2019 Reviewed [file giaa043_reviewer_2_report_original_submission.pdf]

Reviewer Report

Title: Antibiotic resistomes discovered in the gut microbiomes of swine and cattle

Version: Original Submission **Date:** 11/22/2019

Reviewer name: Patrick Munk, Ph.D.

Reviewer Comments to Author:

GENERAL:
The study is primarily focused with characterizing 77 fecal samples from Korean cattle and swine through metagenomic sequencing. Analysis on both the resistome and general phylogentic distribution is performed and MIC panels are used on a collection of isolated E. coli.
The field work, lab work and bioinformatics analysis itself appears good, the network analysis of homologous genes is nice, but the downstream statistical analyses and interpretations need quite a bit of work. More details are also needed before the study could be considered reproducible. I recommend the authors add the following to the MS:

- Actual abundance estimation of the individual ARGs in their samples (see specific comments later down). Basically the equivalent of metaphlan, but for ARGs - Map the reads to an ARG database like ResFinder or CARD or your metagenome-assembled-determined ARGs to determine how abundant they are. See e.g. how we have done it, but there are a lot of ways to estimate how abundant the genes are: Munk, P., Knudsen, B. E., Lukjacenko, O., Duarte, A. S. R., Van Gompel, L., Luiken, R. E. C., … Aarestrup, F. M. (2018). Abundance and diversity of the faecal resistome in slaughter pigs and broilers in nine European countries. Nature Microbiology, 3(8), 898-908. https://doi.org/10.1038/s41564-018-0192-9
- Do testing on dissimilarity matrices based on the abundances - The authors can use e.g. permanova to see how much of the variation in their taxonomic and resistance abundances can be explained by e.g. host species and the specific farm. Because they have several animal samples from each farm, they can calculate if same-farm animals are more similar and if so, how much.
- Consider analyzing abundance matrices as compositional data (CODA). See recent work by e.g. Gloor and Calle on why this is appropriate and consider calculating e.g. alpha and beta diversities using the methods suggested by them. This could help especially with the authors current attempts of correlating different genera with each other, which is prone to spurious correlations.

Gloor, G. B., Macklaim, J. M., Pawlowsky-Glahn, V., & Egozcue, J. J. (2017). Microbiome datasets are compositional: And this is not optional. Frontiers in Microbiology, 8(NOV), 1-6. https://doi.org/10.3389/fmicb.2017.02224
Calle, M. L. (2019). Statistical Analysis of Metagenomics Data. Genomics & Informatics, 17(1), e6. https://doi.org/10.5808/GI.2019.17.1.e6
The written English in the article is not of high quality. In most places, I can guess the authors meaning, but in other places the meaning is uncertain. The manuscript would greatly benefit from a native English speaker going through and improving it.
The genome assemblies, predicted genes and their translations are all available on the FTP server, which is much appreciated. In the spirit of sharing, it would be nice to also make e.g. read counts and

calculated bacterial abundances /biom files from metaphlan avilable. These basically fit in small tab-delimited files or spreadsheets for people to easily review and analyse.
Lastly, line numbers would have been much appreciated during review!
SPECIFIC COMMENTS:
P2
The very first sentence of the abstract doesn't make sense to me. I think the authors mean that antimicrobial resistance genes are getting enriched in different reservoirs, not that the antibiotic molecules themselves are accumulating over time?
"deep sequencing on the gut microbiomes of 36 swine and 41 cattle in the farms,…" - I would already in this sentence mention that the scope of the study is specifically Korean farms.
"Aminoglycoside resistance is the most abundant in both swine and cattle" - This implies higher number of genes which was not determined. The authors looked at the ARG richness (how many different genes), rather than abundance.
P3
"antibiotic resistance genes are more frequently transferred from one bacteria to others in the gut microbiome" - not clear what this refers to. More frequently than what?
"An interesting observation is the presence of two…" - I think this might be phrased slightly odd. It is not odd/interesting/surprising that some ARGs are unique to one of the livestock species and vice versa. That is quite common in these kind of studies.
P4
"A total of 36 gut microbiome" - I think it should be "microbiomes" in plural throughout the manuscript.
"For each farm, three or four samples.." - I think explanations of sampling scheme, rationale etc. should be moved up to the "Data description" or keep it in methods, rather than having it in the "analysis" section.
"The genus-level compositions were significantly different in swine and cattle" - If the authors say "significant", I think the author should add the name of the test and p-value, e.g. "significant (p=0.023, permanova / anosim / other test)". This also applies to other places in the MS where something is claimed to be significant.
P5
"… were the major genus in Firmicutes" - genus is "genera" (the authors have correctly used "genera" in other places). Firmicutes (a phylum) should not be in italic.
"Several genera showed high correlations in cattle" - Is this relevant? Don't we expect some genera to correlate in abundance simply by chance? Do we have more or less than expected by chance?
"Treponema and Bifidobacterium were observed, but they constituted lower than 1% of the composition" (in cattle) - 2 sentences later, you say that Treponema range between 0% and 99% (!) in cattle. So I assume the first 1% refered to was the median? Looking at the variability and e.g. range of Treponema, I think it would be wise to establish whether something funny is going on. 99% Treponema sounds odd to me and it would be nice to hear the authors reflections on this. Also to see the metaphlan output available to verify independently.
"X was positively correlated with Y" - The authors should be aware that correlations between microbiome features can be hard to interpret because data is compositional - the flow cell can only sequence X DNA molecules that get read, so if bacterium A becomes more abundant, B gets displaced

giving a natural tendency towards negative correlations. The different read counts within a single sample can thus not be considered independent.

I strongly suggest re-naming "intra-individual diversity" to "alpha diversity" which is the common phrase of the within-sample distribution of organisms in ecology, also adopted by many microbiome studies.

"The average number of genera that constituted…" - I suggest calculating a more classic alpha diversity index (e.g. Shannon or Simpson diversity).

"Normalized by the million genes predicted in the microbiome (GPM)" - I think the authors way of quantifying AMR in the samples is not appropriate or at least should not stand alone. MEGAHIT is fine for metagenomic assembly, but the authors are very strict in requiring the full length gene gets assembled in each sample. That lowers their detection threshold many fold. For taxonomic assignment, they match the reads directly to references (using metaphlan). Their GPM is actually not an "abundance" in that it doesn't capture how much resistance there is in each sample, but rather the ARG richness relative to total gene richness.

The GPM is fine to report, but without a relative abundance also, it is akin to omitting to report how much there is of each genera in their samples.

Therefore, I think the authors should also map reads directly to ARGs and account for differences in gene lengths and sequencing depth differences. If they then choose the conservative approach of only mapping reads to the genes they manage to assemble in some of their samples, that is okay. But I would suggest mapping their reads directly to a database such as ResFinder or CARD and analyzing the output.

"It implies that these six classes exist in more than half the population" - I do not understand this inference and would like it explained better.

"Moreover, the resistance rates were relatively lower…" - Does this refer to the proportion of animals in which the authors were able to assemble a gene of x phenotype?

P6

"Among the three gene families…" - The term "gene family" is used inconsistently. In one sentence it is used to refer to all aminoglycoside nucleotidyltransferases, in the next it refers to ANT(6) genes.

"The overall abundance of swine is evidently higher than that of cattle" - I assume abundance of ARGs is meant and not the actual pigs?

P9

When the authors say something "correlated", I think they should make it clear whether a correlation test was performed or there was an apparent association was determined visually.

P10

"Low-quality reads were removed using Sickle" - How was the program run / with what parameters (same goes for megahit)? Is it correctly understood that entire reads were removed, rather than trimming to the high-quality parts of the reads? An example command would be nice for reproducibility. Were singleton reads kept if the mate was discarded? I know MEGAHIT can accommodate a combination of single and PE reads, but the details are not clear.

"An average of 160 M paired-reads (ranging between 110 M and 229 M)" - This does not agree with the supplementary Table S1. There, I see e.g. a line with 26M trimmed read pairs (sample C_34). Is this average, perhaps calculated per farm instead of per sample? If so, it should be changed to per sample/individual fecal sample.

It would be good to also mention what DNA library preparation kit was used for the sequencing:

specifically, did it include a PCR amplification step?
P11
"Host contamination was removed by discarding the reads…" - What software, version, arguments was used against what exact NCBI accessions to complete this task?
OTHER
On ENA, it seems like the reads are annotated as coming from a HiSeq4000 instrument. If so, should the manuscript reflect that too?
FIGURES AND TABLES:
Figure1: Not strictly required, but I think a different plotting program that allowed less white in-between the bars would make the figure much more readable.
Figure S2: It seems like a lot is wrong with the caption and/or plots. The first sentence indicates the overall plot contains phylum-level data, which is not the case. The sub-plots are wrongly referenced: (A) and (B) are both family-level taxonomy, so I assume (B) is Family in cattle, rather than species in swine? Also (A) and (B) seem to be entirely "family", while (C) and (D) are not really "species" , but some mixture of families, genera and species (see Peptostreptococaecae, Alistipes and E. coli in (D)).
Figure S3: Are there some kind of production values available for Korea that the authors could use to account for how much biomass/animal is consuming the antibiotics? The raw tonnage can be misleading in case the country prudced 1000-fold more of one type of meat
Table S1: The "Read file size" column doesn't make much sense. Single file? Sum of R1 and R2? Interleaved? Compressed? It will be influenced by e.g. whether sequencing provider utilizes the 3rd comment line of every read. Consider deleting and using the extra horizontal space to provide the number of basepairs per sample or e.g. the raw number of reads pre-quality-trimming.

Methods

Are the methods appropriate to the aims of the study, are they well described, and are necessary controls included? Choose an item.

Conclusions

Are the conclusions adequately supported by the data shown? Choose an item.

Reporting Standards

Does the manuscript adhere to the journal's guidelines on minimum standards of reporting? Choose an item.

Choose an item.

Statistics

Are you able to assess all statistics in the manuscript, including the appropriateness of statistical tests used? Choose an item.

Quality of Written English

Please indicate the quality of language in the manuscript: Choose an item.

Declaration of Competing Interests

Please complete a declaration of competing interests, considering the following questions:

- Have you in the past five years received reimbursements, fees, funding, or salary from an organisation that may in any way gain or lose financially from the publication of this manuscript, either now or in the future?
- Do you hold any stocks or shares in an organisation that may in any way gain or lose financially from the publication of this manuscript, either now or in the future?
- Do you hold or are you currently applying for any patents relating to the content of the manuscript?
- Have you received reimbursements, fees, funding, or salary from an organization that holds or has applied for patents relating to the content of the manuscript?
- Do you have any other financial competing interests?
- Do you have any non-financial competing interests in relation to this paper?

If you can answer no to all of the above, write 'I declare that I have no competing interests' below. If your reply is yes to any, please give details below.

I declare that I have no competing interests

I agree to the open peer review policy of the journal. I understand that my name will be included on my report to the authors and, if the manuscript is accepted for publication, my named report including any attachments I upload will be posted on the website along with the authors' responses. I agree for my report to be made available under an Open Access Creative Commons CC-BY license (http://creativecommons.org/licenses/by/4.0/). I understand that any comments which I do not wish to be included in my named report can be included as confidential comments to the editors, which will not be published.

Choose an item.

To further support our reviewers, we have joined with Publons, where you can gain additional credit to further highlight your hard work (see: https://publons.com/journal/530/gigascience). On publication of this paper, your review will be automatically added to Publons, you can then choose whether or not to claim your Publons credit. I understand this statement.

Yes Choose an item.
