# Supplementary material for: Antibiotic resistomes discovered in the gut microbiomes of Korean swine and cattle
Source: Gigascience. 2020 May 5;9(5):giaa043. doi: 10.1093/gigascience/giaa043 (PMC7317084; doi:10.1093/gigascience/giaa043)
Supplement: giaa043_Reviewer_2_Report_Revision_1 — Patrick Munk, Ph.D. -- 3/12/2020 Reviewed [file giaa043_reviewer_2_report_revision_1.pdf]

Reviewer Report

Title: Antibiotic resistomes discovered in the gut microbiomes of swine and cattle

Version: Revision 1 **Date:** 3/12/2020

Reviewer name: Patrick Munk, Ph.D.

Reviewer Comments to Author:

I am happy to find, that the authors have more or less addressed all my concerns. I have comments for some of their rebuttals and for some of the changes to the manuscript. Also, the authors / journal might want to specify in the paper title reflect that the scope is Korea, like the authors have now done throughout the manuscript.

Answer to author rebuttal:

1 Looks like a fine approach. What was the difference between the first and second filtering threshold? Could a single "70% pass" give the same result?

3 I am not going to insist the authors use CODA methods, but as I explained further down, I have some specific objections to their (old and new) analysis, where I don't think it makes sense and could be helped using alternatives suggested by Gloor et al. Also as far as I can see, the transition to CLR is not meaningful when coupled with their chosen analysis.

Specific comments:

L31: Language could be a bit more precise in conclusion and throughout. Does "more diverse" refer to a higher alpha diversity / evenness in samples from one livestock species or to a larger beta-diversity within one livestock species compared to the other?

L92: On my request, the authors have now added the information on the actual sequencing instrument (HiSeq4000) which ENA also states. However, the MS also still refers to the HiSeqX. It also still says minimum was

110M PE reads. These discrepancies should be fixed.

L112: A negative correlation coefficient is given. Is it significant? Prevotella was very common, so it is not surprising there are lower proportions of other bacteria in samples with a lot of Prevotella. See further down, why the test probably does not yield meaningful results.

L169: Were the values normally distributed so the results of t-tests are valid and meaningful? If median cattle phenicol AMR was 0 RPKM, then it seems unlikely? If the authors log transform data (after zero replacement), they will get ALR values which are more likely to be normal.

L382 "The genus proportion (>0.1%) was used for calculating Shannon index…". Is it correctly understood that rare genera were excluded? That will impact the diversity indexes and seems like it should be justified. I am not convinced its required, especially if the sample was also rarefied (a practice there is some controversy around: https://doi.org/10.1371/journal.pcbi.1003531).

L388: If the authors use rank-based correlation (Spearman), CLR transformation will not have any effect. If the authors want to implement some of the CODA principles from the papers, this is not a good place, when coupled with a rank-based correlation. Rank-based does not solve the issue of negative correlation bias. In the Gloor paper I linked (GB Gloor et al, 2017), they say e.g. the following:

"There are several more rigorous approaches that can be applied to analyze correlation in microbiome datasets, including SPARCC (Friedman and Alm, 2012) and SPieCeasi (Kurtz et al., 2015), both of which assume a sparse data matrix, and the Ï• (Lovell et al., 2015) and Ï•…"
Supplementary:
Some of the captions are very short and uninformative.
The authors claim they do not have estimates of the size of Korean pig and cattle production. Figure S5 has (antibiotic sales in mg/PCU + 1). I am not sure how the authors have managed to adjust the drug use to the size of animal production then. "1 PCU = 1 kilogram of biomass of different categories of livestock and slaughtered animals". This needs explaining and if it is not adjusted to production size / number of animals / meat produced / sold or similar, then than sub-plot can be removed. The lines being higher on the Y axis in one animal is then not very informative.

Methods

Are the methods appropriate to the aims of the study, are they well described, and are necessary controls included? Choose an item.

Conclusions

Are the conclusions adequately supported by the data shown? Choose an item.

Reporting Standards

Does the manuscript adhere to the journal's guidelines on [minimum standards of reporting?] Choose an item.

Choose an item.

Statistics

Are you able to assess all statistics in the manuscript, including the appropriateness of statistical tests used? Choose an item.

Quality of Written English

Please indicate the quality of language in the manuscript: Choose an item.

Declaration of Competing Interests

Please complete a declaration of competing interests, considering the following questions:

- Have you in the past five years received reimbursements, fees, funding, or salary from an organisation that may in any way gain or lose financially from the publication of this manuscript, either now or in the future?

- Do you hold any stocks or shares in an organisation that may in any way gain or lose financially from the publication of this manuscript, either now or in the future?
- Do you hold or are you currently applying for any patents relating to the content of the manuscript?
- Have you received reimbursements, fees, funding, or salary from an organization that holds or has applied for patents relating to the content of the manuscript?
- Do you have any other financial competing interests?
- Do you have any non-financial competing interests in relation to this paper?

If you can answer no to all of the above, write 'I declare that I have no competing interests' below. If your reply is yes to any, please give details below.

I declare that I have no competing interests

I agree to the open peer review policy of the journal. I understand that my name will be included on my report to the authors and, if the manuscript is accepted for publication, my named report including any attachments I upload will be posted on the website along with the authors' responses. I agree for my report to be made available under an Open Access Creative Commons CC-BY license (http://creativecommons.org/licenses/by/4.0/). I understand that any comments which I do not wish to be included in my named report can be included as confidential comments to the editors, which will not be published.

Choose an item.

To further support our reviewers, we have joined with Publons, where you can gain additional credit to further highlight your hard work (see: https://publons.com/journal/530/gigascience). On publication of this paper, your review will be automatically added to Publons, you can then choose whether or not to claim your Publons credit. I understand this statement.

Yes Choose an item.
